# Electrochemical Late-Stage Functionalization

**DOI:** 10.1021/acs.chemrev.3c00158

**Published:** 2023-09-26

**Authors:** Yulei Wang, Suman Dana, Hao Long, Yang Xu, Yanjun Li, Nikolaos Kaplaneris, Lutz Ackermann

**Affiliations:** Institut für Organische und Biomolekulare Chemie and Wöhler Research Institute for Sustainable Chemistry (WISCh), Georg-August-Universität, Göttingen 37077, Germany

## Abstract

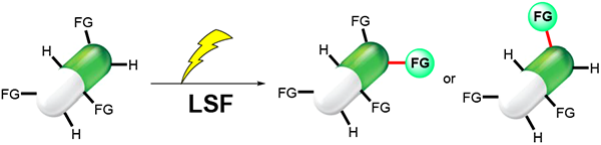

Late-stage functionalization (LSF) constitutes a powerful strategy for the assembly or diversification of novel molecular entities with improved physicochemical or biological activities. LSF can thus greatly accelerate the development of medicinally relevant compounds, crop protecting agents, and functional materials. Electrochemical molecular synthesis has emerged as an environmentally friendly platform for the transformation of organic compounds. Over the past decade, electrochemical late-stage functionalization (eLSF) has gained major momentum, which is summarized herein up to February 2023.

## 1 Introduction

**Scheme 1:**
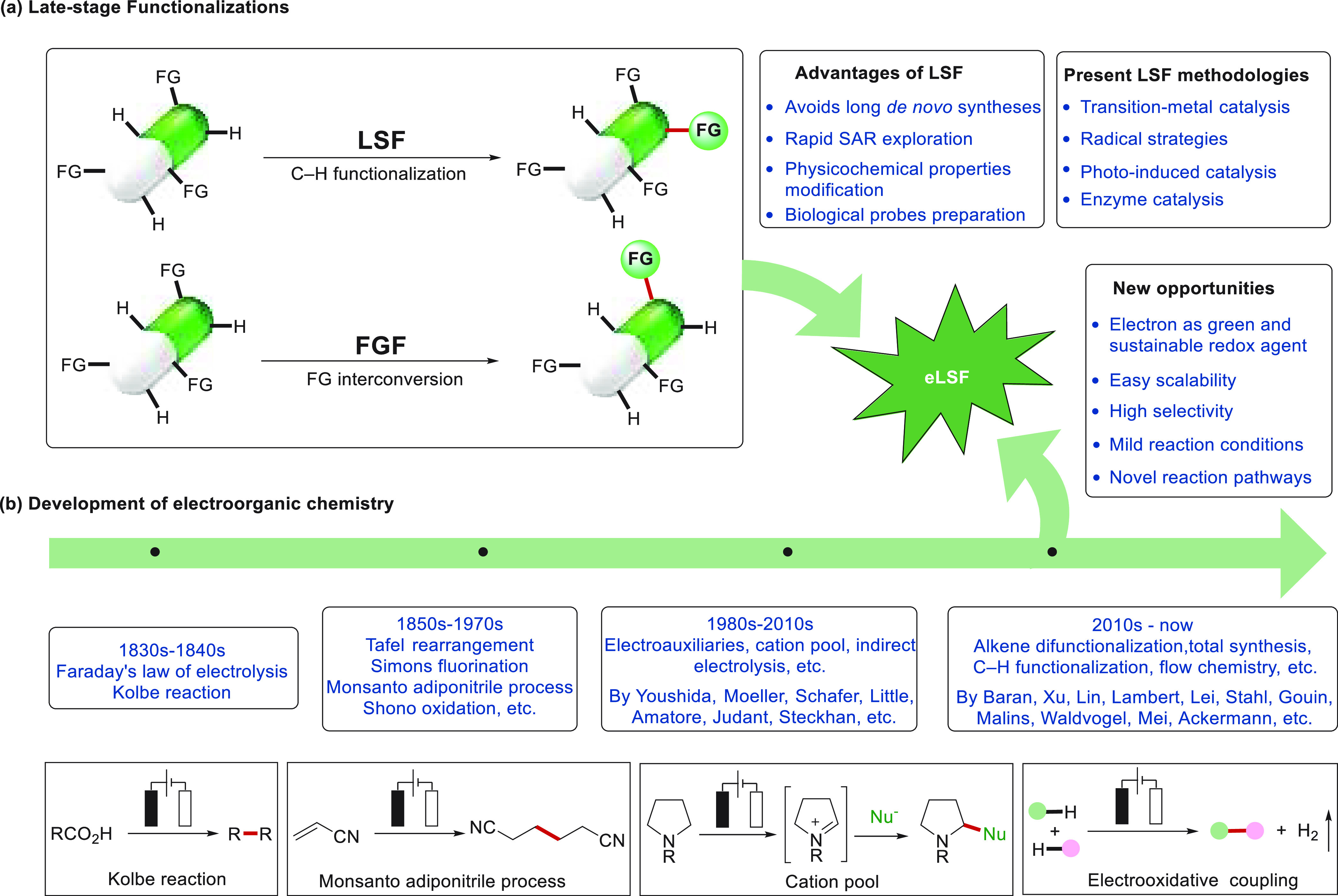
Opportunities for Electrochemical Late-Stage Functionalization Strategies in Drug Discovery

The direct and site-selective late-stage diversification of structurally complex molecules is of great potential for drug discovery, materials science, crop protection, and other areas.^1−8^ This approach avoids a complete *de novo* synthesis of a target molecule, enables the rapid creation of large compound libraries, and hence offers the promise of a fast exploration of structure–activity relationships (SARs). Thereby, an improvement of pharmacokinetics properties as well as physicochemical drug characteristics, such as potency, stability, solubility, and selectivity, is frequently viable.^9^ The most synthetically useful late-stage functionalization (LSF) strategy is often the direct installation of fluorophores or small, noninvasive groups—*inter alia* methyl, hydroxyl, chloro, fluoro, or trifluoromethyl—with a selectivity control at a specific site of an existing biologically relevant molecule. The introduction of a small group can dramatically affect the bioactivity profiles of a structurally complex pharmaceutical molecule. For instance, Pfizer found that the installation of a methyl group to a morpholine-containing compound of mineralocorticoid receptor (MR) agonist, gave rise to a 45-fold potency increase.^10^ In the past decade, a large number of LSF approaches have been developed, including metal-catalyzed transformations,^11−21^ visible-light-induced photocatalysis^22−34^ and enzyme catalysis,^35−43^ among others (Scheme 1a).^44−48^

Electrochemical synthesis is a robust tool for sustainable molecular syntheses since it generally features mild reaction conditions, high selectivities, and facile scalability by flow techniques (Scheme 1b). Electrosynthesis has a history of nearly 200 years that can be traced back to Faraday’s conversion of acetic acid in the 1830s^49^ and Kolbe’s electrochemical decarboxylative dimerization,^50^ as well as industrially conducted processes, including the Simons fluorination process,^51^ the Monsanto adiponitrile process,^52^ and the Shono oxidation.^53^ Yoshida introduced the concepts of electroauxiliaries and *cation pool* to increase the electrosynthesis viability in the late 20th century.^54−56^ Meanwhile, Steckhan elegantly formalized the principles of indirect electrolysis, which thereafter brought forth numerous mediator-driven processes.^57,58^ Subsequent key achievements on the direct electrolysis were made by Schäfer,^59^ Lund,^60^ Little,^61−63^ Moeller,^64^ Jutand,^65^ and Amatore^66^ around the 21st century. On the basis of these pioneering contributions, electro-organic synthesis reemerged in the past decade, with major contributions by Baran,^67^ Xu,^68^ Lei,^69^ Ackermann,^70^ Lambert,^71^ Lin,^72^ Gouin,^73^ Waldvogel,^74^ Stahl,^75^ Malins,^76^ and Mei,^77^ among others.^78−85^ Thus, major advances have been achieved in various fields, including C–H activation, reductive cross-electrophile coupling, alkene difunctionalization, nitrogen-centered radical mediated chemistry, total synthesis, and the LSF of natural products and medicinally relevant molecules (Scheme 1b).

Over the years, a variety of articles have been published that summarized the impressive advances made in the field of electro-organic synthesis^86−106^ and late-stage functionalization,^1−8,46,107−113^ respectively. In contrast, comprehensive reviews of electrochemical late-stage functionalization has remained elusive.^76^ Thus, we herein aim at providing an overview on the advances in the area of electrochemical late-stage functionalization (eLSF), with a topical focus on biorelevant compounds. Notably, we define eLSF reactions as the direct, site-selective, and chemoselective functionalization of C–H bonds or endogenous functional groups on biologically relevant molecules, natural products, pharmaceuticals, or structurally complex molecules consisting of these moieties to provide their analogues. Such alterations may have the capacity to modulate properties, in a beneficial manner of binding affinity, drug metabolism, or pharmacokinetic properties, generally without loss of or even with an enhancement of the drug’s biological activity.

## 2 eLSF of C–H Bonds

Over the past decade, the merger of electrocatalysis with C–H activation has revolutionized the art of molecular synthesis. Particularly, a number of these electrochemical C–H functionalization techniques have been successfully applied for late-stage diversification of natural products and pharmaceuticals, and these key developments afforded tremendous opportunities in drug discovery programs.

### 2.1 eLSF of C(sp^2^)–H Bonds

#### 2.1.1 Late-Stage C(sp^2^)–H Carbonization

Among numerous strategies for late-stage functionalization, the methylation reaction plays a unique role in the modulation of bioactive molecules.^6^ The incorporation of a simple methyl group can dramatically improve their potency by enhancing lipophilicity, metabolic stability, and binding interactions, among others, which are collectively referred as the “magic methyl effect”. A literature survey of >2000 cases revealed that 8% of methyl installations led to a > 10-fold potency boost, and >100-fold activity increases in 0.4% of cases.^9^ Consequently, and despite indisputable progress, new synthetic methylation strategies, particularly direct C–H methylation, are highly sought after.

**Scheme 2:**
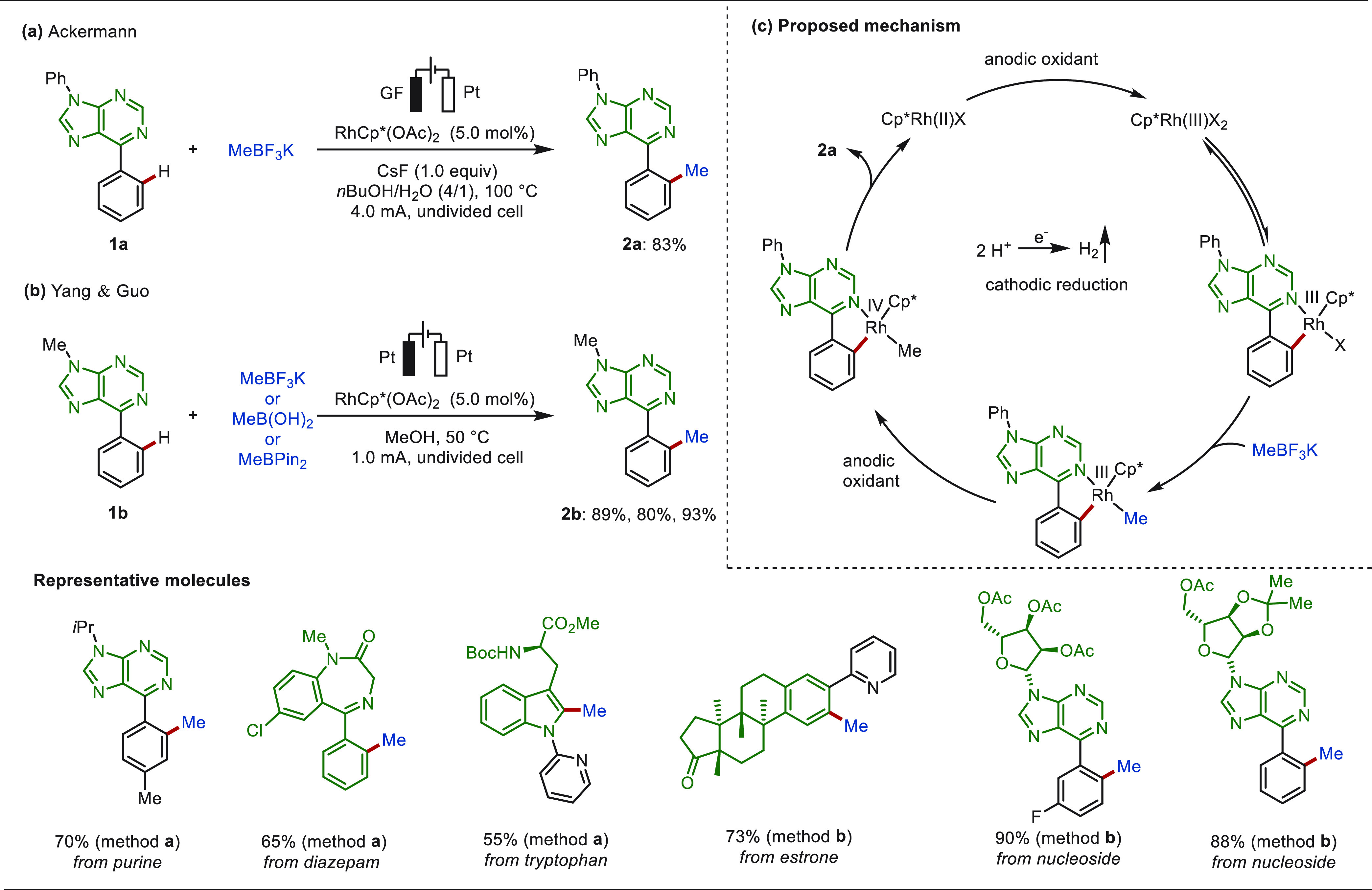
Electrochemical Rhodium-Catalyzed Late-Stage C(sp^2^)–H Methylation

In 2017, Mei and co-workers first disclosed the electrochemical C(sp^2^)–H methylation via anodic oxidation with MeBF_3_K as the methyl source under the catalysis of Pd(OAc)_2_, offering an alternative methylation strategy to conventional method that requires strong chemical oxidants.^114,115^ In 2022, the Ackermann group reported on an electrochemical *ortho* C(sp^2^)–H methylation of N-heteroarenes with the aid of RhCp*(OAc)_2_ as a catalyst and MeBF_3_K as the methyl source in a mixture of *n*BuOH and H_2_O (Scheme 2a).^116^ This approach proceeded in a user-friendly undivided cell setup and has been successfully applied to various biologically molecules, including purines, diazepam, and amino acids with high levels of site- and monoselectivity. Shortly thereafter, Guo and co-workers described a similar transformation in MeOH. Besides MeBF_3_K, MeB(OH)_2_ and MeBPin were further used as coupling partners for this rhodaelectro-catalyzed C–H methylation (Scheme 2b).^117^ Hence, the eLSF of a variety of bioactive architectures including purines, estrone, nucleosides, and nucleotides were achieved under mild reaction conditions. Current was the only oxidant for this catalysis. Mechanistic studies indicated that an anodic-oxidation-induced reductive elimination occurred within a rhodium(III/IV/II) regime. Meanwhile, H_2_ was released as the byproduct at the cathode (Scheme 2).

**Scheme 3:**
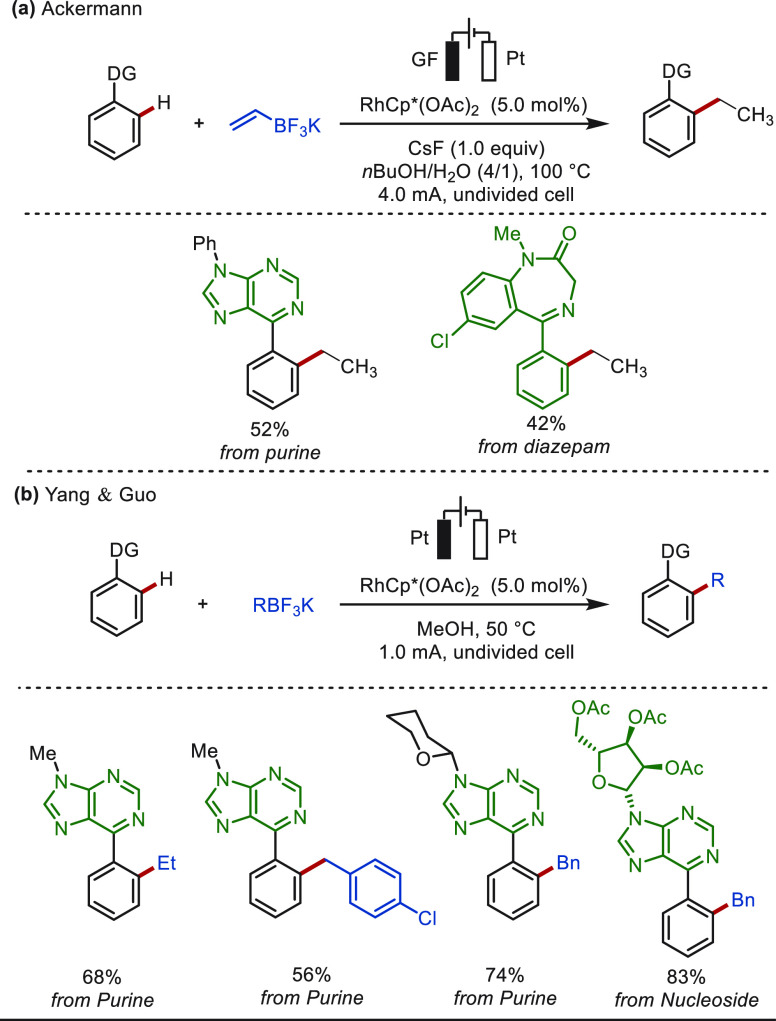
Electrochemical Rhodium-Catalyzed Late-Stage C(sp^2^)–H Alkylation

In addition, the monoselective C–H ethylation of purines and diazepam was achieved by the Ackermann group with VinBF_3_K by paired electrolysis, in which the reduction of in situ generated vinylated products takes place at the cathode to afford the ethylated products (Scheme 3a).^116^ Under Guo’s reaction conditions, different alkylation agents was examined for the LSF of purine derivatives (Scheme 3b).^117^ While potassium ethyltrifluoroborate and potassium benzylic trifluoroborates were identified as suitable substrates, giving the desired alkylation products, other alkyltrifluoroborates (*n*butyl, trifluoromethyl, and cyclohexyl) were unsuccessful.

Approximately 20% of commercial drugs contain at least one fluorine atom.^118^ The introduction of fluorine-containing groups leads to a significant boost in the potency of bioactive compounds.^119,120^ Particularly, trifluoromethylated compounds are in high demand in pharmaceutical industries and medicinal chemistry, since they can display unique lipophilicity and bioactivity.^121,122^ Consequently, direct C–H trifluoromethylation occupies an important position in terms of LSF.

**Scheme 4:**
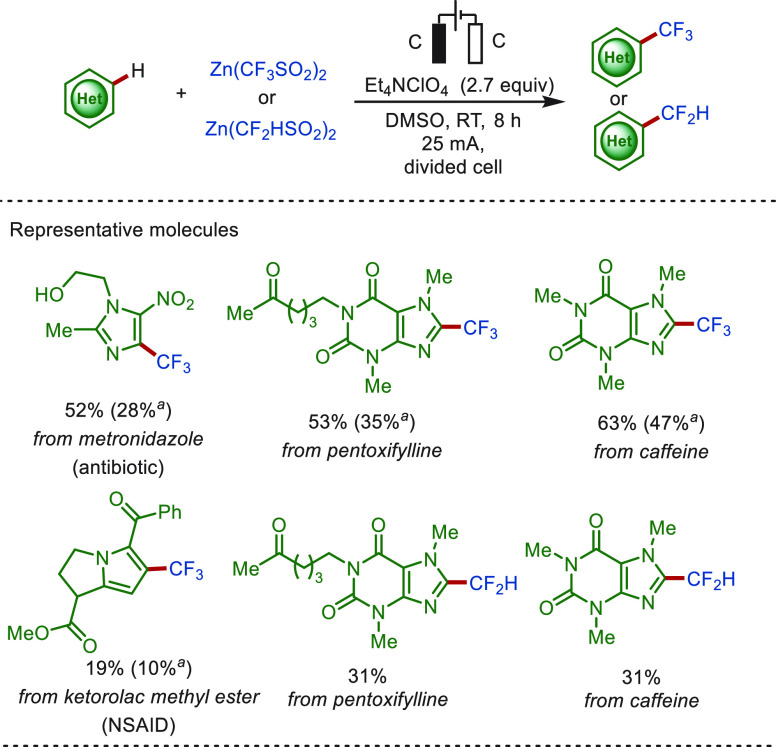
Electrochemical Controlled C(sp^2^)–H Late-Stage Trifluoromethylation and Difluoromethylation

In 2014, Baran and co-workers reported an electrochemical C(sp^2^)–H trifluoromethylation of heterocyclics with Zn(CF_3_SO_2_)_2_ under constant current electrolysis (Scheme 4).^123^ This approach featured mild reaction conditions with high site-selectivity and offers a wide application for late-stage trifluoromethylation of molecular architectures, including metronidazole, pentoxifylline, caffeine, and ketorolac methyl ester. Notably, this strategy resulted in significantly improved yields compared to the traditional method using *tert*-butyl hydroperoxide (TBHP) as the radical initiator and oxidant. Mechanistic studies indicated a controlled electron transfer at the anode, giving a sulfinate radical, which was rapidly converted to fluoroalkyl radical by cleavage and releasing SO_2_. Difluoromethylation of complex molecules was also achieved with Zn(CF_2_HSO_2_)_2_ at an elevated temperature of 60 °C, albeit in lower yield because of the poor reactivity of the CF_2_H radical with heterocycles.^123^

**Scheme 5:**
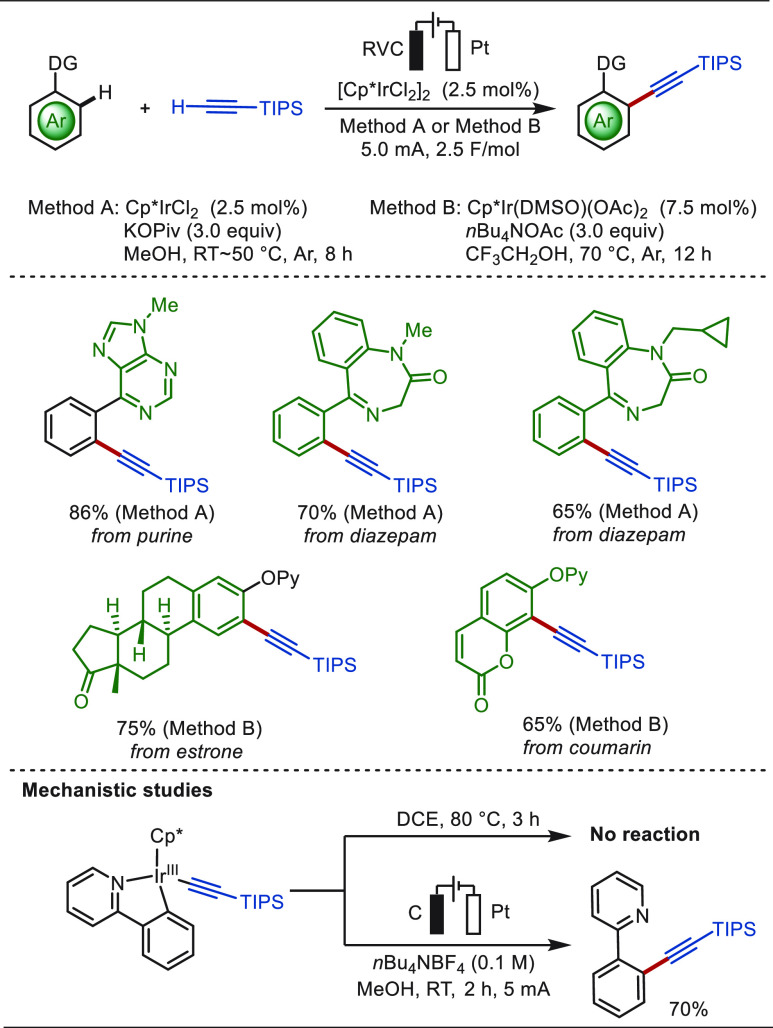
Electrochemical Iridium-Catalyzed Late-Stage C(sp^2^)–H Alkynylation

The transition-metal-catalyzed C–H alkynylation is a practical strategy in synthetic chemistry to install the versatile alkyne as a (transient) functional group.^124−126^ In 2020, Shi and Xie reported an electrochemical iridium-catalyzed directed C(sp^2^)–H alkynylation with terminal alkyne in an undivided cell (Scheme 5).^127^ Here, anodic oxidation was enabled by an iridium(III) intermediate to promote reductive elimination, affording the desired coupling products in excellent to good yields without the use of exogenous chemical oxidants. This transformation was amenable to various N-based directing groups, such as pyridyl, pyrazolyl, and isoquinolyl, enabling a high atom economy with H_2_ as the byproduct. The success of installing an alkyne on complex bioactive molecules, including derivatives of purine, diazepam, estrone, and coumarin, highlighted the potential application of this approach in late-stage functionalization of pharmaceuticals.

**Scheme 6:**
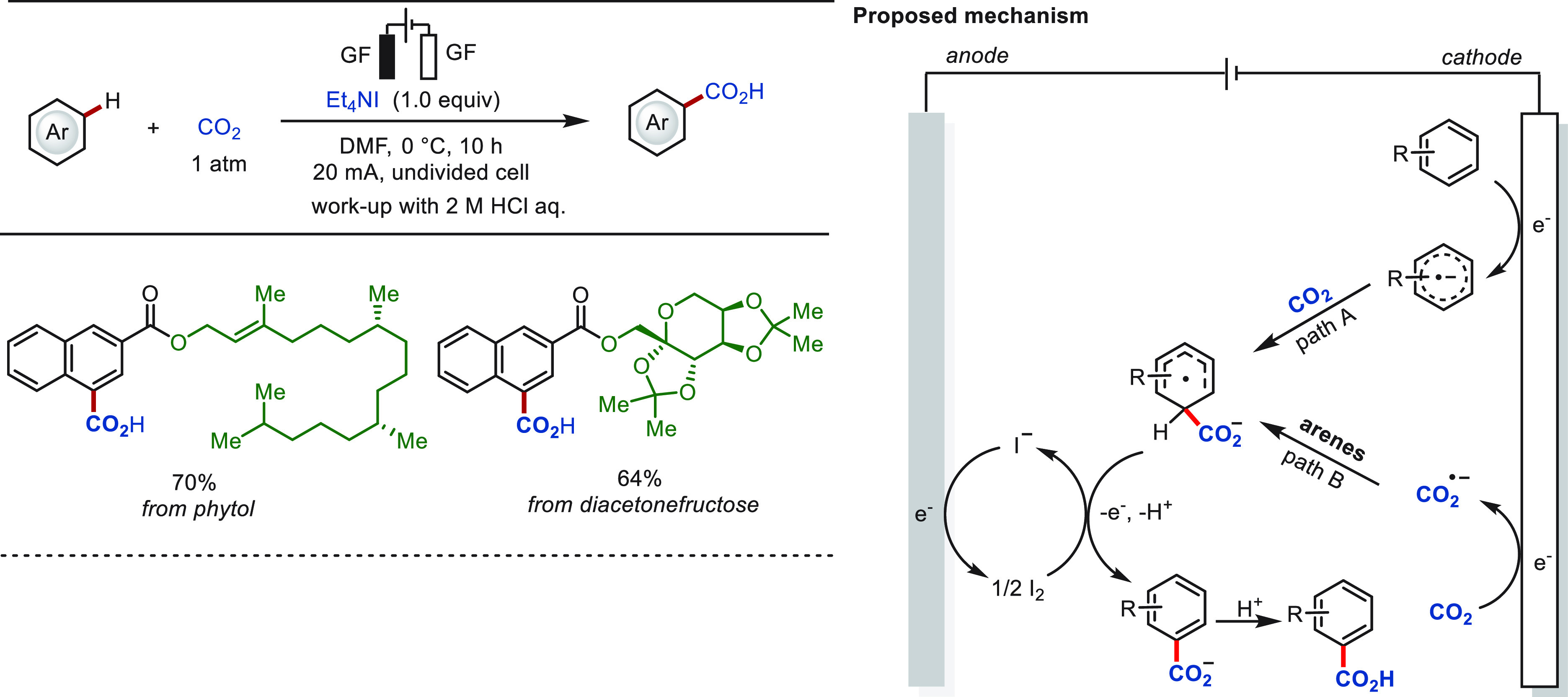
Electrochemical Late-Stage C(sp^2^)–H Carboxylation with CO_2_

CO_2_ is an abundant C-1 source that has been widely used in electrochemical transformations to construct diverse carboxylic acid compounds or their derivatives.^128−144^ In 2022, the Qiu group reported a direct aromatic C(sp^2^)–H carboxylation approach with CO_2_ to access synthetically useful aryl carboxylic acids (Scheme 6).^145^ This transformation proceeded in an undivided cell, displaying high site selectivity and chemoselectivity and obviating the use of a transition-metal catalyst. An array of challenging arenes, including electron-deficient naphthalenes, as well as heteroarenes such as pyridines and substituted quinolines, proved to be suitable substrates. The late-stage carboxylation of bioactive molecules derived from phytol and diacetonefructose was achieved efficiently. For a substrate with a less negative reduction potential, the process commences with the arene reduction at the cathode to form the corresponding radical anion. By contrast, for a substrate with a more negative reduction potential than that of CO_2_, the transformation starts with the CO_2_ reduction to a CO_2_ radical anion.

#### 2.1.2 Late-Stage C(sp^2^)–H Oxygenation

The direct hydroxylation of arene C(sp^2^)–H bonds is a highly sought-after transformation in the field of LSF, since the introduction of a small −OH group can dramatically improve *inter alia* the water solubility of the drug molecules.^38^ However, the controlled electrochemical hydroxylation of the aromatic C–H bond is challenging to achieve given the high propensity of the phenol to undergo overoxidation. To attenuate the overoxidation issue, trifluoroacetic acid (TFA) was taken into consideration as the oxygen donor to first generate aryl trifluoroacetate intermediates, followed by hydrolysis to release the hydroxylated products.^146,147^ Although this approach seems promising, the viable methods are limited to a few examples of structurally simple and electron-deficient/neutral arenes. Electron-rich arenes still typically suffer from overoxidation and self-coupling side reactions, and hence cannot efficiently converted to phenols.^148^

**Scheme 7:**
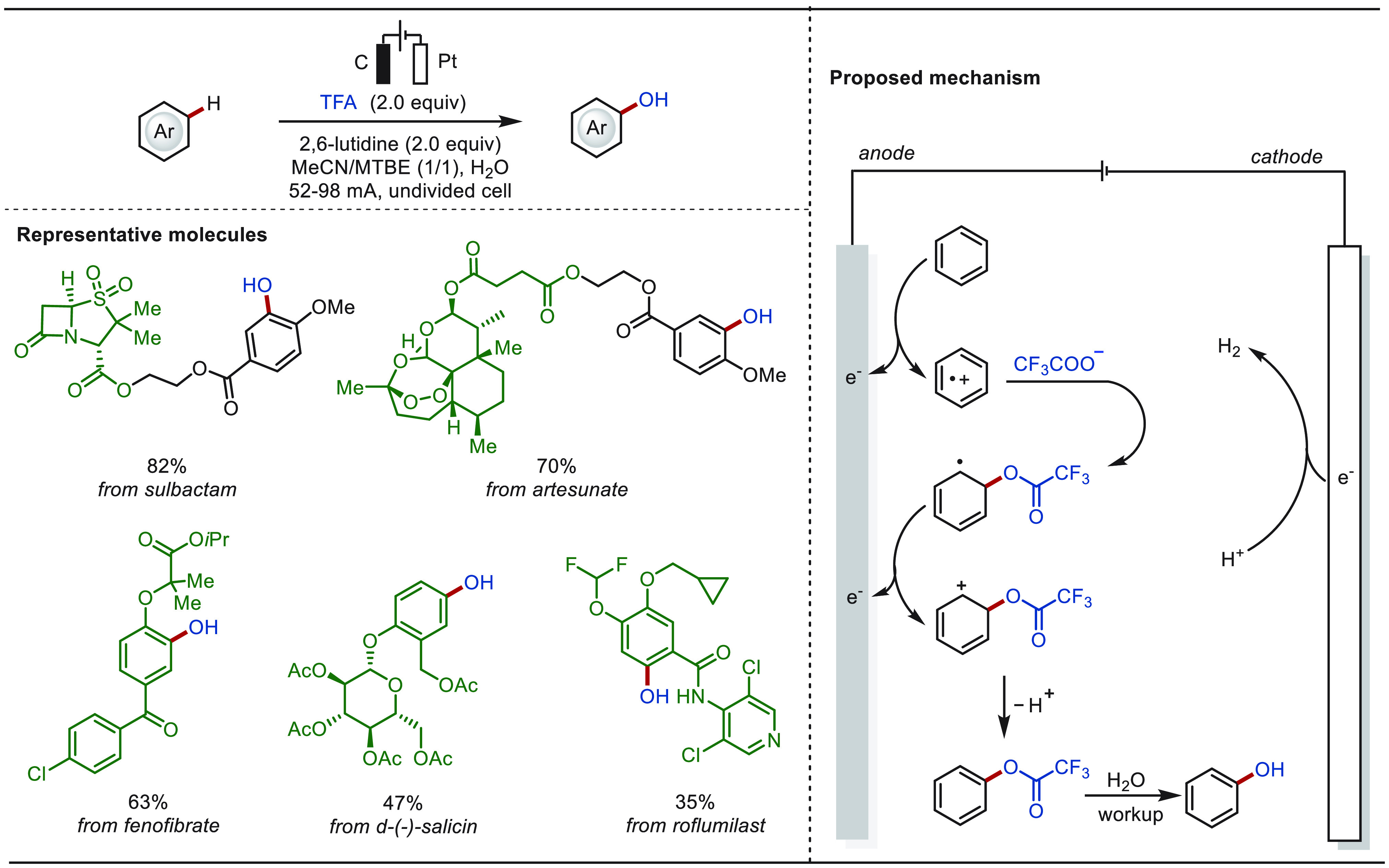
Electrochemical Late-Stage C(sp^2^)–H Hydroxylation

Continuous-flow electrochemical microreactors have the potential to increase the reaction efficiency and reducing overoxidation.^149−155^ In this context, the Xu group elegantly realized electrochemical C(sp^2^)–H hydroxylation of diverse arenes with high efficiency and selectivity in a continuous flow electrochemical microreactor (Scheme 7).^156^ The approach proceeded under mild conditions without chemical oxidants or transition-metal catalysts, featuring a broad scope of arenes with diverse electronic properties. The overoxidation reaction was greatly inhibited. The eLSF of a number of natural products and drug derivatives was achieved in an efficient and selective manner.

**Scheme 8:**
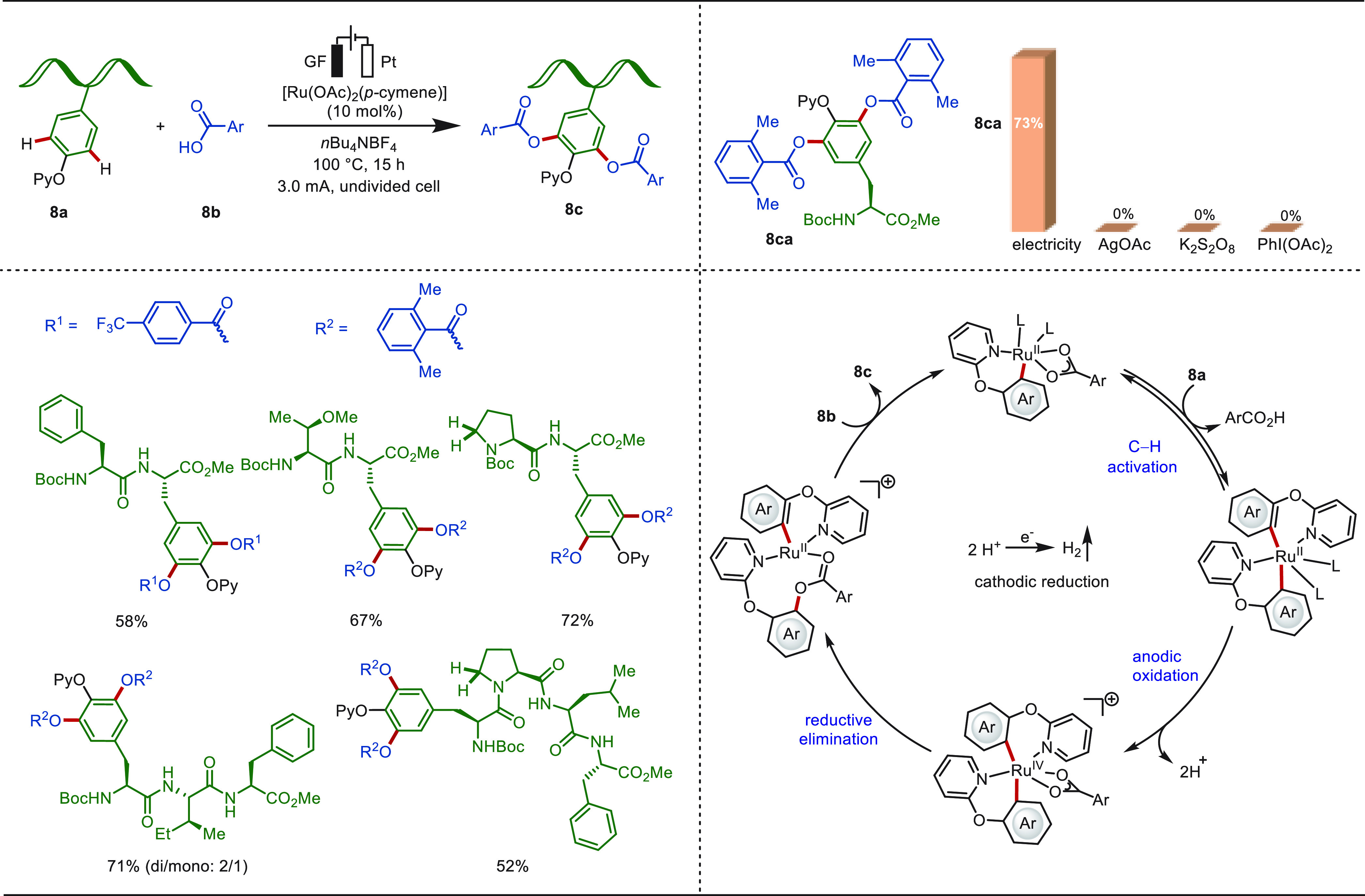
Electrochemical Late-Stage C–H Acyloxylation of Tyrosine-Containing Peptides Reproduced with permission from ref (162). Copyright 2022, Royal Society of Chemistry.

Besides the direct electrolysis method, the merger of electrocatalysis with organometallic C–H activation provides another sustainable strategy for the oxygenation of the aromatic C(sp^2^)–H bond.^157−162^ The Ackermann group has previously reported rhodaelectro- and ruthenaelectro-catalyzed hydroxylations of diverse arenes with TFA.^159,161^ Very recently, the same group achieved the ruthenaelectro-catalyzed late-stage C(sp^2^)–H acyloxylation of tyrosine-containing peptides with various aromatic acids (Scheme 8).^162^ Notably, attempted transformations with chemical oxidants, including AgOAc, K_2_S_2_O_8_, and PhI(OAc)_2_, proved to be ineffective — a strong testament to the robust nature of the electrochemical approach. A variety of di-, tri-, and tetrapeptides were efficiently acyloxylated without epimerization of the otherwise sensitive peptides. Remarkably, this electro-oxidative regime bypassed Shono-type manifolds even when employing proline-containing peptides. Mechanistic studies indicated that *p*-cymene dissociated during the catalytic cycle and the catalyst underwent a ruthenium II/IV regime likely involving a bis-cyclometalated complex intermediate.

**Scheme 9:**
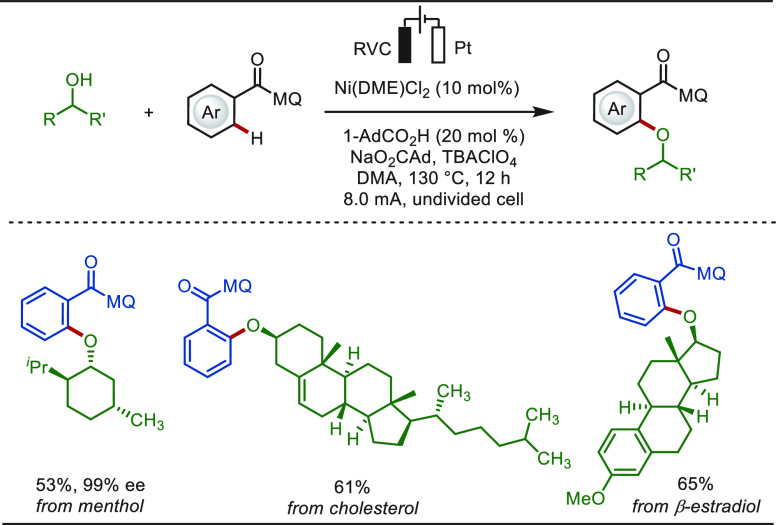
Electro-oxidative C–H Alkoxylation of Arenes with Secondary Alcohols

In 2019, Ackermann reported a rare example of electro-oxidative nickel-catalyzed C(sp^2^)–H alkoxylation reaction with secondary alcohols (Scheme 9).^158^ This metallaelectrocatalysis exhibited high chemo- and positional-selectivity, and the plausible mechanism of this transformation involves a nickel(IV)-intermediate. Notably, various naturally occurring alcohols, such as menthol, cholesterol, and β-estradiol, were accommodated as coupling partners, delivering the corresponding aromatic ethers in excellent yields.

#### 2.1.3 Late-Stage C(sp^2^)–H Amination

**Scheme 10:**
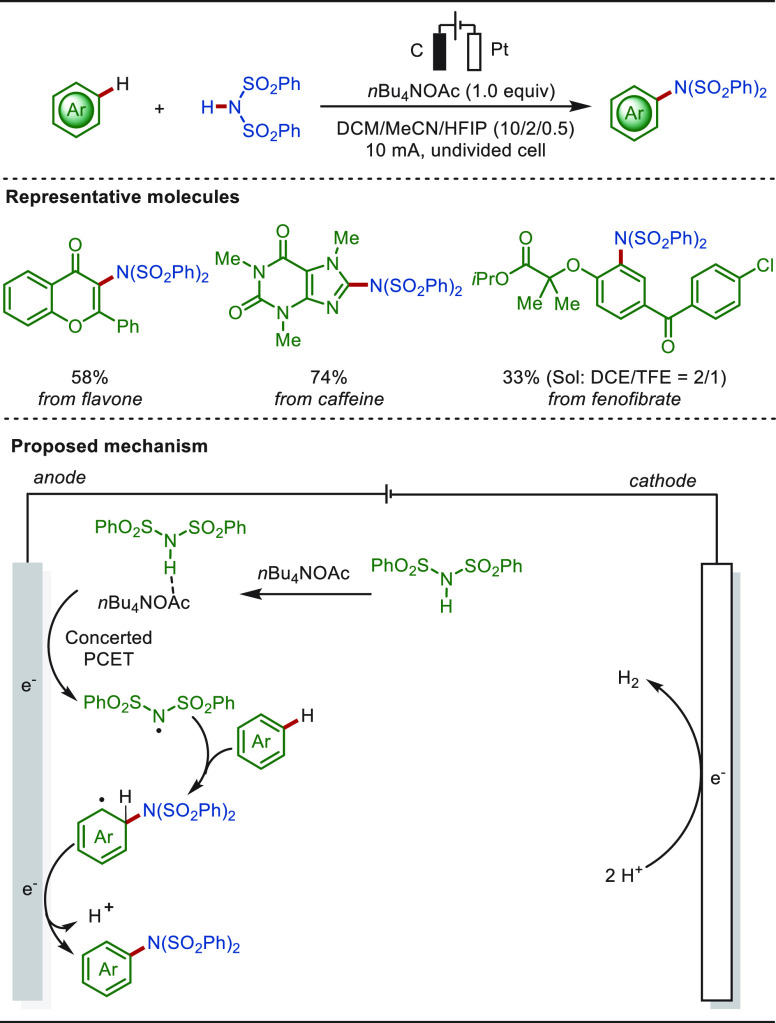
Electrochemical Late-Stage Aminations

The electro-oxidative C–H/N–H cross-coupling is a straightforward and powerful tool to install nitrogen functionalities into aromatic compounds. In 2019, the Lei group reported an intermolecular cross-coupling between sulfonimides and aromatic arenes (Scheme 10).^163^ The transformation proceeded through a nitrogen-centered radical addition pathway under transition-metal-free and exogenous oxidant-free conditions. A variety of arenes, alkenes, heteroarenes, and pharmaceuticals, such as flavone, caffeine, and fenofibrate, were amenable scaffolds. Aryl sulfonamides or aniline derivatives could thus be obtained after the deprotection process. Mechanistic studies indicated that the nitrogen-centered radicals were generated via a proton-coupled electron transfer (PCET) process jointly mediated by *n*Bu_4_NOAc and an anodic oxidation process.^163,164^ Concurrently, the Ackermann group achieved an electrochemical oxidation induced C(sp^2^)–H nitrogenation for a variety of heteroarenes, including pyrroles, indoles, benzothiophene, and benzofuran.^165^ In addition, metallaelectro-catalyzed C(sp^2^)–H aminations have been realized with diverse transition-metal catalysts (Ni, Co, Cu, etc.).^166−169^

**Scheme 11:**
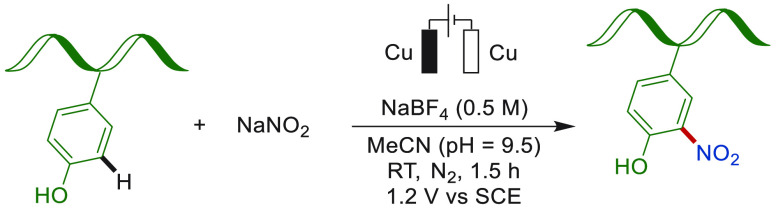
Electrochemical Late-Stage Nitrogenation

Introducing a functional group into a peptide or a protein under mild, user-friendly conditions is of great significance in the field of chemical biology, medical chemistry, and pharmacology.^170−173^ In this context, the post modification of the phenolic tyrosine side chain has become the most commonly used strategy due to its relatively high reactivity and low abundance in the proteome (2.9%). Thus, early in the 1990s, Walton and Heptinstall had reported on the modification of hen egg-white lysozyme proteins and horse heart myoglobin via electro-oxidative nitration in a mildly acidic buffer (Scheme 11).^174−178^

**Scheme 12:**
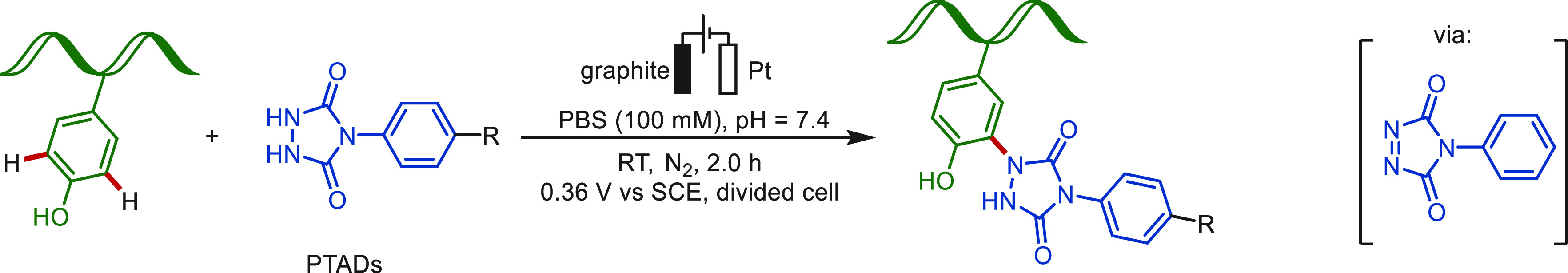
Electrochemical Peptide and Protein Modification at Tyrosine

The development of efficient methods for the conjugation of native proteins is relevant for chemical biology and biotherapies, among others. In 2010, Barbas disclosed an ene-like reaction between the tyrosine residues and substituted phenyl-3*H*-1,2,4-triazole-3,5(4*H*)-diones (PTADs).^179^ Bulk chemical oxidant and cosolvents or scavengers (e.g., Tris) needed to be employed, which limited its application scope. In 2018, Gouin and co-workers discovered the electrochemical Y-click reaction at a mild oxidative potential (+0.36 V, constant voltage electrolysis) in an aqueous buffer (Scheme 12).^73^ At the low potential conditions, the *in situ* generated reactive species is immediately conjugated with tyrosine, hence minimizing undesired side reactions and leading to a higher selectivity. The utility of this protocol was highlighted by the functionalization of a remarkably broad range of substrates. Both the small peptide hormone oxytocin and epratuzumab, a 152 kDa monoclonal antibody, were selectively modified by this method. In addition, Huan and Li recently employed this strategy to cross-link peptides and proteins at tyrosine residues without the use of photoirradiation or a metal catalyst.^180^

**Scheme 13:**
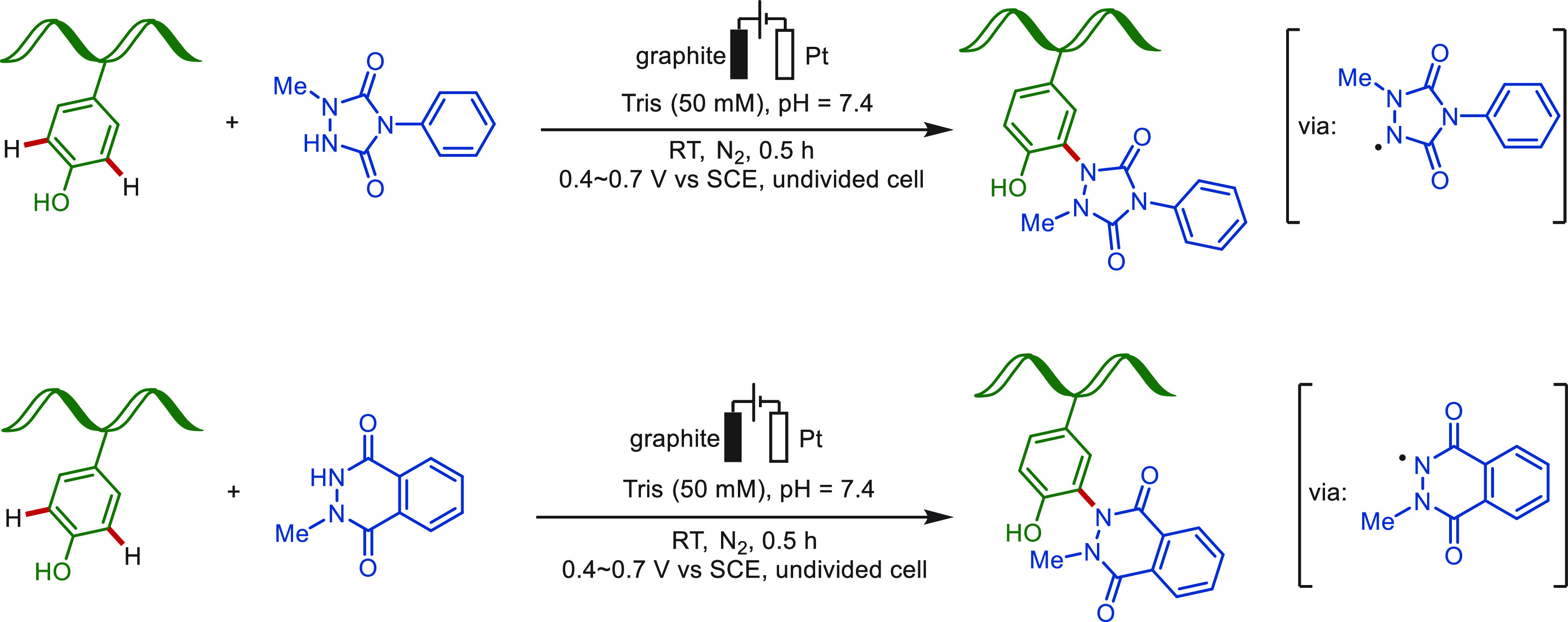
Selective Peptide Modification at Tyrosine Using *N*-Methyl Luminol and 1-Methyl-4-Phenylurazole

In 2020, Nakamura and co-workers likewise devised a modified version of the e-Y-click reaction for selective bioconjugation at tyrosine residues (Scheme 13).^181^ In their studies, *N*-methyl luminol and 1-methyl-4-phenylurazole derivatives were used as active small-molecules, which easily converted to the corresponding nitrogen radical species via the SET process under electro-oxidative conditions. A protected model octapeptide angiotensin II was successfully modified at the native tyrosine residue in a biological fashion. This approach employed purely aqueous buffer (Tris, 50 mM), neutral pH (7.4), and mild electrochemical conditions (400–700 mV), representing a truly biocompatible electrochemical modification strategies.

**Scheme 14:**
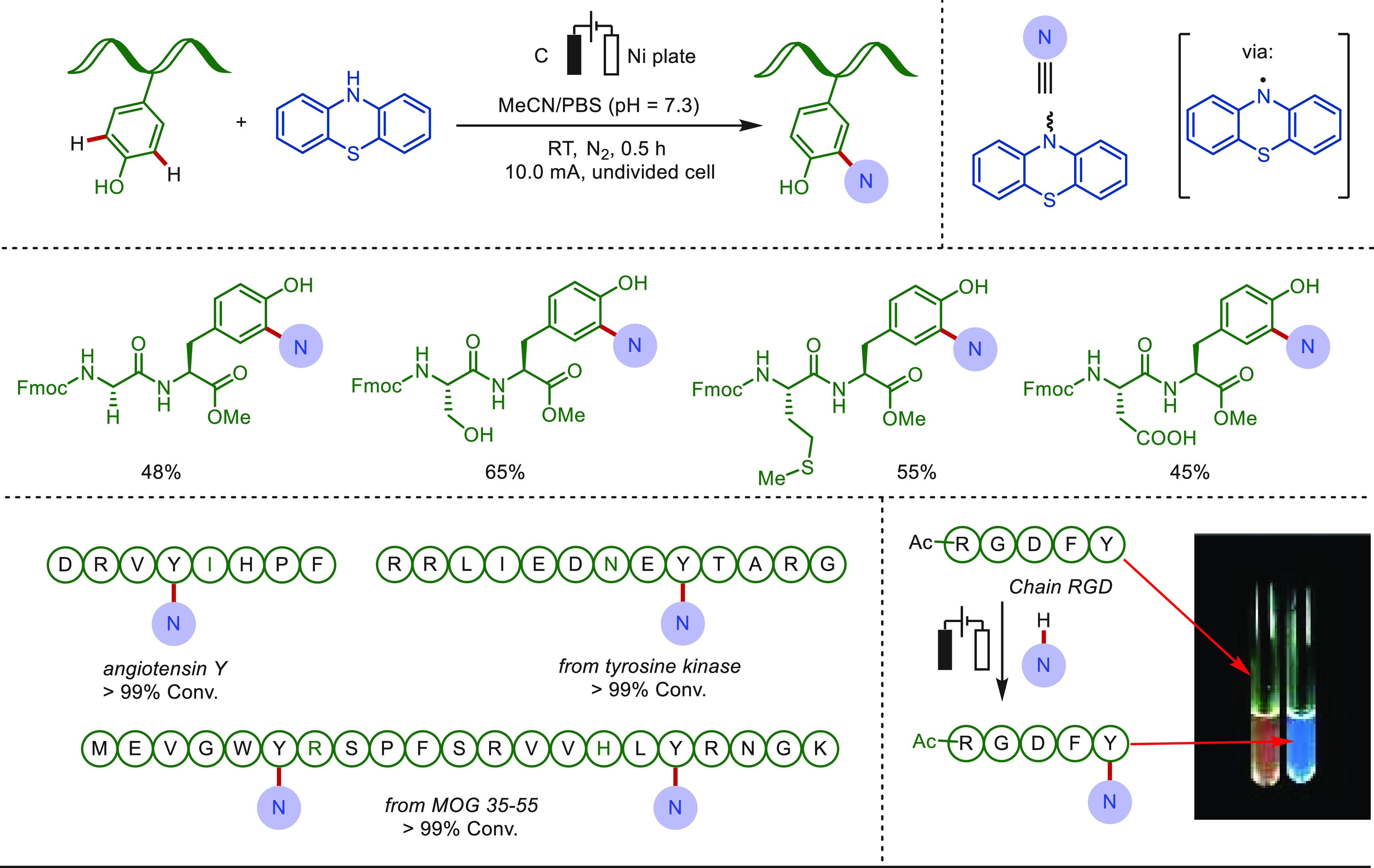
Electrochemical Late-Stage C–H Nitrogenation of Tyrosine-Containing Peptides Reproduced with permission from ref (182). Copyright 2019, Royal Society of Chemistry.

In contrast, the Lei group described an electrochemical method to execute the bioconjugation of a tyrosine side chain with phenothiazine derivatives in a simple and rapid manner (Scheme 14).^182^ This approach provided direct and efficient access to LSF of oligopeptides and proteins, featuring high chemo- and site-selectivity, without the use of transition-metal or chemical oxidants. Valuable bioactive compounds, such as angiotensin Y, tyrosine protein kinases, and MOG 35-55, selectively underwent the electro-oxidative bioconjugation process. It was also demonstrated that the phenothiazine-labeled peptide could be utilized as a fluorophore.

#### 2.1.4 Late-Stage C(sp^2^)–H Phosphorylation

**Scheme 15:**
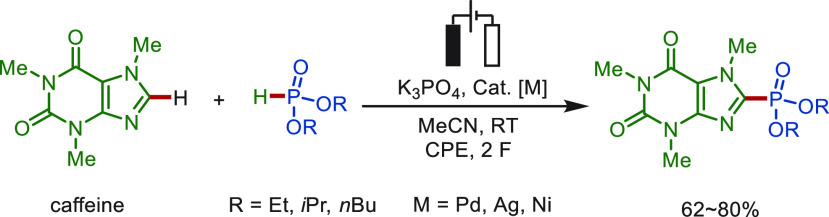
Electrocatalytic Coupling Reactions of Caffeine with Dialkylphosphites

The development of efficient late-stage phosphorylation methods is of considerable importance, given that organophosphorus compounds have wide utilities in medicinal chemistry.^183^ The past few years have seen significant development of electrochemical phosphorylation methodologies.^184−191^ In 2019, Budnikova and co-workers realized the metallaelectro-catalyzed coupling reactions of caffeine with dialkylphosphites (Scheme 15).^185^ Interestingly, diverse transition metals, including Pd(OAc)_2_, AgOAc, and Bipy_3_Ni(BF_4_)_2_ proved to be efficient for these transformations, affording the phosphorylated caffeine in good yields of 62–80%.

**Scheme 16:**
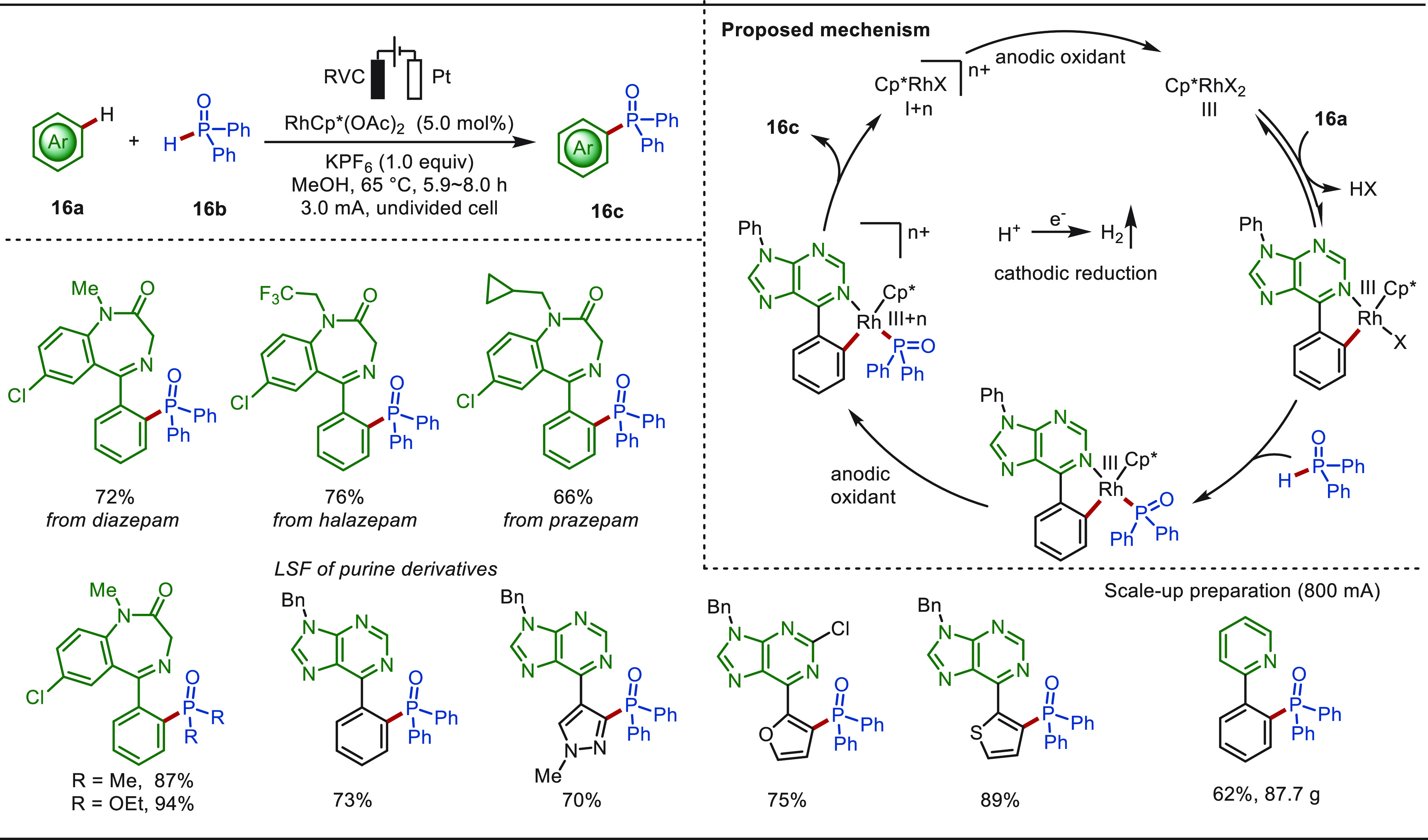
Electrochemical Rhodium-Catalyzed C(sp^2^)–H Phosphorylation

In the same year, Xu and co-workers reported an rhodaelectro-catalyzed late-stage aryl C(sp^2^)–H phosphorylation reaction with various phosphine oxides (Scheme 16).^186^ The electrochemical approach was characterized by a broad scope and high functional group tolerance without using exogenous chemical oxidants. The method proved to be compatible with the eLSF of a variety of bioactive molecules such as diazepam and purine derivatives. Notably, this electrochemical reaction was also easily scaled up, remarkably yielding a phosphonate product in 87.7 g. Mechanistic interrogation suggested that the C–P bond was formed via an oxidation-induced reductive elimination process.

**Scheme 17:**
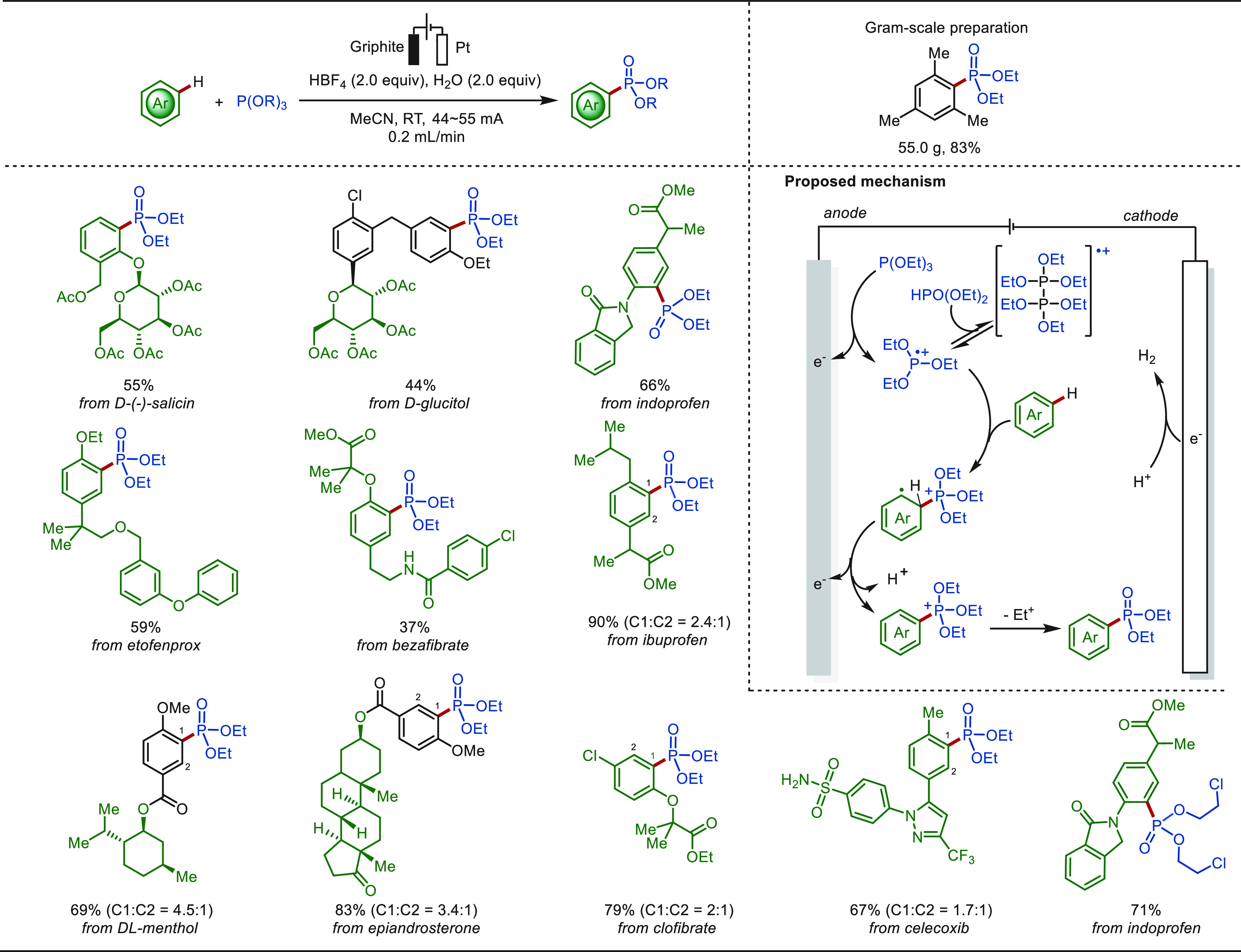
Electrochemical Late-Stage C(sp^2^)–H Phosphorylation in Continuous Flow

In 2021, the Xu group furthermore disclosed an electrochemical aromatic C(sp^2^)–H phosphorylation reaction using triethyl phosphite P(OR)_3_ in a continuous flow cell, obviating the use of exogenous chemical oxidants and transition-metal catalysts (Scheme 17).^187^ This continuous flow electrosynthesis was found to be compatible with both electron-rich and electron-deficient arenes. The practical utility of this electrochemical phosphorylation was further illustrated by the continuous production of one phosphonate product in 55.0 g. The C–P bond was formed through the reaction of arenes with *in situ* anodically generated P-radical cations. The selective late-stage functionalization of a series of bioactive compounds and natural products was accomplished through continuous flow electrosynthesis.

#### 2.1.5 Late-Stage C(sp^2^)–H Halogenation

The incorporation of a halide atom into drug molecules may have a profound effect on enhancing their biological properties.^192^ In addition, halogenated arenes, particularly aryl bromides, can be quickly converted to radio-labeled compounds, showing great utilities in metabolism studies.^193^ Moreover, halogenated arenes and heterocycles are versatile intermediates in diverse organic transformations.^194^ Therefore, the development of efficient and atom-economical halogenation methodologies under mild conditions has been a long-term goal in molecular synthesis. Traditionally, strong corrosive, oxidizing reagents (X_2_, NXS) or halides (X^−^) combined with external strong chemical oxidants were needed for halogenation of arenes.^195^ In contrast, electrochemistry offers a powerful alternative for various halogenation reactions with simple halides salts or an aqueous HX solution as the halogenating source.^196−203^

**Scheme 18:**
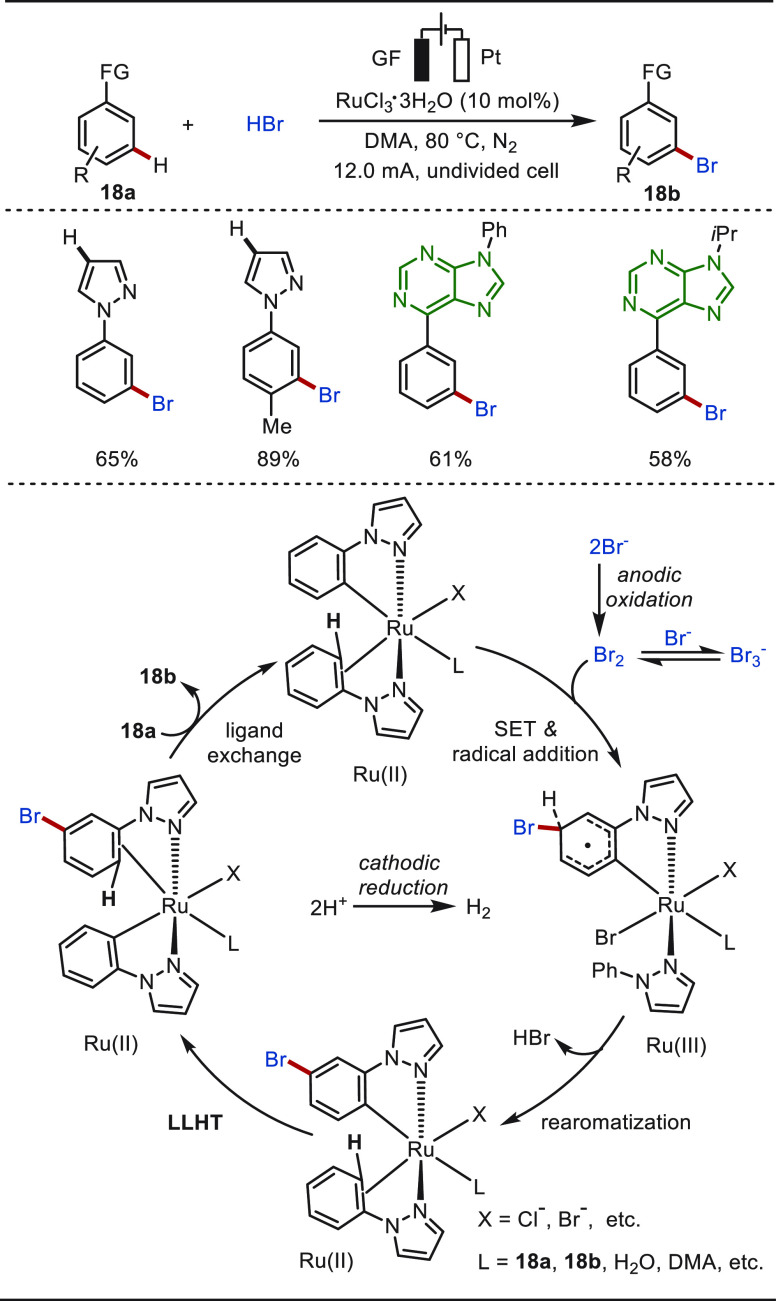
Ruthenaelectro-Catalyzed *meta*-C–H Bromination with HBr

Recently, Ackermann and co-workers disclosed electrochemical ruthenium-catalyzed distal C(sp^2^)–H bromination with an aqueous HBr solution as the brominating agent (Scheme 18).^199^ The regioselective *meta*-C–H bromination was conducted in an undivided cell by the catalysis of RuCl_3_·XH_2_O, under external ligand- and electrolyte-free conditions, featuring an ample substrate scope. Particularly, phenylpyrazole was readily brominated at the *meta*-position on the benzenoid moiety rather than at the commonly functionalized electron-rich pyrazole ring. Thus, and in sharp contrast, the bromination of pyrazolylarene under reported ruthenium/NBS conditions^204^ or electrochemical metal-free^200^ conditions proved to occur on the electron-rich pyrazole rings via a simple S_E_Ar process. Purine derivatives were identified as suitable substrates for the ruthena-electrocatalyzed *meta*-C–H bromination. Mechanistic studies revealed that the bromide ion Br^–^ was oxidized to molecular Br_2_, which equilibrated with the tribromide anion Br_3_^–^ by combining and/or releasing a bromide ion. Then the bromination process occurred between Br_2_ and the in situ generated cycloruthenated complex. The desired *meta*-brominated product was released after ligand-to-ligand hydrogen transfer (LLHT) and a ligand exchange process.

**Scheme 19:**
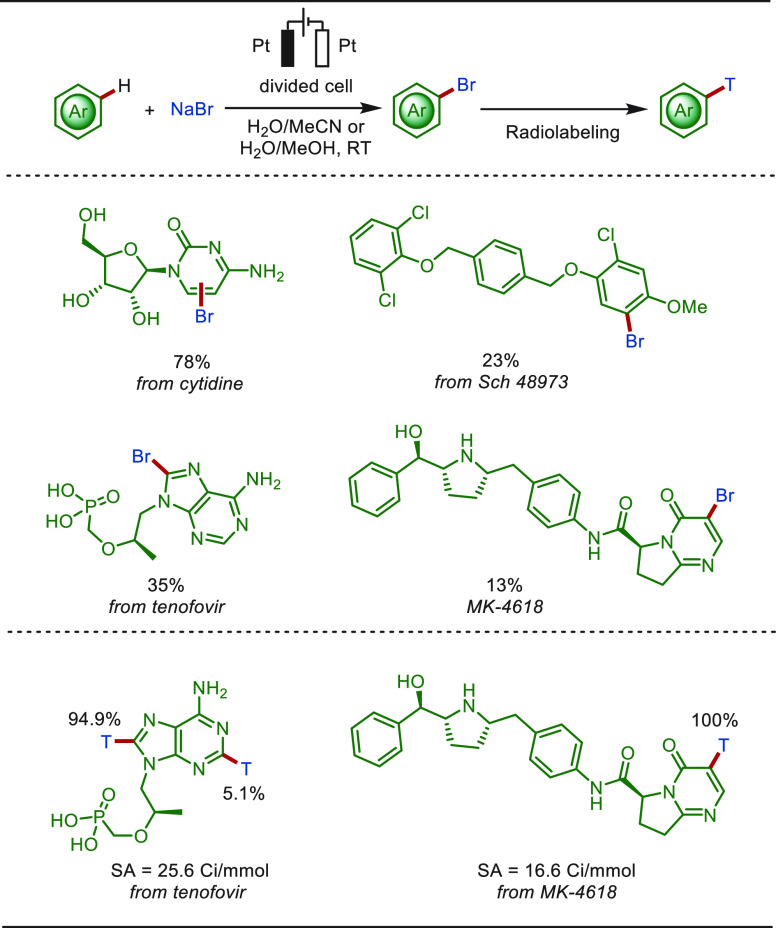
Electrochemical Late-Stage Bromination of Drug Molecules with NaBr

In 2017, Rivera and Liu disclosed an eLSF bromination method using NaBr in a mixture of water with acetonitrile/methanol (Scheme 19).^202^ The bromination reactions were conducted in a separate microflow electrochemical cell under mild conditions. Electrochemical bromination of drug molecules, including Cytidine, Sch 48793, Tenofovir, and MK-4618, gave rise to the corresponding aryl bromides. The brominated analogues of Tenofovir and MK-4618 were further converted to the corresponding tritium labeled products.

**Scheme 20:**
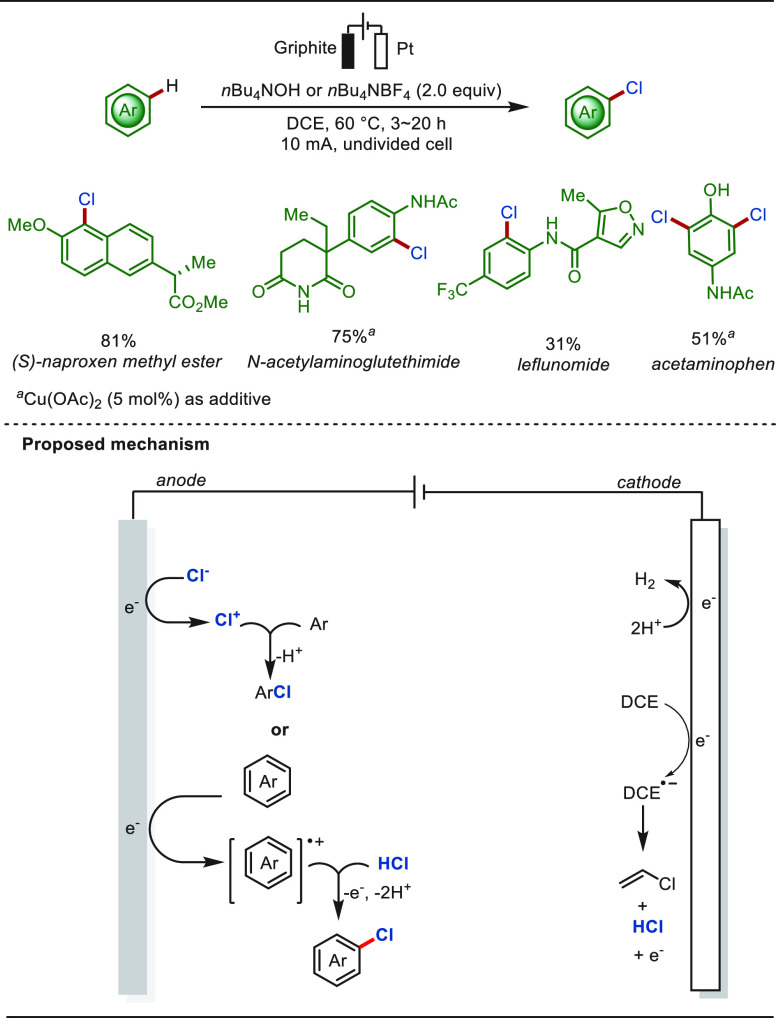
Paired Electrocatalysis for the Preparation of Aryl Chlorides Using DCE

Jiao and co-workers elegantly achieved the electrochemical aromatic chlorination with common solvent DCE as the chloro source, producing vinyl chloride as a useful byproduct (Scheme 20).^203^ In this work, the electrochemical dehydrochlorination of DCE occurred by controlling the current intensity, producing vinyl chloride and HCl. This method opened a new avenue for the preparation of (hetero)aryl chlorides and vinyl chloride in an environmentally benign manner. The mild nature and practicality of the method was further demonstrated by its easily scaled-up and efficient eLSF chlorination of a number of bioactive molecules, such as (*S*)-naproxen methyl ester, leflunomide, and acetaminophen. Using a similar strategy, McNeil and co-workers recently realized the chlorination of arenes with waste poly(vinyl chloride) as a chloro source.^83^

**Scheme 21:**
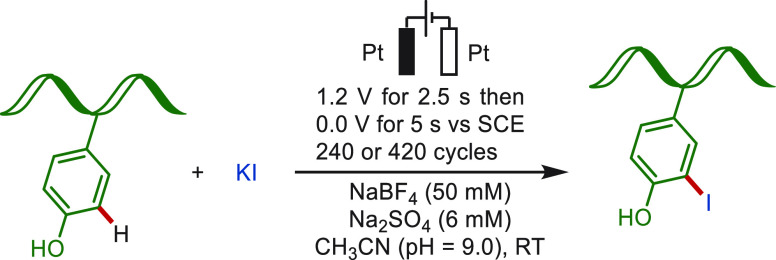
Electrochemical Late-Stage C(sp^2^)–H Iodination of Tyrosine-Containing Protein

In addition to the electrochemical protein late-stage nitrogenation (vide supra), Heptinstall and co-workers have developed the selective electrochemical iodination of horse heart myoglobin with KI (Scheme 21).^205^ Since rapid anodic oxidation of an iodide anion led to persistent formation of the undesirable triiodide, the authors used an innovative “redox pulse” method (2.5 s at 0.4 V vs SCE and 5 s at 0.0 V vs SCE, 240 cycles) to enable mono- and double iodination of myoglobin with high levels of selectivity. Notably, this exquisitely controlled protein iodination strategy could proceed at both high and very low iodide concentrations, offering improved selectivity compared to those of reported chemical and enzymatic methods.

#### 2.1.6 Late-Stage Annulation Reactions via C(sp^2^)–H Activations

In the past two decades, annulations via C–H bonds activation have revolutionized the art of preparing cyclic compounds.^206−208^ Particularly, the electrochemical annulations have gained significant recent momentum without the use of sacrificial chemical oxidants, such as Cu(OAc)_2_ and AgOAc, avoiding the generation of undesired byproducts and increasing the atom economy.^209−220^ Diverse five-, six-, and seven-membered rings have been efficiently assembled through formal [3 + 2], [4 + 1], [4 + 2], or [5 + 2] cycloadditions. However, only a small part of these tools were exploited for chemo-selective eLSF.

**Scheme 22:**
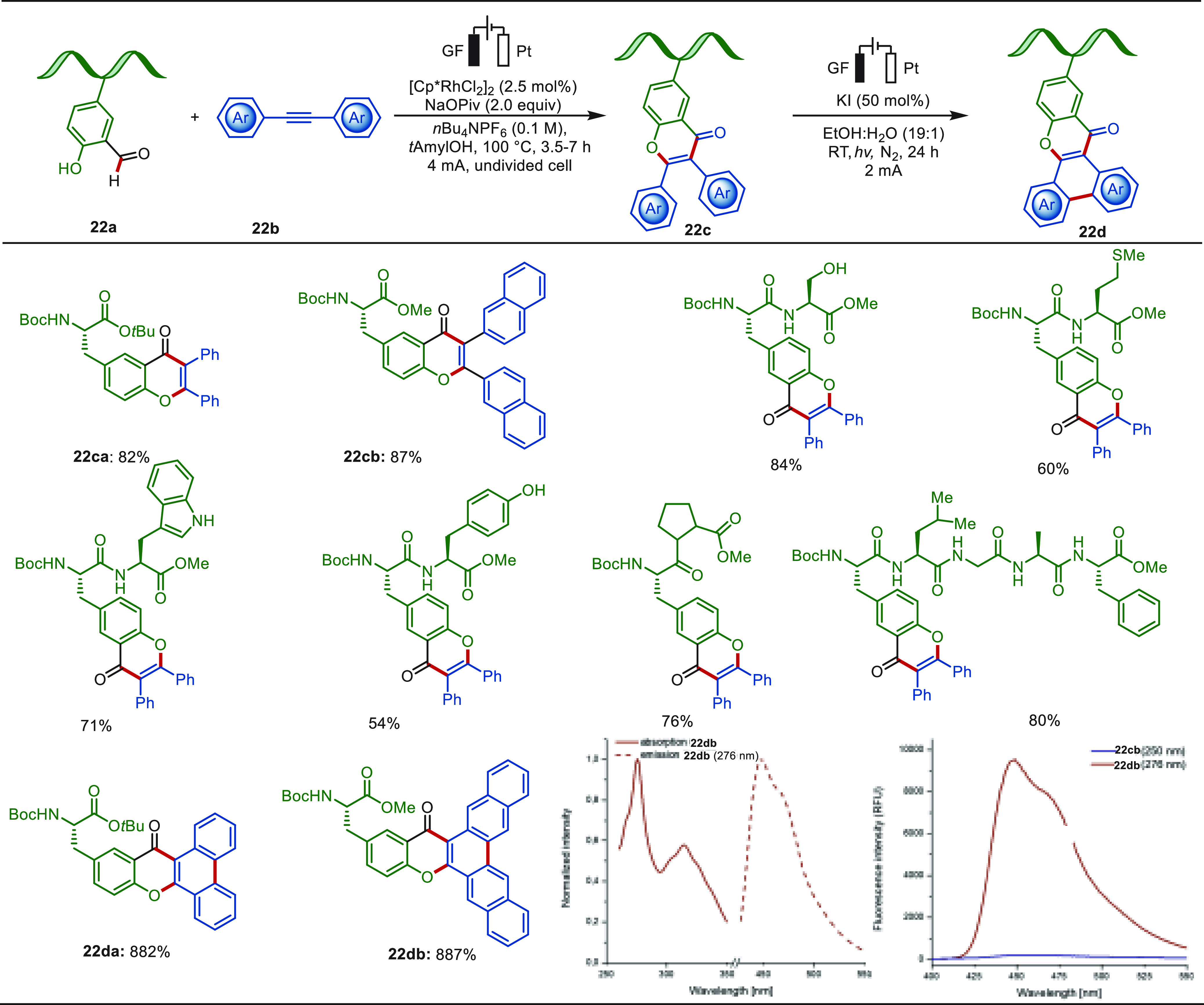
Rhodaelectro-Catalyzed Peptide Late-Stage Labeling via Formyl C–H Activation Reproduced with permission from ref (221). Copyright 2021, Springer Nature.

In 2021, the Ackermann group reported on the rhodaelectro-catalyzed annulations of 2-hydroxybenzaldehydes with alkynes by electrochemical formyl C–H activation (Scheme 22).^221^ The strategy was applicable to the functionalization of tyrosine derivatives and hence enabled access to site-selective electrolabeling of tyrosine-derived fluorescent amino acids and peptides. A broad variety of dipeptides, even including oxidation-sensitive methionine and serine containing peptides as well as polypeptide, were efficiently converted to the desired products. Mechanistic studies provided strong support for an oxidation-induced reductive elimination within a rhodium(III/IV/II) manifold. Notably, a mediated photoelectrochemical oxidation of the modified amino acids allowed for access to π-extended peptide labels, which exhibited intense fluorescence and have great potential as fluorogenic probes.

**Scheme 23:**
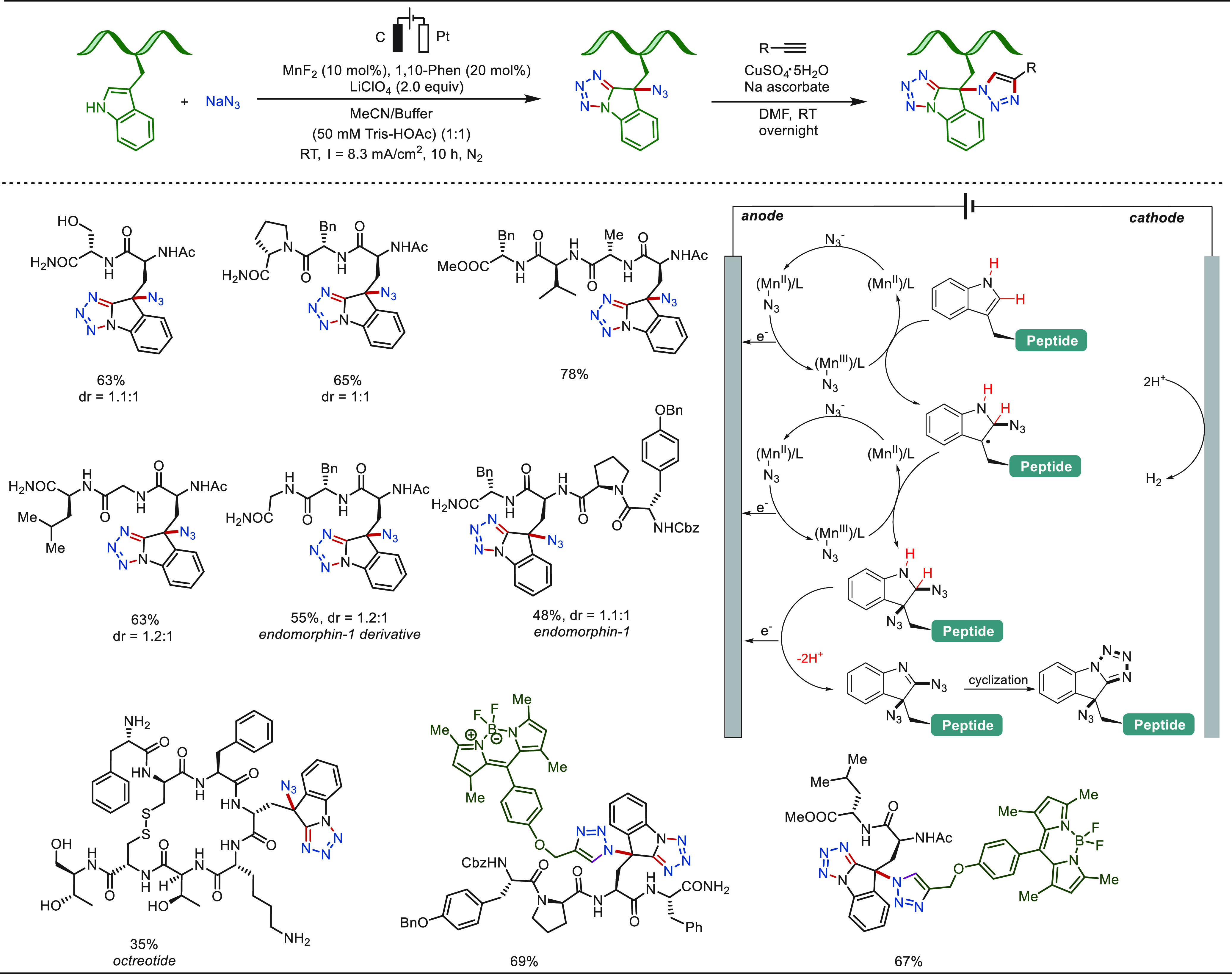
Tandem Electrochemical Oxidative Azidation/Heterocyclization of Tryptophan-Containing Peptides

Very recently, Weng and co-workers developed an electrochemical LSF of tryptophan-containing peptides with NaN_3_ to afford azide-substituted tetrazolo[1,5-α]indolecontaining peptides (Scheme 23).^222^ This reaction used an earth abundant Mn catalyst under mild buffered conditions. This strategy was applicable for a wide range of peptides with good functional-group tolerance and high site-selectivity. In addition, the thus-obtained Trp-containing peptides with an azide group could be further derivatized to various triazole products by a copper-catalyzed “click” reaction. The reaction was proposed to proceed through manganese-mediated diazidation of the indole unit, followed by the dehydrogenation and heterocyclization to deliver the LSF products.

**Scheme 24:**
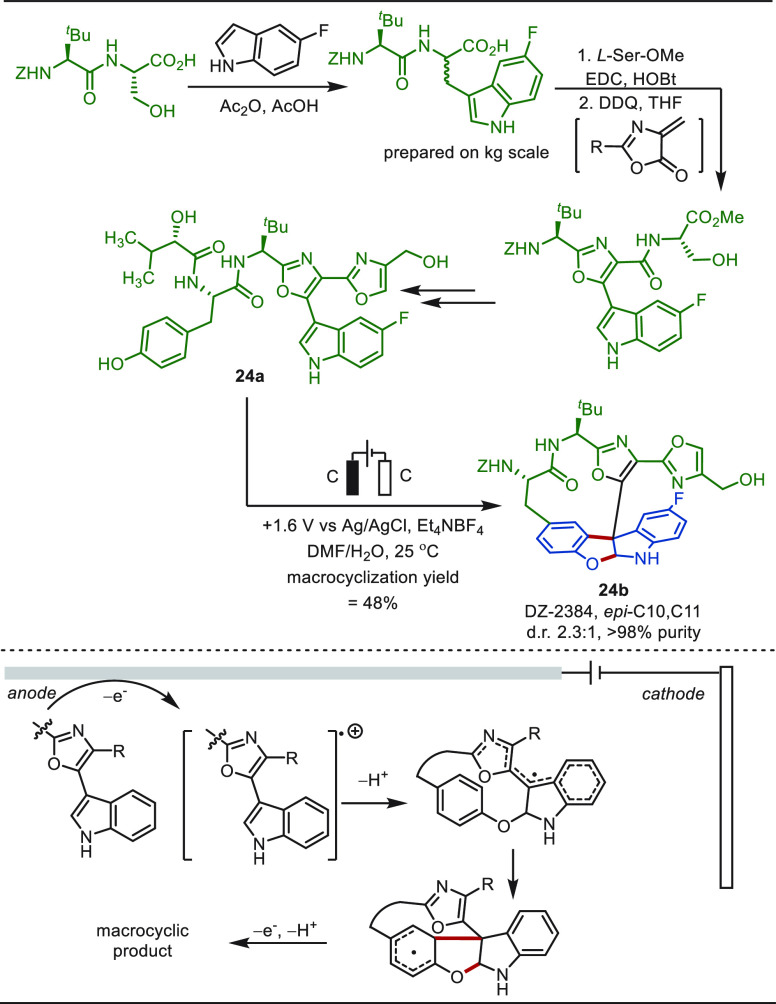
Electrochemical Oxidative Macrocyclization for the Synthesis of DZ-2384

The synthesis of macrocycles—abundant motifs in biologically relevant molecules and pharmaceuticals—continues to represent a popular arena for synthesis chemists.^223−227^ However, the construction of large ring systems is challenging, considering geometrical and thermodynamic constraints. Recently, electrochemical transformations have emerged as useful techniques in this regard. In 2015, Harran uncovered an electro-oxidative late-stage macrocyclization strategy for a scalable synthesis of antimitotic agent DZ-2384 (Scheme 24).^228^ The synthetic strategy used the dipeptide *tert*-Leu-5-F-Trp-OH as the precursor for the synthesis. After strategic modification over this dipeptide, advanced intermediate **24a** was synthesized, which upon constant potential electrolysis realized an oxidative cyclization on the indole core to construct the DZ-2384 analogue in moderate yields. The oxidative cyclization involved SET oxidation of **24a**, which then underwent a nucleophilic attack with the phenolic −OH functionality present in the molecule. Next a *5*-*exo*-*trig* cyclization with the arene counterpart followed by aromatization generated the DZ-2384.

### 2.2 eLSF of C(sp^3^)–H Bonds

#### 2.2.1 Late-Stage Benzylic C(sp^3^)–H Functionalization

**Scheme 25:**
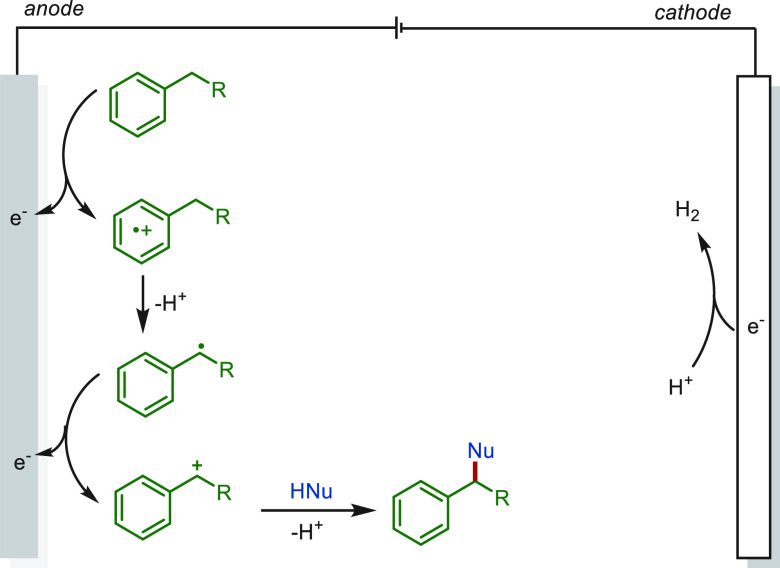
Proposed Mechanism for Electrochemical Benzylic C(sp^3^)–H Functionalization

Benzylic C(sp^3^)–H bonds are ubiquitous in natural products and drug molecules. About 25% of the 200 best-selling drugs contain benzylic C–H bonds. The relatively low bond dissociation energy of benzylic C–H bonds enables high site-selectivity among various types of C–H bonds in structurally complex molecules.^229^ Therefore, benzylic C(sp^3^)–H functionalization has wide application foreground in the LSF field and has received a great deal of attention. In this context, significant progress was achieved for electrochemical benzylic C(sp^3^)–H functionalization.^230^ The reaction mechanism was proposed as following for most cases: Anodic oxidation of the hydrocarbon substrate gives an arene-centered radical cation, which undergoes rapid proton transfer and a second electron transfer oxidation to form a benzylic cation. Then, the intermediate was trapped by a nucleophilic reagent to afford the final product (Scheme 25). Meanwhile, hydrogen gas is generated at the cathode. Notably, the selection of a suitable solvent generally plays a key role in such transformation to modulate the oxidation potentials of the starting substrate and the product to avoid overoxidation.

**Scheme 26:**
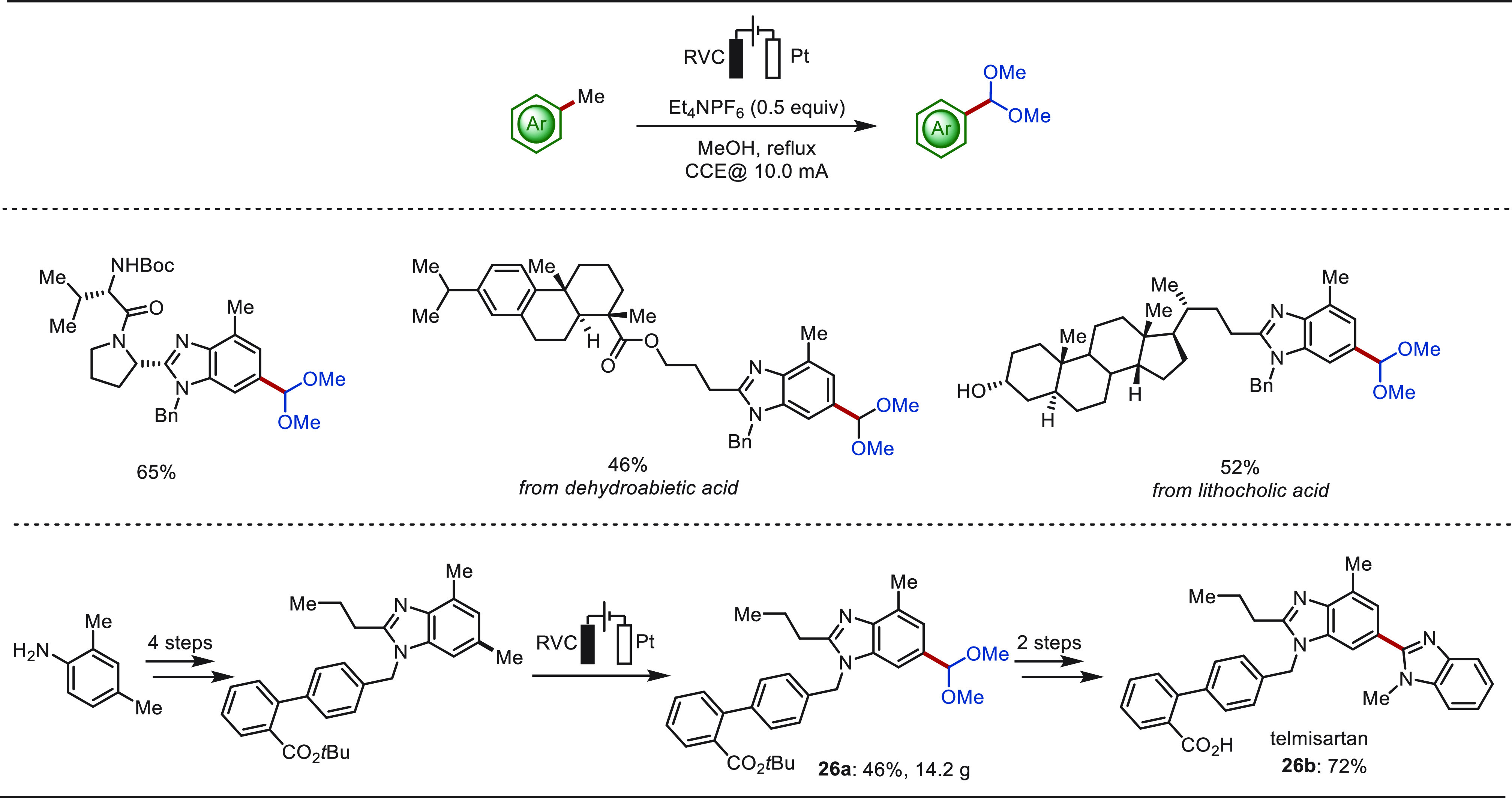
Electrochemical Late-Stage Oxidation of Various Methylarenes

The formyl group is a synthetically versatile functional group that can be converted to a variety of functionalities. The oxygenation of methylarenes to benzaldehyde derivatives is of significant practical interest for LSF as the benzyl methyl motifs are widely present in drug molecules. However, control of chemo-selective oxidation with highly functionalized methylarenes remains a significant challenge due to the product overoxidation and selectivity issues for substrates featuring multiple oxidizable C–H bonds.^231,232^ Recently, the Xu group disclosed an electrochemical method that can site-selectively oxidize methyl benzoheterocycles to aromatic acetals in an undivided cell setup, without the utility of transition-metal catalysts and exogenous chemical oxidants (Scheme 26).^233^ The acetals could be easily hydrolyzed to the corresponding aldehydes in one-pot or in a separate step. This electro-oxidation approach was amenable to various functionalized benzoheterocycles and medicinally relevant molecules. The utility of this electro-oxidation reaction was further demonstrated by the efficient construction of the antihypertensive drug telmisartan **26b**, in which the key dimethyl acetal intermediate **26a** was obtained on a 14.2 g scale by site-selective electro-oxidation reaction.

**Scheme 27:**
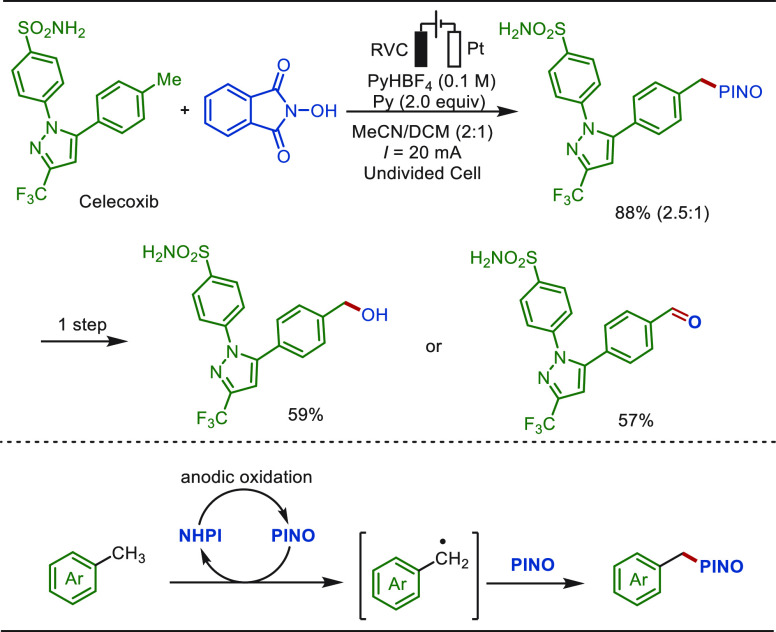
NHPI Mediated Late-Stage Benzylic Oxidation of Methylarenes

The oxidation of methylarenes is generally ineffective for electron-neutral and electron-deficient arenes since their higher redox potentials lead to poor selectivity or competitive solvent oxidation. A NHPI (*N*-hydroxypthalimide) mediated electrosynthetic method was developed by Stahl and co-workers to overcome these limitations (Scheme 27).^234^ In their studies, proton-coupled electrochemical oxidation of NHPI generated the PINO (phthalimide-N-oxyl) radical, which serves as a hydrogen-atom-transfer (HAT) mediator and as a metastable persistent radical to trap the *in situ* generated benzylic radicals. This PINOylation reaction operated at ∼0.5–1.5 V lower electrode potentials compared with the direct electrolysis methods, and hence enables the mediated electrolysis approach to tolerate a broad scope of methylarenes with diverse electronic properties and ancillary functional groups. The synthetic utility of this method was clearly reflected by facial conversion of the thus-obtained products into benzylic alcohols or aldehydes under photochemical conditions, both of which are compatible with LSF of the nonsteroidal anti-inflammatory pharmaceutical celecoxib.

**Scheme 28:**
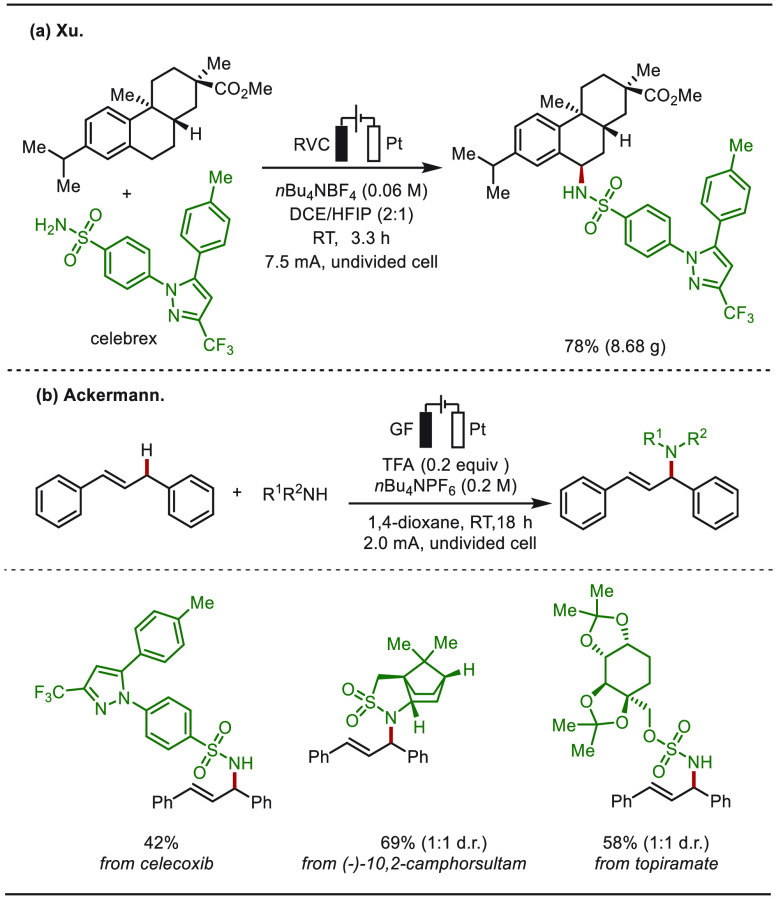
eLSF by Benzylic C–H Amination

In 2021, Xu and co-workers reported on a site-selective electrochemical benzylic C–H amination via the hydrogen evolution reaction (HER) without the need of exogenous oxidants or transition-metal catalysts (Scheme 28a).^235^ The practical utility of this electrochemical C–H amination reaction was illustrated by a gram-scale preparation with Celebrex as the aminating source, giving the corresponding C(sp^3^)–N coupling product in 78% yield. Meanwhile, the Ackermann group disclosed an effective method for electrochemical C–H aminations of 1,3-diarylpropenes via direct oxidative C(sp^3^)–H functionalizations with various substituted amides including the chiral auxiliary (−)-10,2-camphorsultam as well as the sulfonamide drugs Celebrex and Topiramate (Scheme 28b).^236^

**Scheme 29:**
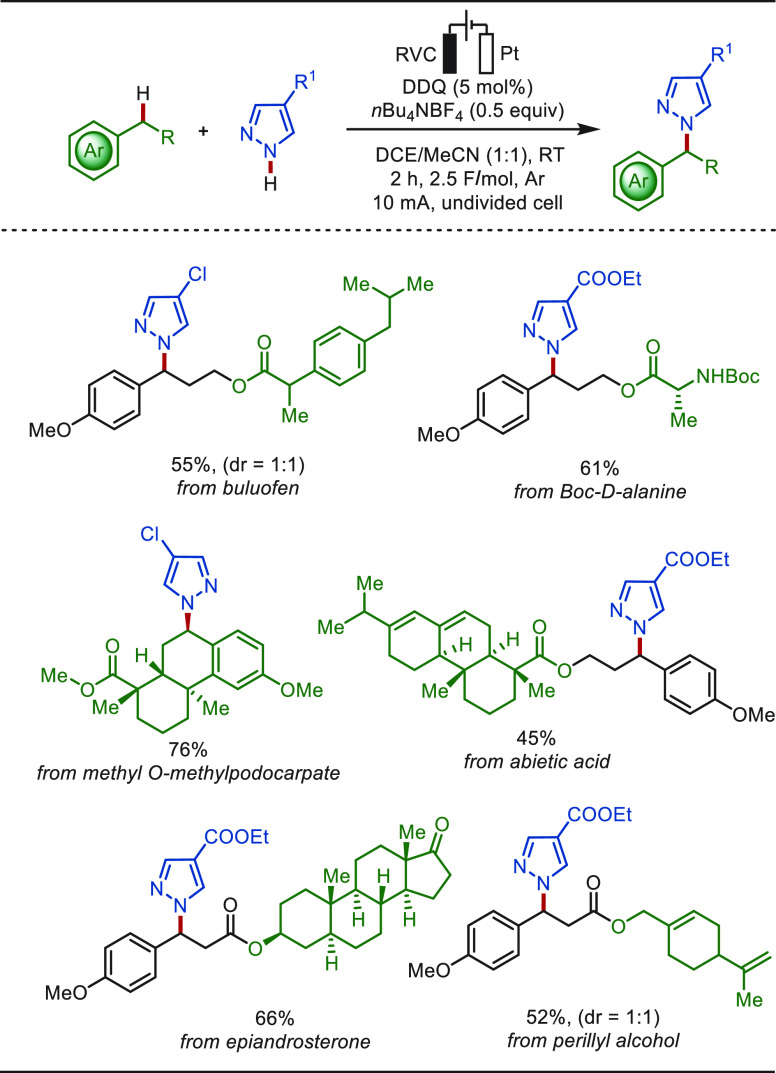
DDQ-Mediated Late-Stage Benzylic C–H Azolation

The Wang group also achieved a similar benzylic C–H amination reaction with diverse pyrazoles in a mixture of DCE and MeCN as solvents (Scheme 29).^237^ DDQ (2,3-dichloro-5,6-dicyano-1,4-benzoquinone) was employed as a redox mediator to improve the electrolysis efficiency. The compatibility of this electrochemical strategy was demonstrated by the late-stage aminations of bioactive molecule substrates deriving from buluofen, abietic acid, epiandrosterone, and perillyl alcohol.

**Scheme 30:**
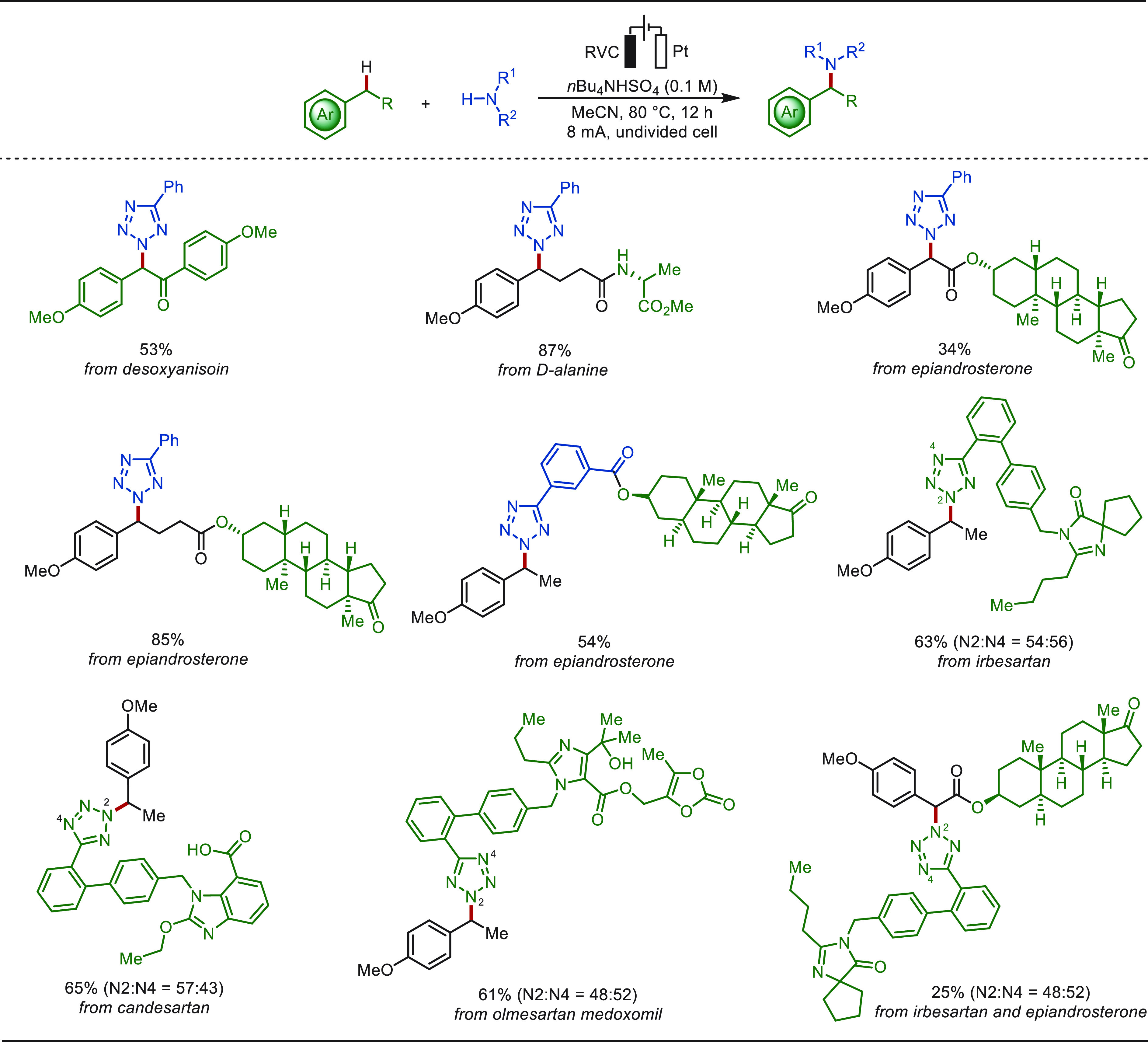
Electrochemical Late-Stage Benzylic C–H Azolation

Later, Ruan and co-workers showed that azoles were suitable amination reagents for the electrochemical C–H/N–H cross-coupling reactions, with *n*Bu_4_NHSO_4_ as the electrolyte and MeCN as the solvent in an undivided cell (Scheme 30).^238^ The azolation occurred efficiently and selectively at primary, secondary, and even challenging tertiary benzylic positions. This approach was directly exploited to install azole or benzyl motifs on a variety of structurally complex drug molecules.

**Scheme 31:**
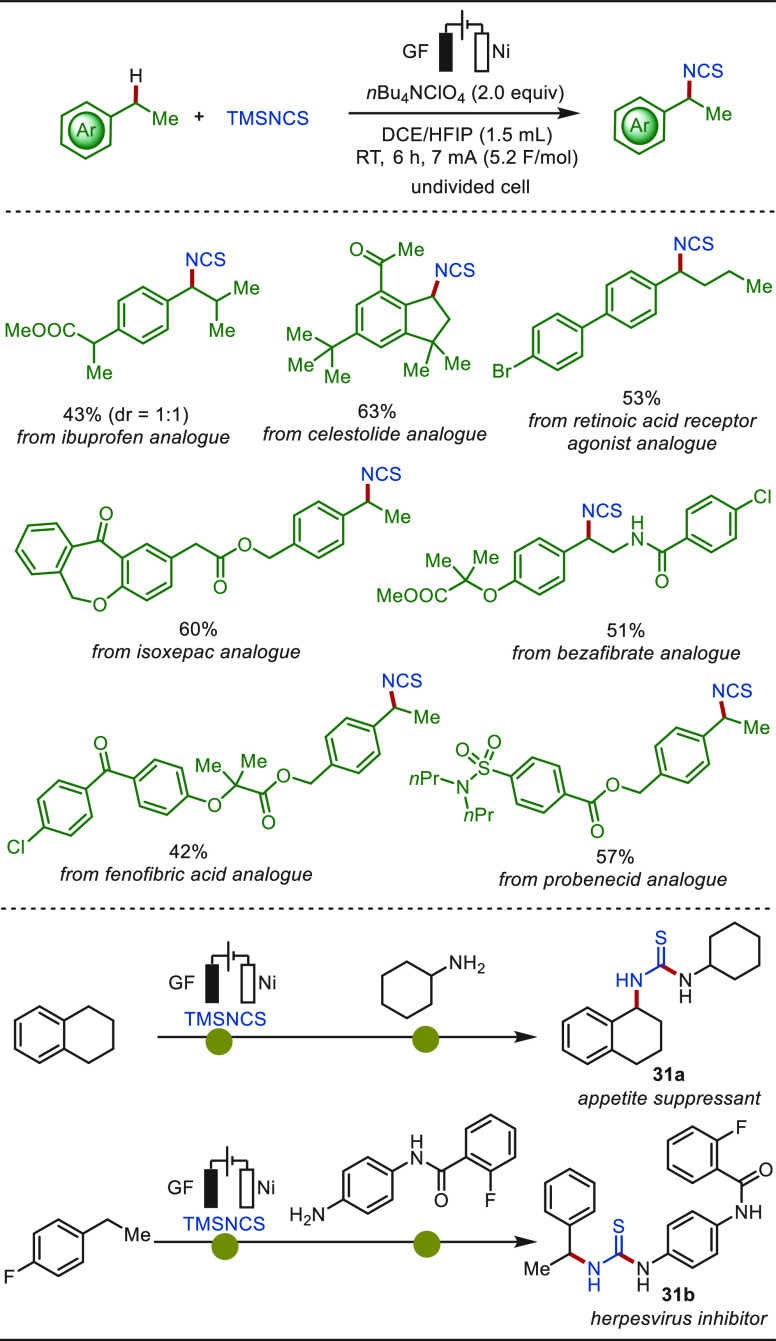
Electrochemical Late-Stage Benzylic C–H Isothiocyanation

Direct C(sp^3^)–H isothiocyanations represent a straightforward strategy for the introduction of the versatile isothiocyanate functional group.^239^ Recently, Guo and Wen disclosed an electrochemical late stage benzylic C(sp^3^)–H isothiocyanation with TMSNCS (Scheme 31).^240^ A broad range of drug and bioactive molecules smoothly underwent the isothiocyanation under mild conditions with high chemo- and regio-selectivity. The chemoselectivity was attributed to the ready isomerization of in situ generated thiocyanates to isothiocyanates under the electrolysis conditions. In addition, with the electrochemical isothiocyanation strategy, two drug molecules—appetite suppressant **31a** and herpesvirus inhibitor **31b**—were prepared in a one-pot, two-step procedure from readily available alkylated arenes. In contrast, previous syntheses of these two compounds required four- and three-step processes, respectively.

**Scheme 32:**
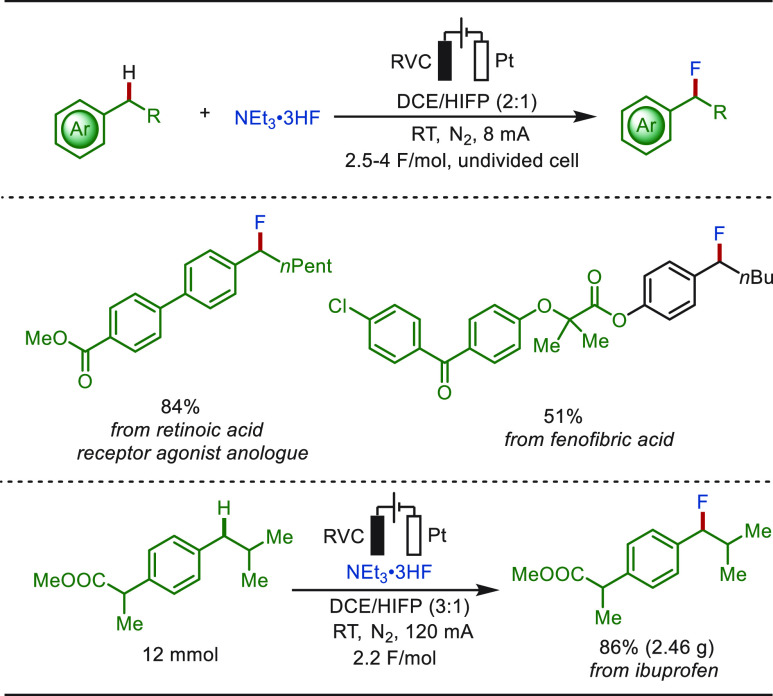
Electrochemical Late-Stage Benzylic C–H Fluorination

The straightforward and efficient introduction of fluorine is of great important in medicinal chemistry because of the unique properties of the C–F bond.^241−245^ In recent years, electrochemical fluorinations of C(sp^3^)–H bonds with nucleophilic fluoride sources have gained more attention.^246−249^ Recently, the Ackermann group developed a selective electrochemical C(sp^3^)–H fluorination with readily available NEt_3_·3HF, in lieu of alternative expensive electrophilic fluorine reagents (Scheme 32).^249^ External oxidants and transition-metal catalysts, as well as directing groups, were not required. The method displayed broad functional group tolerance, setting the stage for the late-stage fluorination of bioactive drugs. The practical utility was substantiated by fluorination of ibuprofen on a large scale of 2.5 g. Notably, adamantane was fluorinated at the tertiary position under otherwise identical electrolysis conditions, implying considerable potential for alkane modification. In addition, the synthetic utility of the C(sp^3^)–H fluorination could be further illustrated by a subsequent one-pot arylation of the generated benzylic fluorides.

**Scheme 33:**
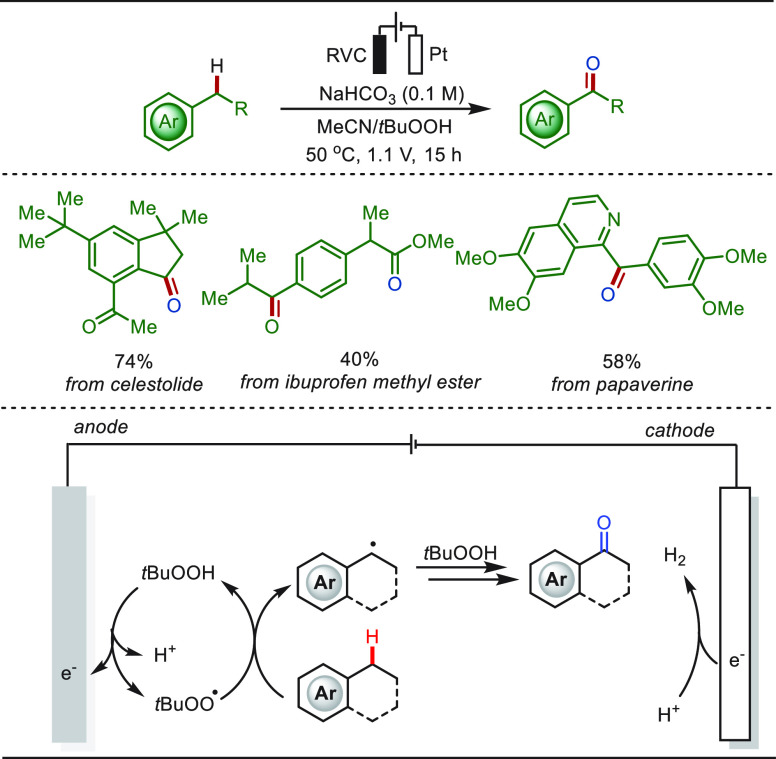
Electrochemical Late-Stage Benzylic C–H Bonds Oxidation to Form Ketones

Ketones are versatile functional groups and omnipresent in natural products and biologically active compounds.^250^ Recently, Liu and co-workers developed a sustainable protocol for direct benzylic C–H bond oxidation of alkylarenes to provide the corresponding ketone compounds with *tert*-butyl hydroperoxide as the radical- and oxygen-source (Scheme 33).^251^ The *tert*-butyl peroxyl radical was first generated by mild anodic oxidation. Then, the hydrogen atom transfer (HAT) occurred to form a benzylic radical, which reacts with *t*BuOOH, affording the corresponding ketone. This approach was successfully applied to the LSF of bioactive molecules, including celestolide, ibuprofen methyl ester, and papaverine, in synthetically useful yields without affecting other functional groups.

#### 2.2.2 Late-Stage Allylic C(sp^3^)–H Functionalization

Over the past decade, late-stage allylic C(sp^3^)–H functionalization has attracted substantial interest among organic chemists, benefiting from the fact that the carbon–carbon double bond is widely present in natural products and drug molecules. The allylic C(sp^3^)–H bond features relatively low bond dissociation energy, which enables high site selectivity in structurally complex molecules. In this context, electrochemical late-stage allylic C(sp^3^)–H functionalization has witnessed considerable recent progress.^252−255^

**Scheme 34:**
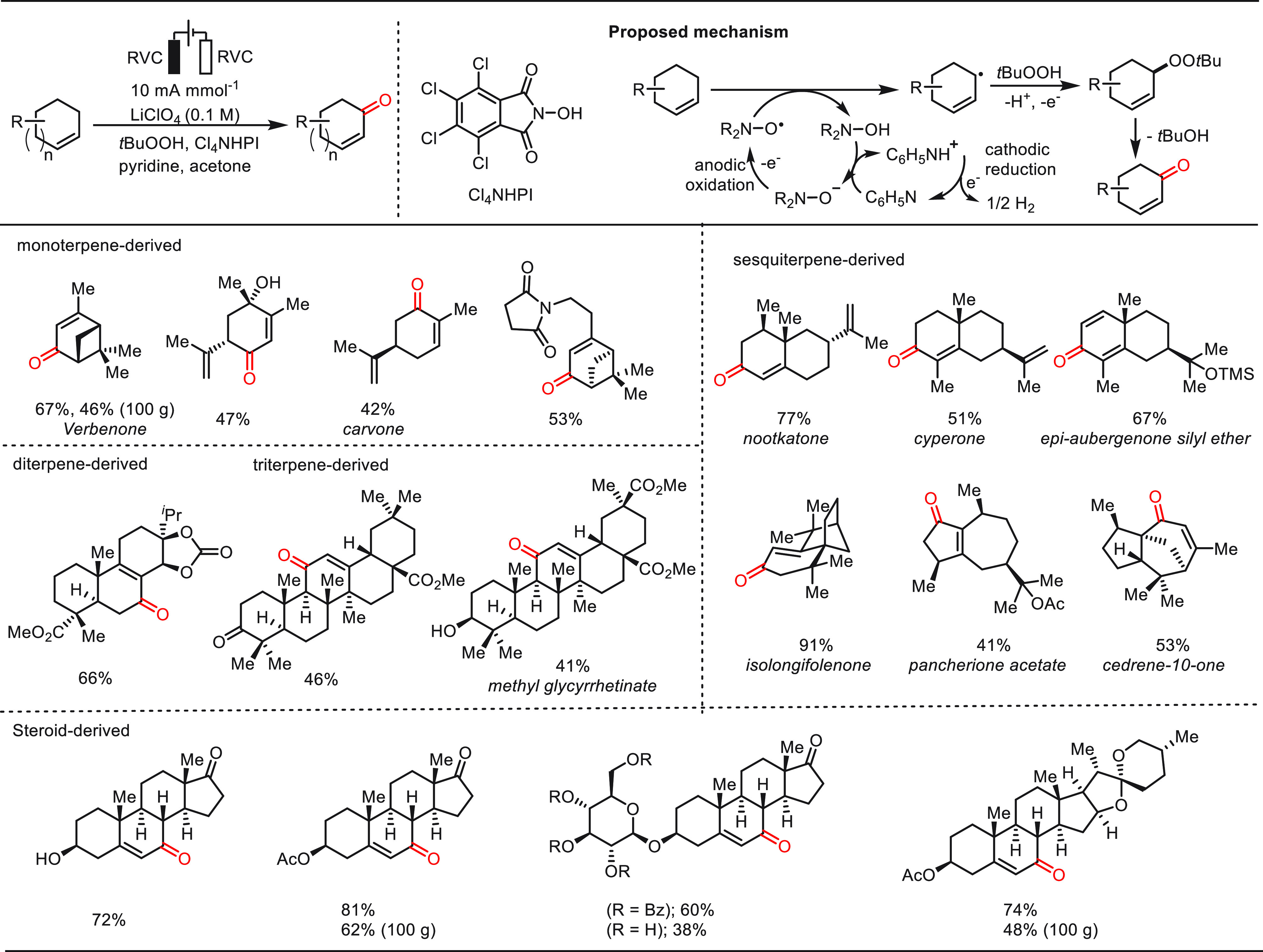
Electrochemical Late-Stage Allylic C(sp^3^)–H Oxidation

In 2016, Baran and co-workers described an elegant electrochemical allylic C(sp^3^)–H oxidation strategy using 20 mol % Cl_4_NHPI as a redox mediator, pyridine (2.0 equiv) as the base, *t*BuOOH (1.5 equiv) as a co-oxidant, and LiClO_4_ as the electrolyte (0.1 M) in acetone under constant-current conditions in an undivided cell (Scheme 34).^255^ This powerful electrochemical approach was characterized by a broad substrate scope, high chemoselectivity, and operational simplicity. A variety of representative terpenes were oxidized under the electrolysis conditions, affording corresponding versatile monoterpenes, sesquiterpenes, diterpenes, triterpenes, and steroids, which have outstanding utilities in food, fragrance, and pharmaceuticals industries. Notably, the user-friendly and robust nature of this electrochemical allylic C–H oxidation was demonstrated by 100 g preparation of several products with good efficiency.

**Scheme 35:**
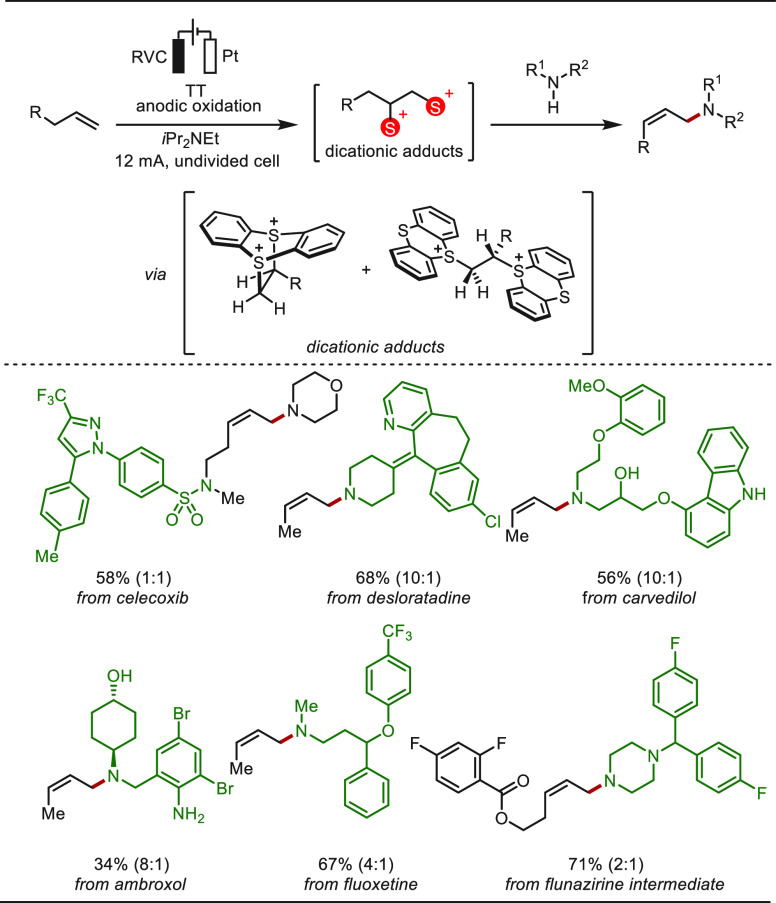
Electrochemical Late-Stage Allylic C(sp^3^)–H Amination

Allylic amines are valuable building blocks in molecular synthesis and they are likewise prevalent in diverse biologically active molecules.^256−258^ In 2021, Wickens and co-workers developed a most user-friendly electrochemical strategy to prepare aliphatic allylic amines by the oxidative coupling of unactivated alkenes with secondary aliphatic amines (Scheme 35).^259^ This reaction proceeded via the electrochemical formation of a dicationic alkene-bis(thianthrene) adduct between thianthrene (TT) and the alkene substrate. Treatment of these adducts with aliphatic amines and base efficiently provides the corresponding linear, tertiary allylic amine products in high *Z* selectivity. Complex biologically active molecules are amenable to this transformation as both amine and alkene partners. Mechanistic studies revealed the vinylthianthrenium salts as the key reactive intermediates.

#### 2.2.3 Late-Stage α-C(sp^3^)–H Functionalization of Carbonyls

The diversification of the C(sp^3^)–H bond adjacent to a carbonyl group is among the most basic transformations of utmost utility in molecular chemistry. Representative examples include the Claisen condensation, aldol reactions, or the Mannich reaction. In this context, α-C(sp^3^)–H functionalization of carbonyl compounds has been extensively explored.^260−268^ Particularly, significant recent momentum has been gained in eLSF and preparation of pharmaceutical derivatives.

**Scheme 36:**
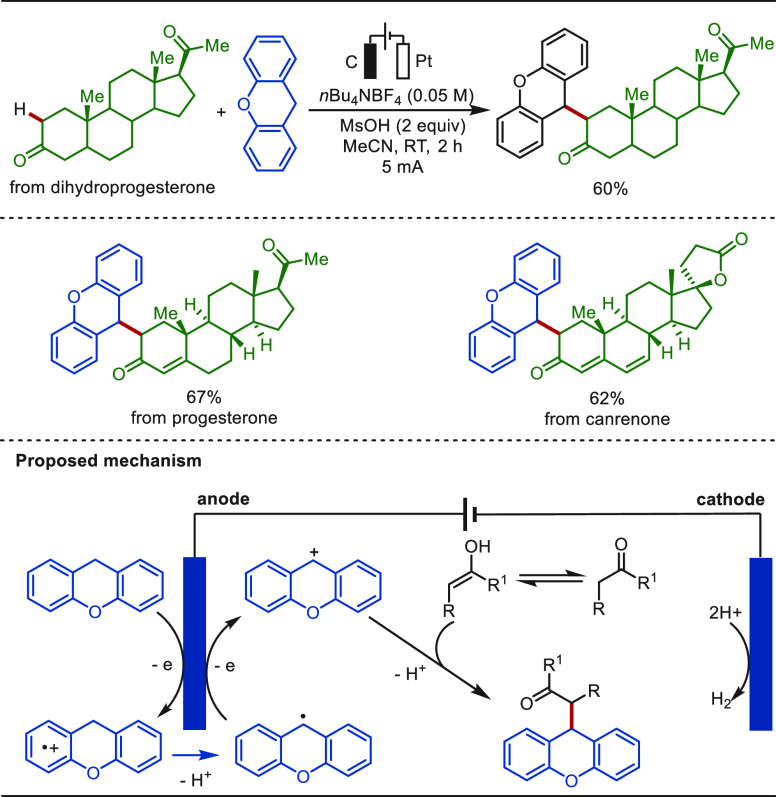
Electrochemical Dehydrogenative Cross-Coupling of Ketones with Xanthenes (66)

In 2020, Li and Song reported a practical electro-oxidative dehydrogenative cross-coupling of ketones with xanthenes (Scheme 36).^261^ This transformation was performed under mild conditions, featuring a high atom economy and excellent functional-group tolerance. Drug molecules including dihydroprogesterone, progesterone, and canrenone proved to be compatible with the electrochemical C(sp^3^)–H/C(sp^3^)–H cross-coupling reactions, giving the corresponding products in excellent yields. Mechanistic studies indicated that a stabilized carbocation was first generated via anodic oxidation of xanthene. Then, the intermediate reacted with the nucleophilic enol to afford the cross-coupling product.

**Scheme 37:**
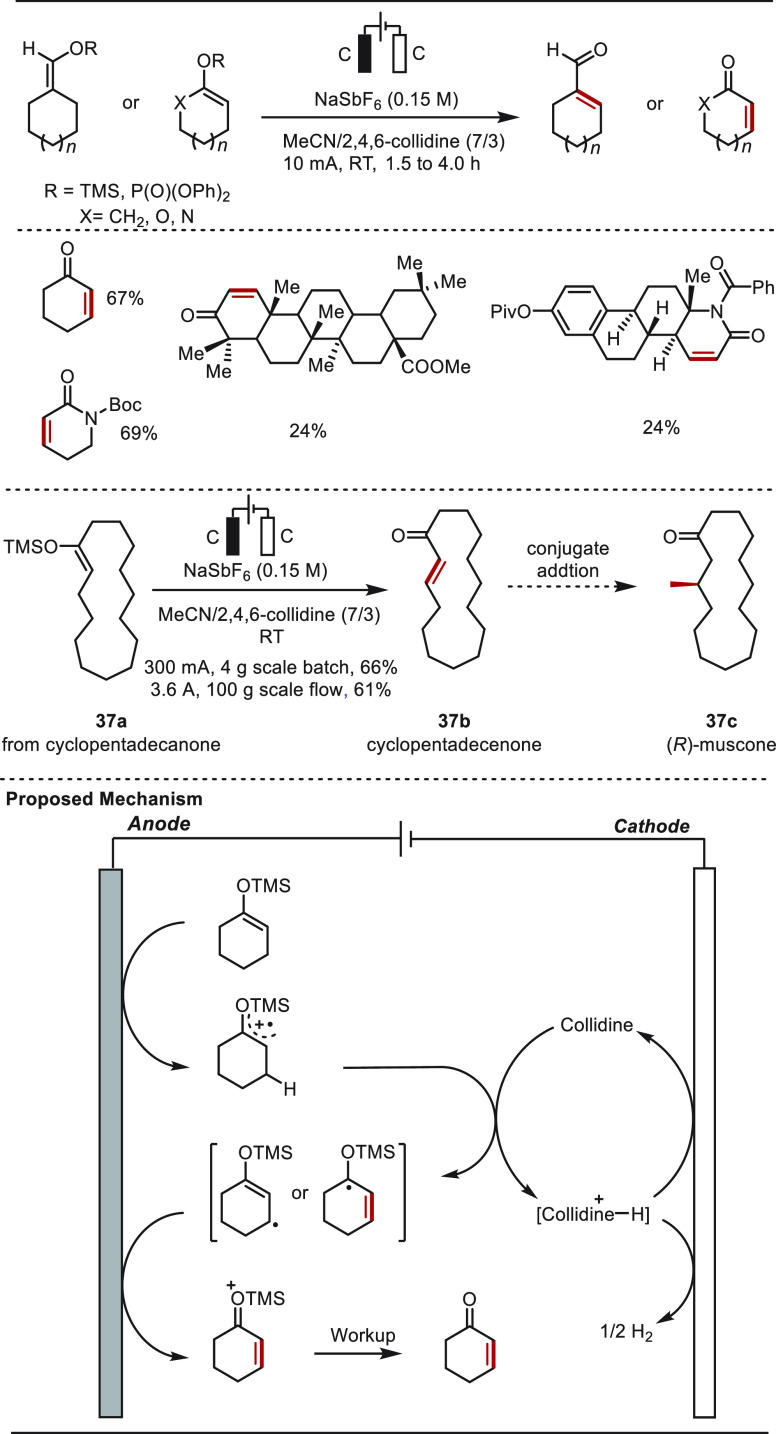
Electrochemically Driven Late-Stage Desaturation of Carbonyl Compounds

Carbonyl desaturation to enone is a fundamental organic oxidation that was widely employed in organic synthesis.^269^ Established approaches to achieve this transformation generally rely on transition metals (Cu or Pd) or stoichiometric oxidative reagents.^270−273^ In 2021, Baran and co-workers disclosed an operationally simple electrochemical method to access such structures from enol phosphates or silanes, which can be readily formed from carbonyls (Scheme 37).^262^ This electrochemically driven desaturation (EDD) was characterized by a broad substrate scope including a variety of ketones and lactams. Notably, the late-stage site-selective desaturation of structurally complex molecules, which is difficult to achieve, afforded the desired enones in synthetically useful yields. In addition, the practical utility of the EDD was further illustrated by the desaturation of 4 g of cyclopentadecanone-derived silyl enol ether **37a** to afford cyclopentadecenone **37b**, which is easily converted to the valuable (*R*)-muscone **37c**. Increasing the current from 10 to 300 mA and using alternating polarity enabled the EDD reaction to smoothly afforded compound **37b** in a 66% isolated yield. By further increasing the current to 3.6 A, 100 g of **37a** was successfully converted into **37b** in a 61% yield in a flow apparatus that contained six reaction cells. Mechanistic studies suggested a radical-based manifold, involving two consecutive single-electron oxidations of enol silane to form oxonium, which released the desired enone after hydrolysis.

**Scheme 38:**
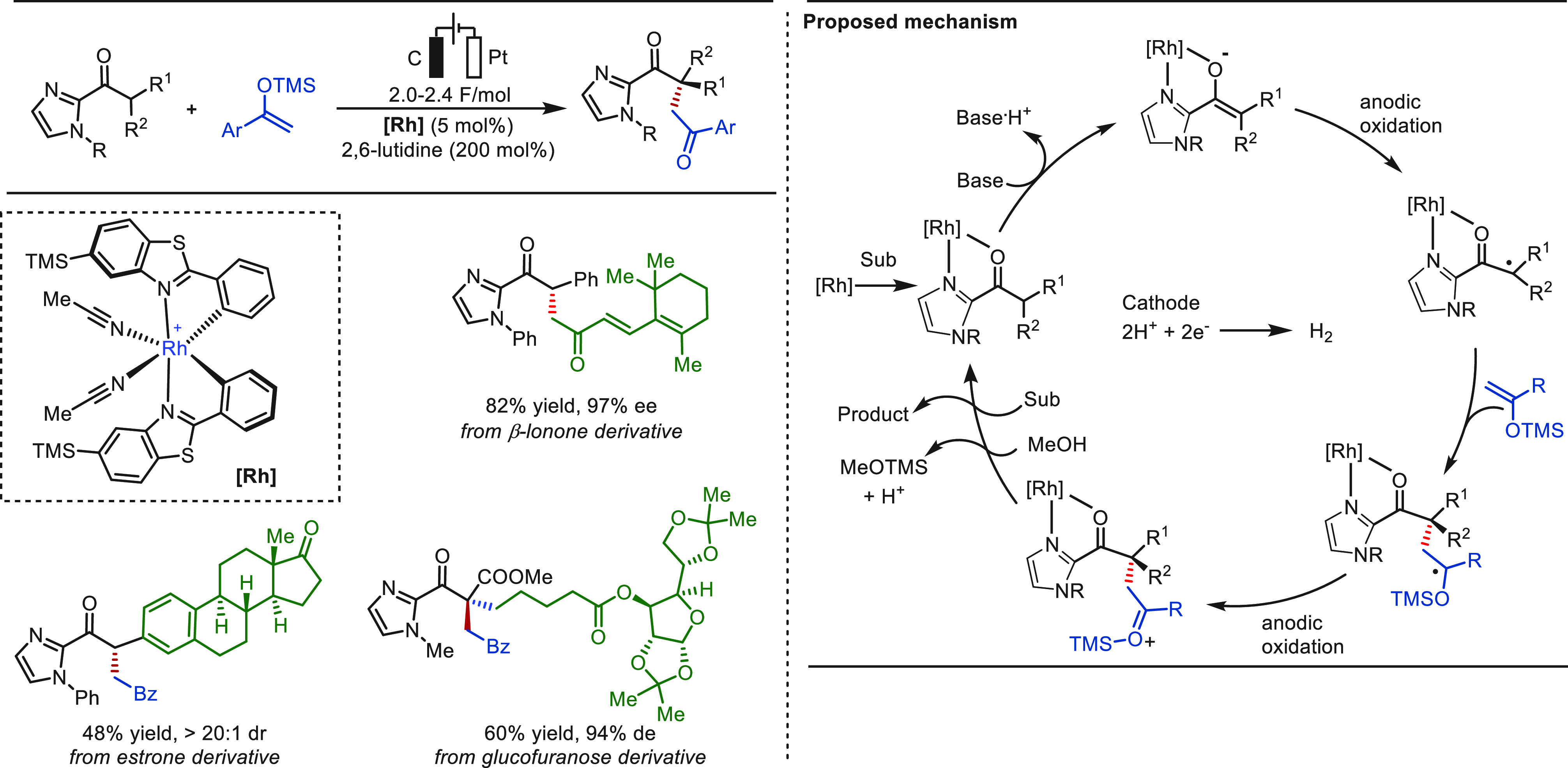
eLSF by α-C(sp^3^)–H Functionalization of Carbonyls

By the merger of organic electrosynthesis with asymmetric catalysis, the Meggers group introduced, in 2019, a versatile electricity-driven chiral Lewis acid catalyzed asymmetric coupling of 2-acyl imidazoles with silyl enol ethers to generate synthetically useful 1,4-dicarbonyls, which include products bearing all-carbon quaternary stereocenters (Scheme 38).^263^ The chiral-at-metal rhodium catalyst played a dual role in both the electrochemical step and to guarantee the asymmetric induction, enabling mild reaction conditions, a broad substrate scope, and high chemo- and enantioselectivities (up to >99% ee). The robustness of this approach is further demonstrated by the effective generation of complex products derived from β-ionone estrone and glucofuranose. Notably, the cleavage of the imidazolyl group could be achieved without a significant loss of optical purity.

**Scheme 39:**
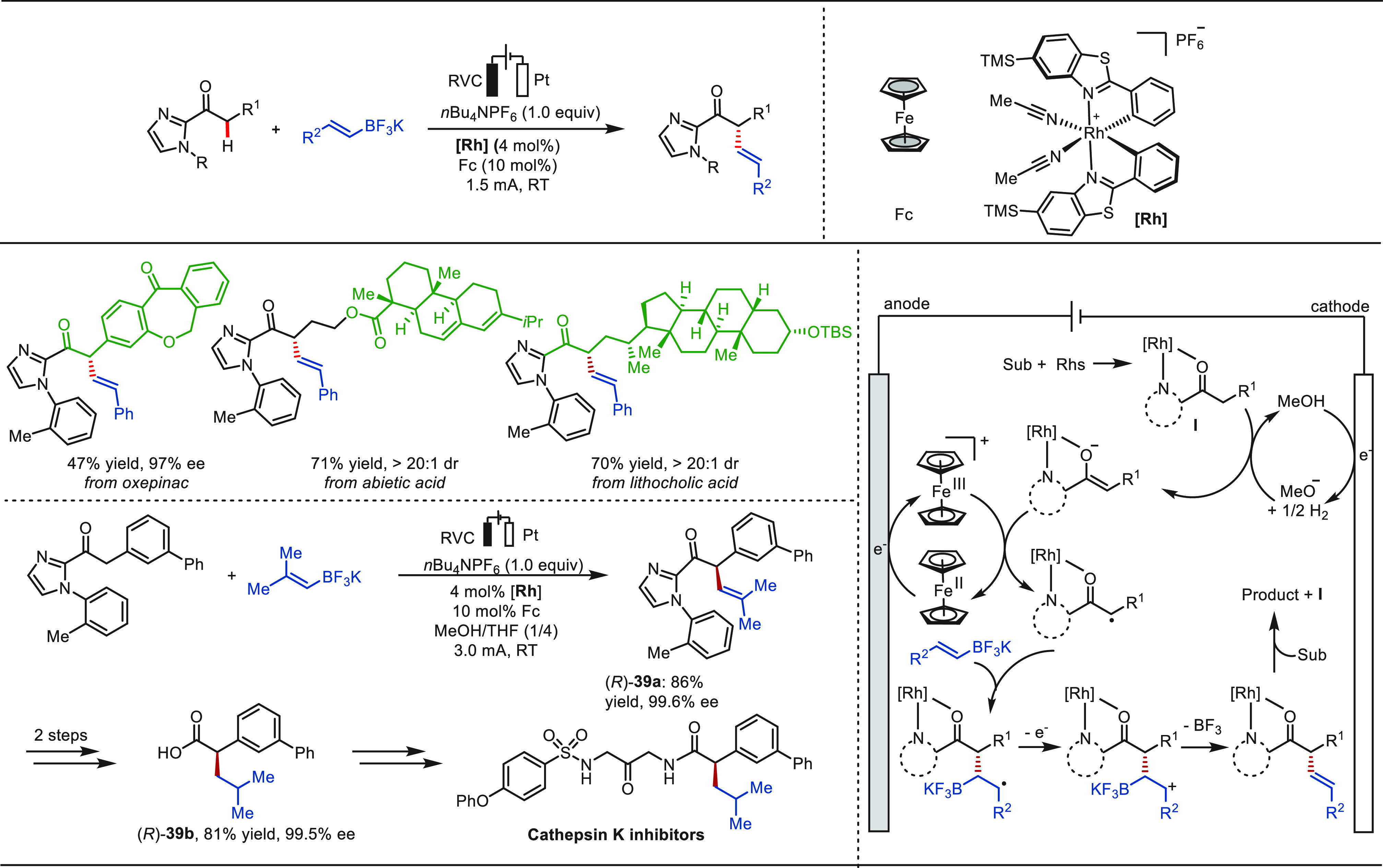
Electrochemical Late-Stage α-C(sp^3^)–H Alkenylation of Carbonyls

Recently, Meggers and co-workers reported another conceptually related approach to achieve enantioselective α-C(sp^3^)–H alkenylation of ketones with potassium alkenyl trifluoroborates (Scheme 39).^264^ The electrochemical asymmetric oxidative coupling reaction features a broad substrate scope, high yields (up to 94%), and exceptional enantioselectivities (≥99% ee). Catalytic amounts of ferrocene were used as the redox mediator, which enables the key chiral rhodium-involving single-electron transfer reaction to homogeneously occur in the solution rather than at the electrode surface, hence providing mild electrochemical conditions. The eLSF alkenylation of complex molecules derived from oxepinac, abietic acid, and lithocholic acid were accomplished in excellent yields via the electricity-driven asymmetric synthesis method. Moreover, this approach was applied to the straightforward assembly of intermediates (*R*)-**39a** of the cathepsin K inhibitor in 86% yield with an *ee* value up to 99.6%.

#### 2.2.4 Late-Stage α-C(sp^3^)–H Functionalization of Amines

**Scheme 40:**
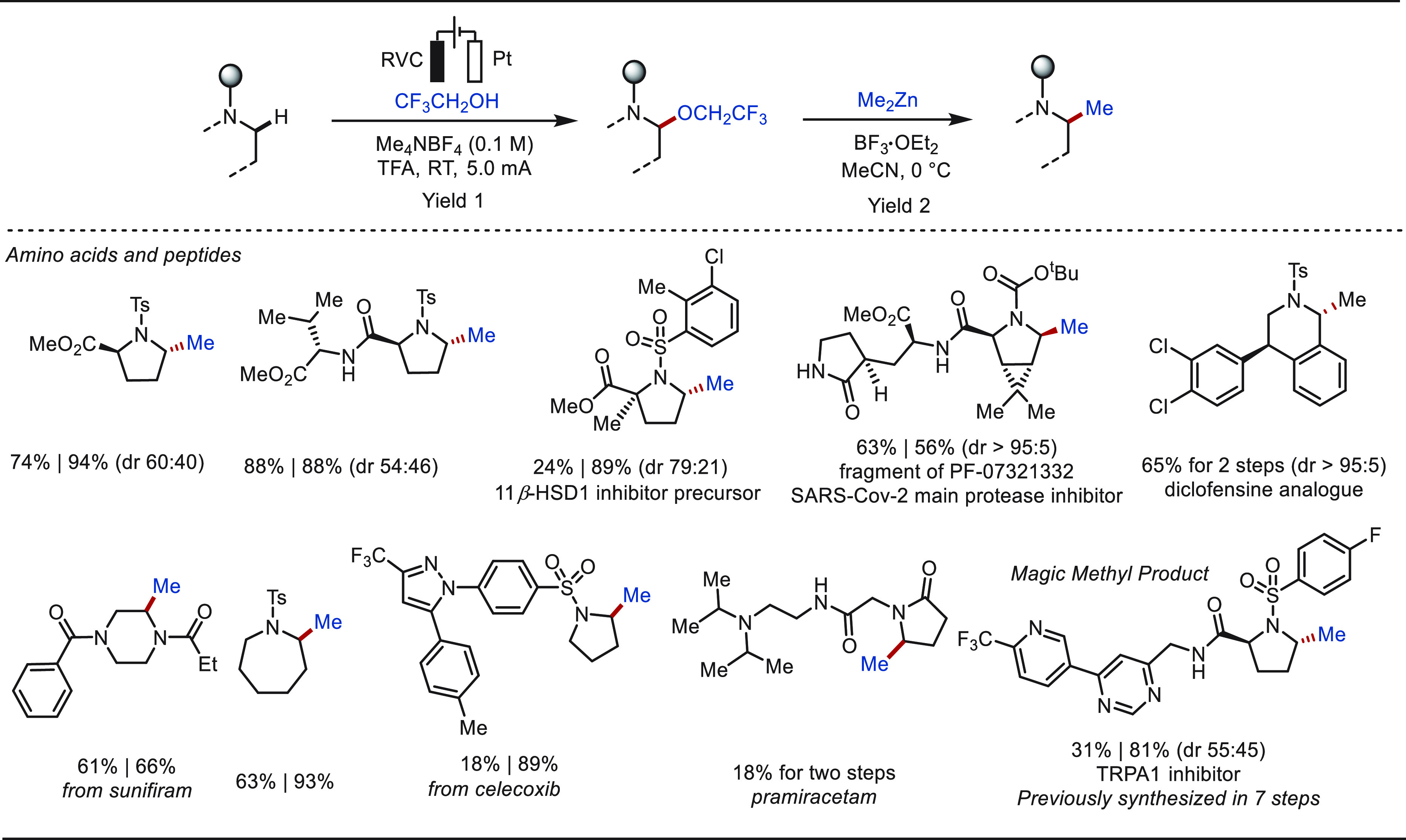
Electrochemical Late-Stage α-Methylation of Amines

The electrochemical functionalization of C(sp^3^)–H bonds adjacent to nitrogen atoms, such as the well-esablished Shono oxidation, has been wildly applied in organic synthesis.^274,275^ However, until recently, it was primarily employed for the functionalization of structurally simple compounds. Based on the classic Shono oxidation reaction, Lin and Terrett recently reported a modular and practical strategy for eLSF α-methylation of structurally complex amines derivatives (Scheme 40).^276^ The electro-oxidation generated *N*,*O*-acetal readily reacted with organozinc reagents, enabling the facile installation of a methyl moiety as well as various other important groups. This improved electrochemical protocol features operational simplicity and high functional group compatibility. The site-selective late-stage methylation of a variety of bioactive targets, has been efficiently achieved. Notably, a drug molecule of TRPA1 inhibit, which has been explored for the “magic methyl” effect, presenting a >10-fold boost in potency, was previously synthesized using *de novo* routes in a total of 7 steps.^277^ In sharp contrast, this compound could be readily prepared from its parent inhibitor by this electron driven approach.

**Scheme 41:**
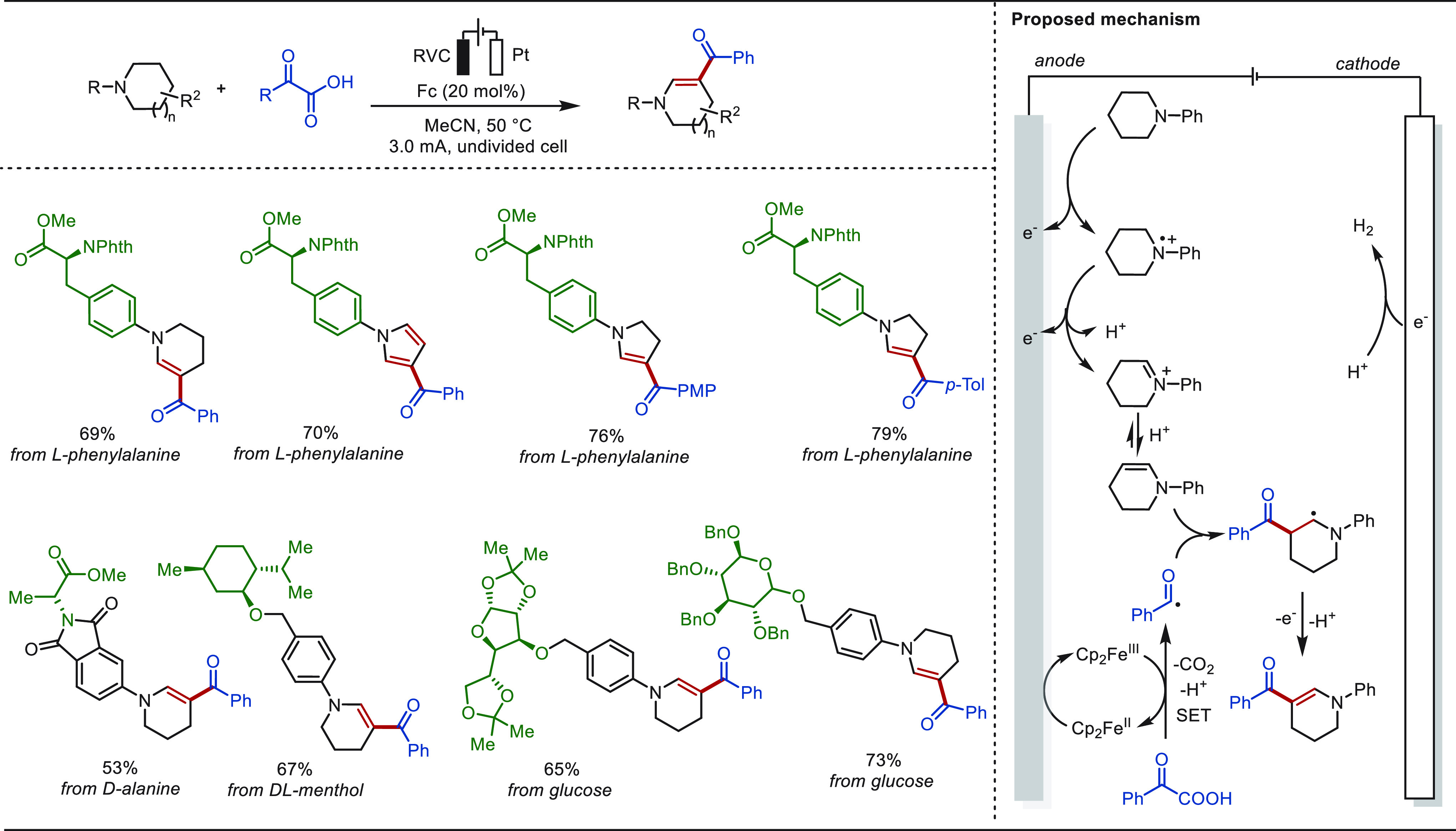
Electrochemically Driven Desaturative β-C(sp^3^)–H Functionalization of Amines

Electrochemical dehydrogenation based on the α-C(sp^3^)–H activation of amines represent an important organic transformation to ubiquitous unsaturated compounds.^278−283^ In 2018, the Lei group disclosed a TEMPO-mediated dehydrogenation of *N*-heterocycles in an undivided cell to access a variety of five- and six-membered nitrogen-heteroarenes without the usage of sacrificial hydrogen acceptors.^282^ Recently, Qiu and co-workers reported a straightforward and robust approach of electrochemically driven desaturative β-C(sp^3^)–H functionalization of cyclic amines (Scheme 41).^284^ Various β-substituted desaturated cyclic amines were obtained under constant current electrolysis in MeCN at 50 °C. This transformation was achieved via multiple single-electron oxidation processes with catalytic amounts of ferrocene as a redox mediator. The unique utility of this approach was clearly demonstrated by the eLSF of natural products and derivatives (Scheme 41). Diverse pyrrolidine- or piperidine-containing molecules deriving from l-phenylalanine, d-alanine, d,l-menthol, glucofuranose, and glucopyranose afforded the corresponding desaturated acylation products in excellent yields. Notably, the reaction of l-phenylalanine bearing a pyrrolidine motif with phenylacetic acid formed a pyrrole product through further electro-oxidation.

#### 2.2.5 Late-Stage C(sp^3^)–H Functionalization of Sulfides

**Scheme 42:**
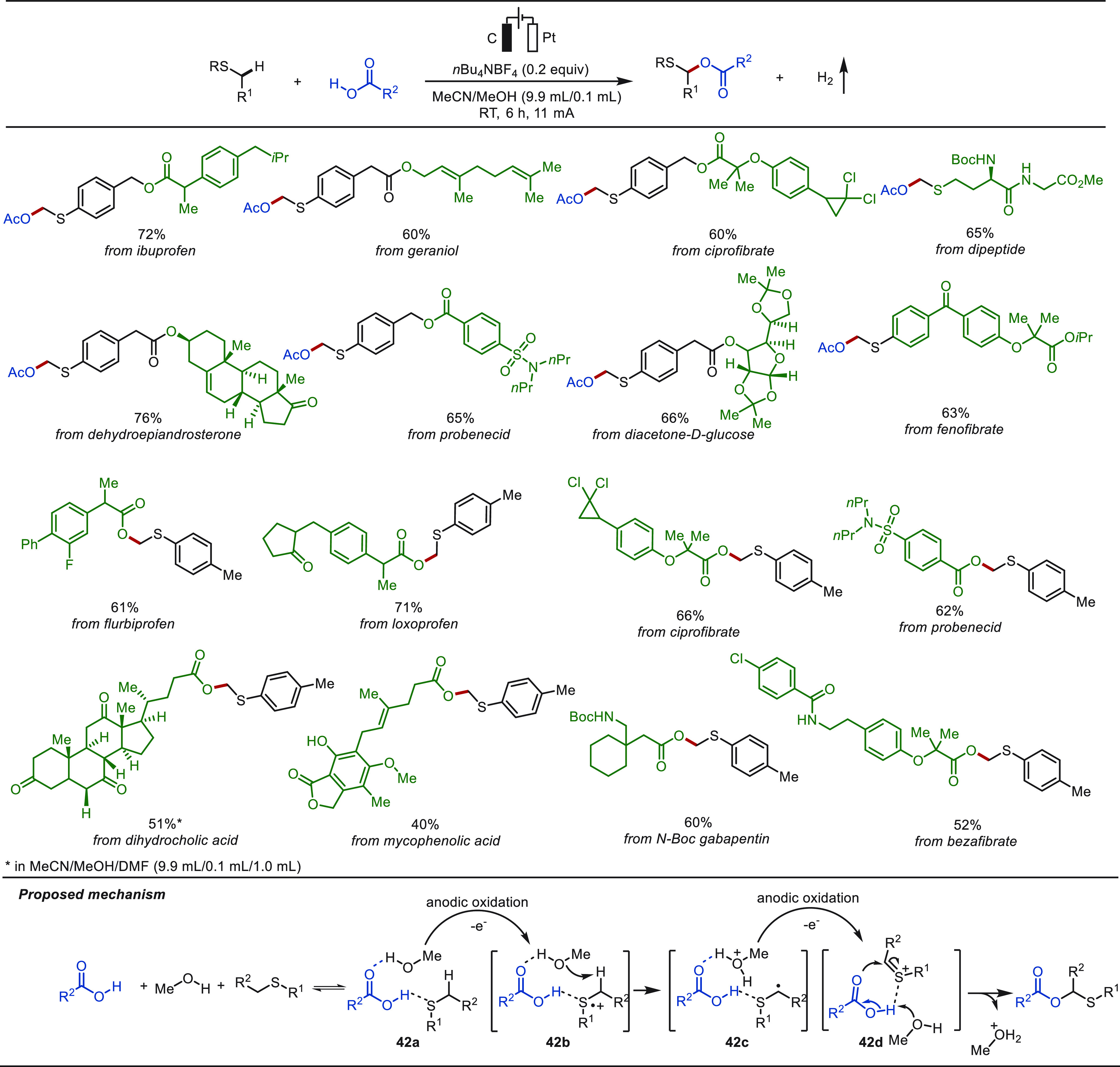
Electrochemical Late-Stage α-C(sp^3^)–H Acyloxylation of Sulfides

The precise and selective activation of oxidation-sensitive sulfur-containing compounds is a significant challenge due to its inherent activity and complicated valence states.^285^ In 2021, Lei and co-workers reported on an electrochemical protocol for the construction of α-acyloxy sulfides, which represent key structural motifs in agrochemicals and pharmaceuticals (Scheme 42).^286^ This electro-oxidized C(sp^3^)–H/O–H cross-coupling protocol was found to be environmentally friendly, highly selective, and scalable while featuring an exceptionally broad substrate scope. The robustness and utility of this protocol was demonstrated by the efficient eLSF of a wealth of bioactive molecules, including amino acids, peptides, and pharmaceuticals. Mechanistic studies suggested a synergistic effect of the self-assembly induced C(sp^3^)–H/O–H coupling pathway. Sulfide, AcOH, and MeOH assemble into an adduct, and hydrogen bonding between AcOH and the sulfur atom can facilitate a SET oxidation of sulfide. MeOH selectively captures the proton to form the state **42c** with high regioselectivity. Then, a thionium ion is generated via the loss of a proton and an electron, and the desired product is finally delivered after the nucleophilic attack of AcOH to the thionium ion.

#### 2.2.6 Late-Stage Unactivated C(sp^3^)–H Functionalization

Unactivated C(sp^3^)–H bonds generally feature a high redox potential of more than 3.0 V vs SCE.^287^ Thus, the direct electrolysis of the C(sp^3^)–H bond represents a formidable challenge, since oxidation of other functionalities or solvents is likely to occur prior to the desired C–H oxidation of simple alkanes. Despite the difficulties, scientists have made remarkable progress in electrochemical late-stage functionalization of unactivated C(sp^3^)–H bond by the use of redox mediators or transition-metal catalysts.^288,289^

**Scheme 43:**
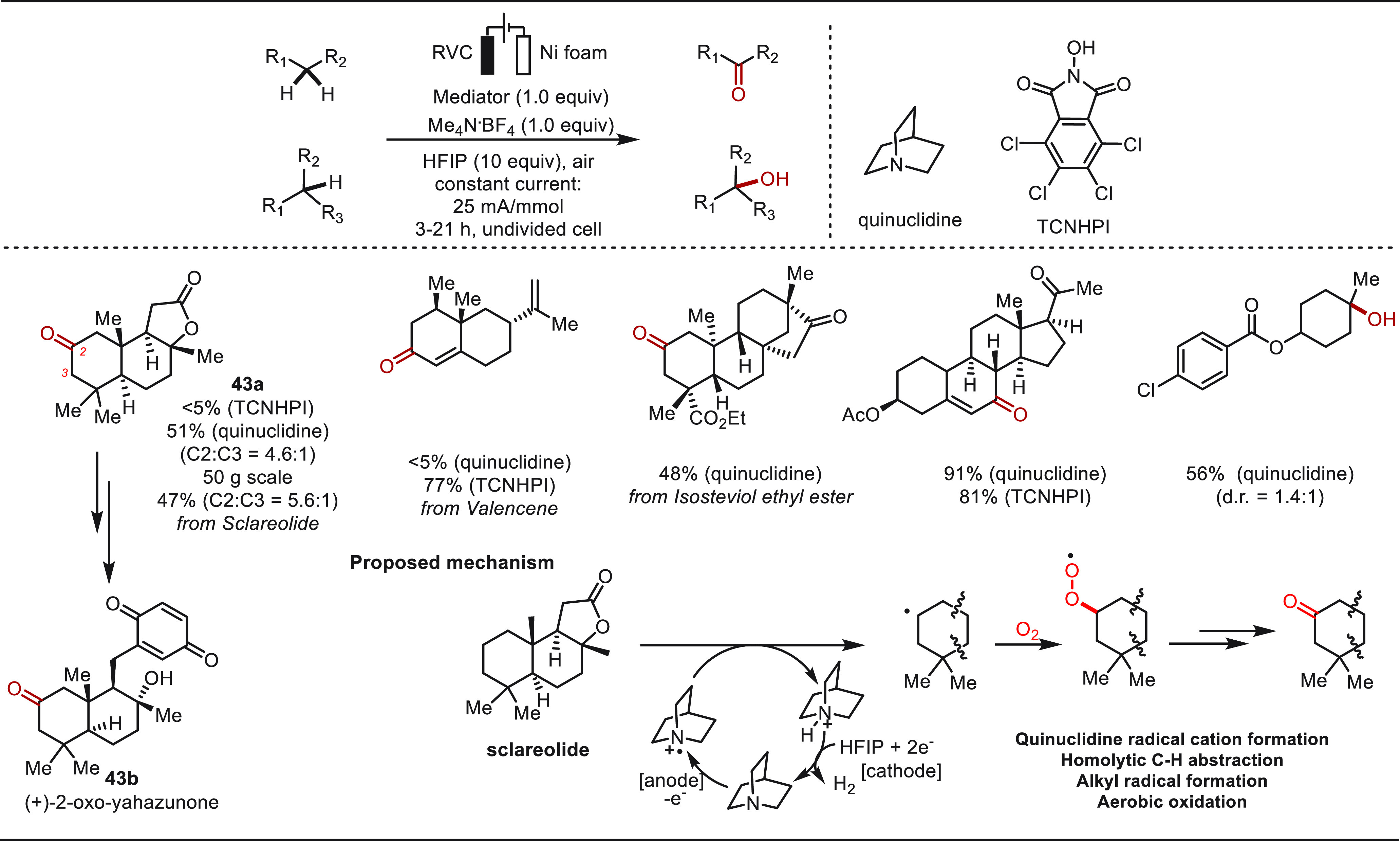
Electrochemical Late-Stage Oxidation of Unactivated C(sp^3^)–H Bonds

In 2017, the Baran group presented a practical electrochemical oxidation of otherwise unactivated C–H bonds in MeCN with Me_4_NBF_4_ as the electrolyte and HFIP as the additive (Scheme 43).^288^ Identification of a suitable redox mediator was the key to success for high yields and chemoselectivities. While using quinuclidine as a mediator allowed the selective late-stage oxidation of Sclareolide in a 51% yield at ca. 1.8 V vs SCE Ag/AgCl, the use of TCNHPI as mediator is superior on those bearing multiple olefin motifs, such as valencene. The quinuclidine-based redox mediator system further proved compatible for the eLSF of isosteviol ethyl ester and oxidation of a terpene to a relative steroid. In addition, a tertiary C–H bond is efficiently oxidized to the corresponding alcohol under the quinuclidine-mediated electrolysis. The utility of this protocol was illustrated with a 50 g scale late-stage oxidation of sclareolide to **43a**, which is a key intermediate for the synthesis of (+)-2-oxo-yahazunone. Mechanistically, the in situ anodic oxidation generated quinuclidine radical cation served as a HAT reagent and O_2_ was involved in the later aerobic oxidation step.

**Scheme 44:**
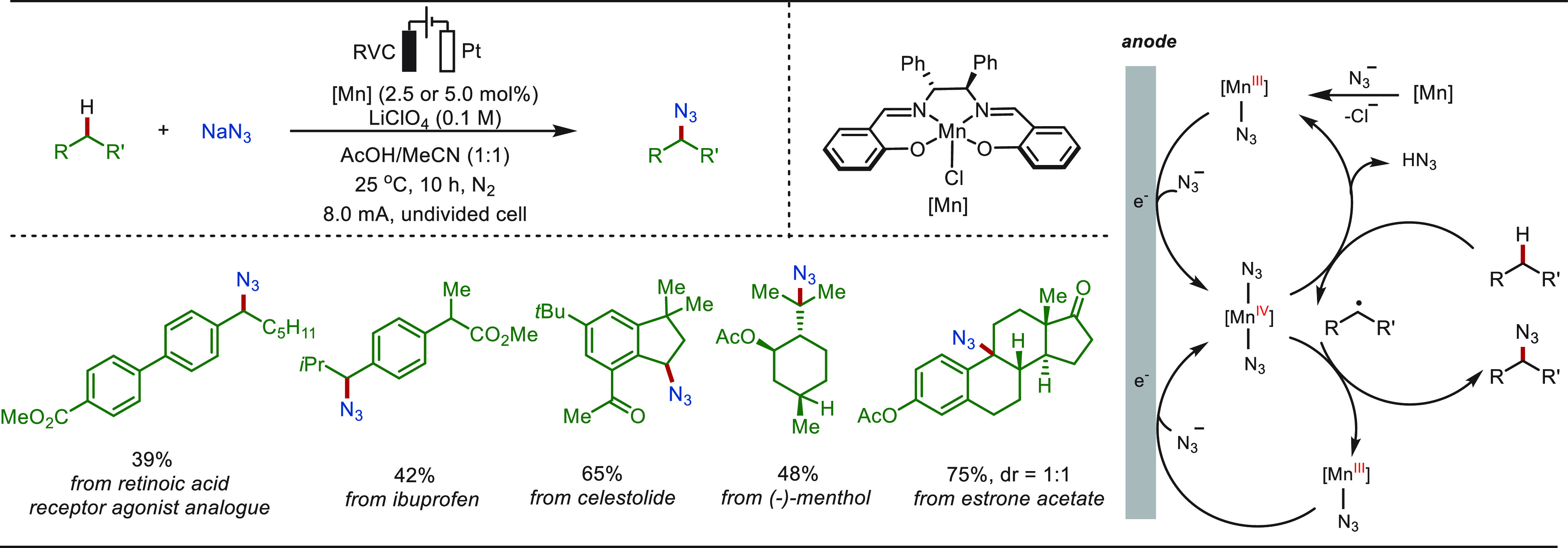
Electrochemical Late-Stage C(sp^3^)–H Azidation

Organic azides are key intermediates for numerous transformations in medicinal chemistry, peptide chemistry, or molecular biology.^290−292^ Traditionally, stoichiometric amounts of strong indiscriminate chemical oxidants, such as NFSI and hypervalent iodine reagents, are required to install the azido group into C(sp^3^)–H bonds. By contrast, in 2021, the Ackermann group disclosed a manganaelectro-catalyzed C–H azidation of otherwise unactivated C(sp^3^)–H bonds with most user-friendly NaN_3_ as the nitrogen-source and traceless electrons as the sole redox-reagent (Scheme 44).^289^ The robustness and practicability of the resource-economic method was highlighted by the eLSF azidation of a variety of bioactive molecules. Detailed mechanistic studies supported a unique manganese(III/IV) regime, avoiding overoxidation to the carbocation and thus suppressing undesired side-reactions to oxygenated products.

The merger of electrochemistry with organometallic catalysis has shown significant advances in C(sp^3^)–H activation. For example, Mei and Sanford, respectively, have achieved unactivated C(sp^3^)–H bond oxygenation by palladium catalysis under electrochemical conditions.^293,294^ This strategy is expected to be used in the late-stage modification of bioactive molecules in the future.

### 2.3 eLSF of C(sp)–H Bonds

**Scheme 45:**
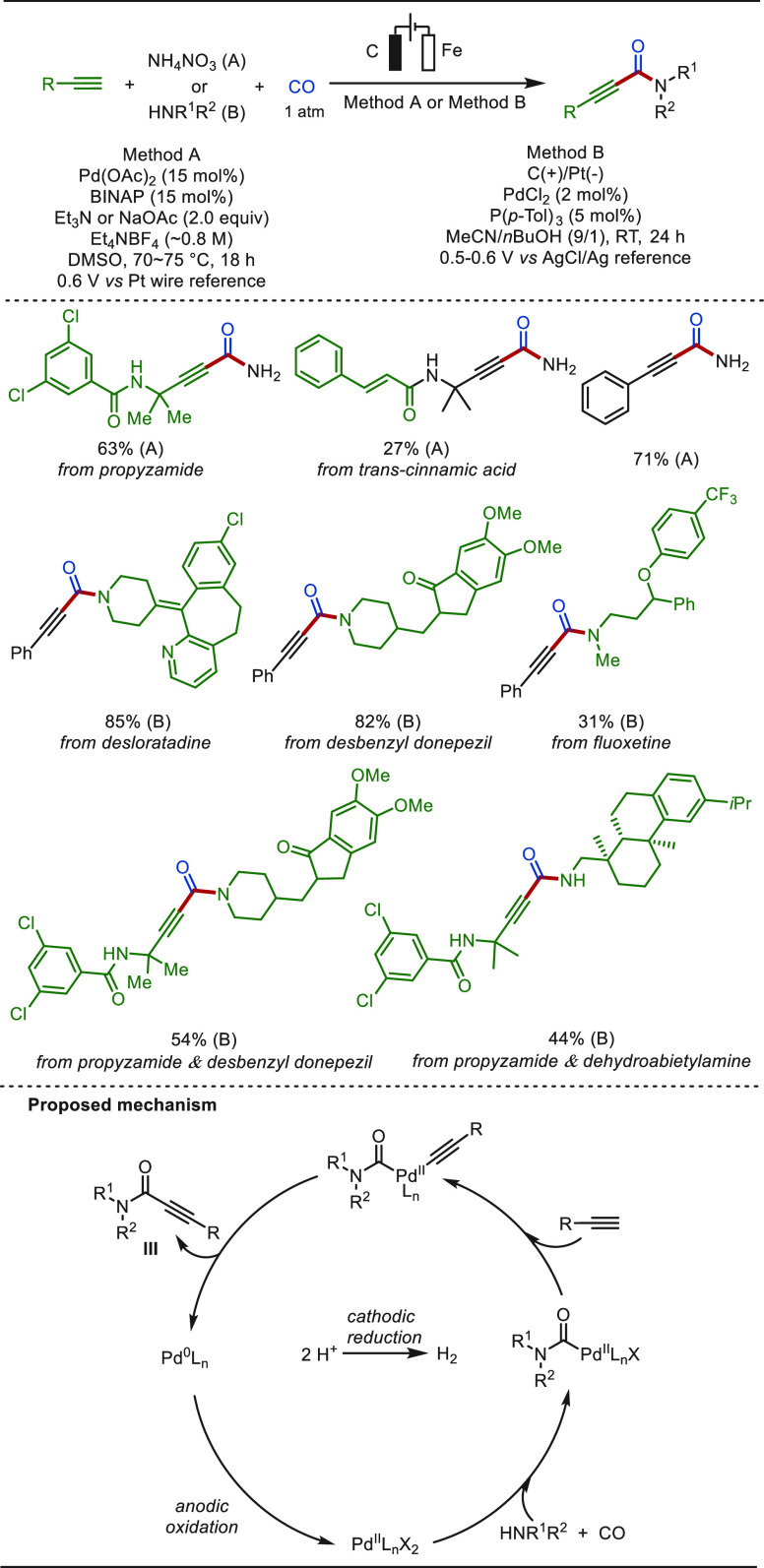
Electrochemical Palladium-Catalyzed Aminocarbonylation of Terminal Alkynes

Oxidative carbonylation of alkynes represents an important transformation in molecular synthesis that generally uses O_2_ as the oxidant. However, the explosibility of gas mixtures of CO/O_2_ (12.5–74.0%) deters scalable application of this process. In 2019, the Lei group disclosed an electro-oxidative palladium-catalyzed carbonylation of alkynes to 2-ynamides under copper- and O_2_-free conditions (Scheme 45). This transformation occurred under potentiostatic conditions, and the role of the current was to oxidize Pd(0) to Pd(II), which was also the rate-determining step of this process. The eLSF of propyzamide with CO (1 atm) and NH_4_NO_3_ furnished corresponding propiolamide in a 63% yield. Primary and secondary amines proved to be amenable for this electrochemical aminocarbonylation reaction. Drug molecules, including desloratadine, fluoxetine, and desbenzyl donepezil, smoothly underwent eLSF, affording desired 2-ynamides in satisfactory yields.

**Scheme 46:**
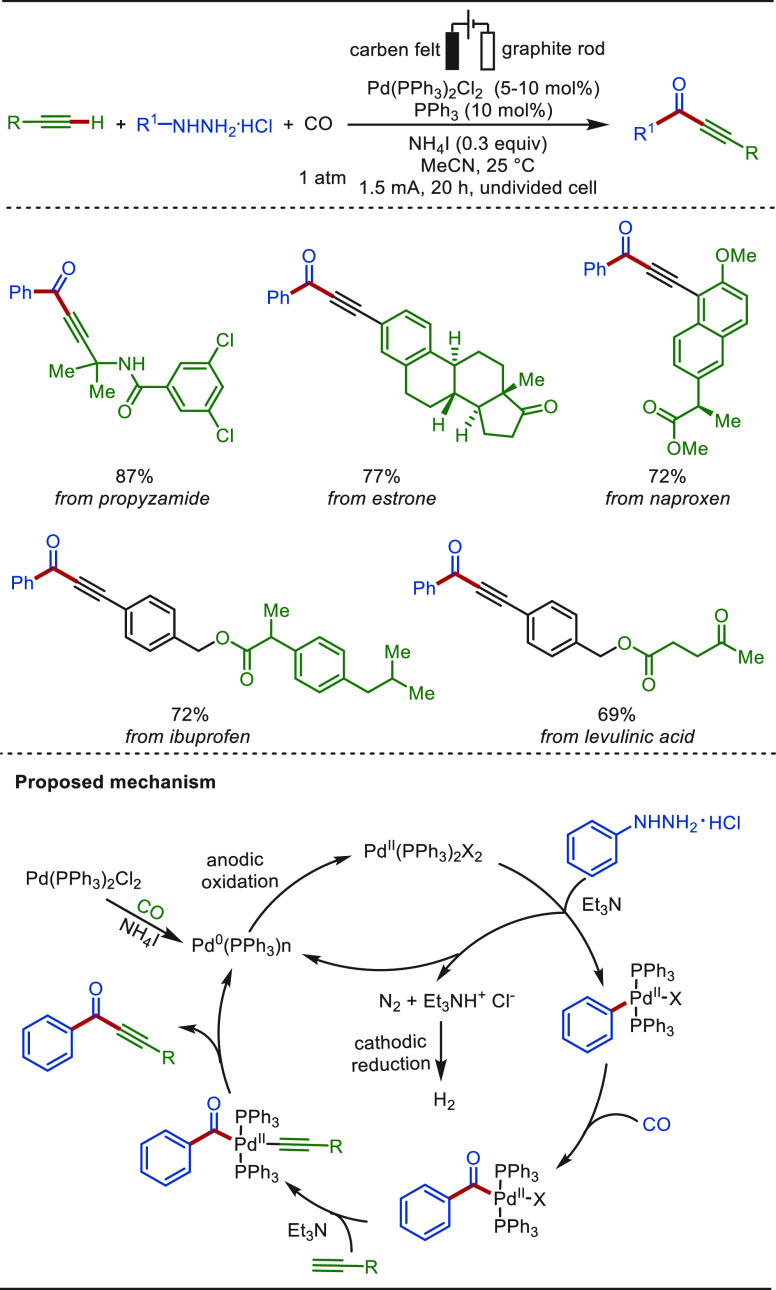
Electrochemical Palladium-Catalyzed Oxidative Sonogashira–Hagihara Carbonylation of Arylhydrazines and Alkynes

In 2021, by employing arylhydrazines instead of amines, Lei and co-workers further accomplished the electrochemical palladium-catalyzed oxidative carbonylation of alkynes to synthesize ynones, which is an alternative supplement of the carbonylative Sonogashira–Hagihara reaction (Scheme 46).^295^ The LSF of bioactive molecules deriving from propyzamide, estrone, naproxen, ibuprofen, and levulinic acid afforded the corresponding ynones in excellent yields. Similarly, the process occurs via a proposed palladium(0)/palladium(II) regime, and the use of current as oxidant avoids the explosion hazard of CO.

**Scheme 47:**
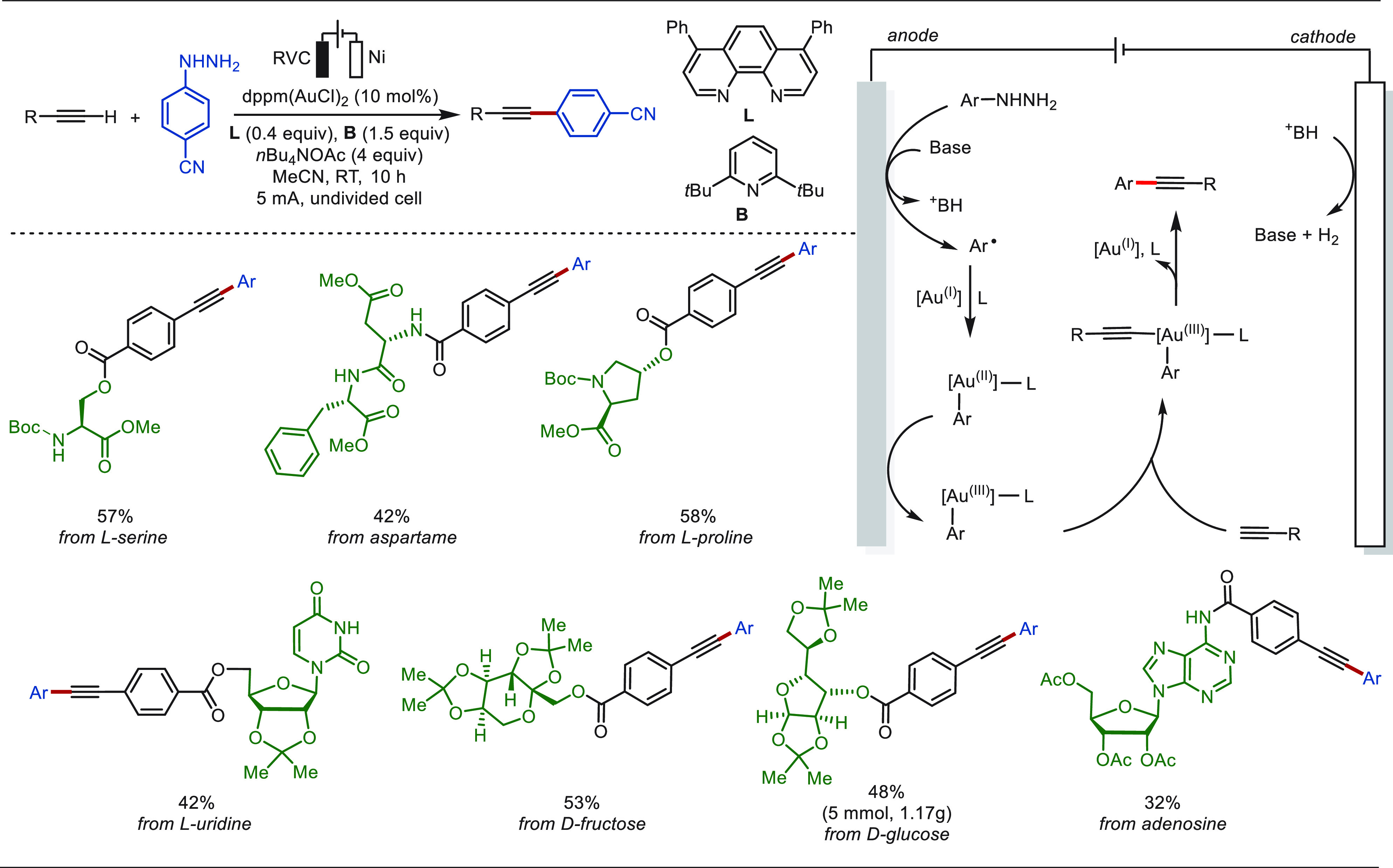
Electrochemical Gold-Catalyzed Oxidative C(sp)–C(sp^2^) Coupling

Recently, Xie and co-workers disclosed a electrochemical gold-catalyzed C(sp)–C(sp^2^) coupling reaction between structurally complex alkynes and arylhydrazines (Scheme 47).^296^ This approach exhibited broad functional group tolerance without the use of chemical oxidants. The robustness of this approach was further illustrated by the efficient late-stage modification of a variety of alkynes tethered to biomolecules. Mechanistic studies suggested the anodic oxidation of aromatic hydrazine to generate an aryl radical, which recombined with gold(I) and underwent further anodic oxidation to form the Ar–Au(III) species for subsequent σ-activation of alkynes.

## 3 eLSF of Functional Groups

Electrocatalytic interconversion of common organic functionalities bears unique potential for the advancement of organic synthesis. Interestingly, owing to their robust and mild conditions, these approaches are often adopted for the late-stage derivatization of complex organic molecules. In the following section, we will discuss the progress in the area of electrochemical late-stage functional group modification strategies.

### 3.1 eLSF of Alkenes and Alkynes

Olefins are prevalent structural motifs in various biologically relevant molecules and natural products.^297,298^ These moieties are quite reactive and are often garnered for incorporating new functional groups in the molecule.^299−306^ Synthetic manipulation of these substructures is thus used as a versatile strategy for late-stage functionalization reactions.

**Scheme 48:**
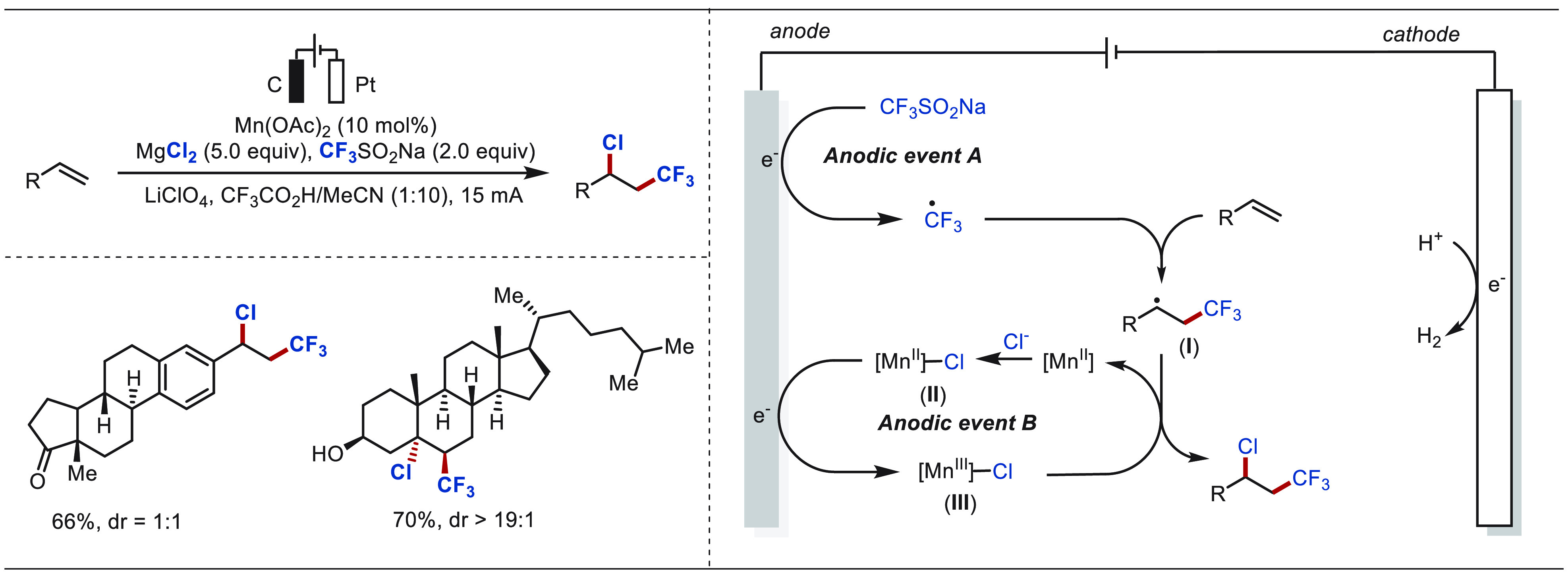
Electro-oxidative Chlorotrifluoromethylation of Olefins

The Lin group has done great contributions in the field of metallaelectro-catalyzed functionalization of alkenes.^301,302,304−309^ In 2018, Lin described an electro-oxidative heterodifunctionalization of olefins enabled by anodic oxidation of CF_3_SO_2_Na (Scheme 48).^307^ The interception of the anodically generated trifluoromethyl radical with a terminal olefin formed a secondary alkyl radical intermediate, which was trapped with a chloride radical to form the heterodifunctionalized product. The use of catalytic Mn(OAc)_2_ assisted the electrochemical process through the formation of an alleged Mn(III)-Cl radical chlorinating agent, which helped the chloride radical recombination step. This anodically coupled electrocatalytic process was exploited for the late-stage functionalization of several natural product analogues.

**Scheme 49:**
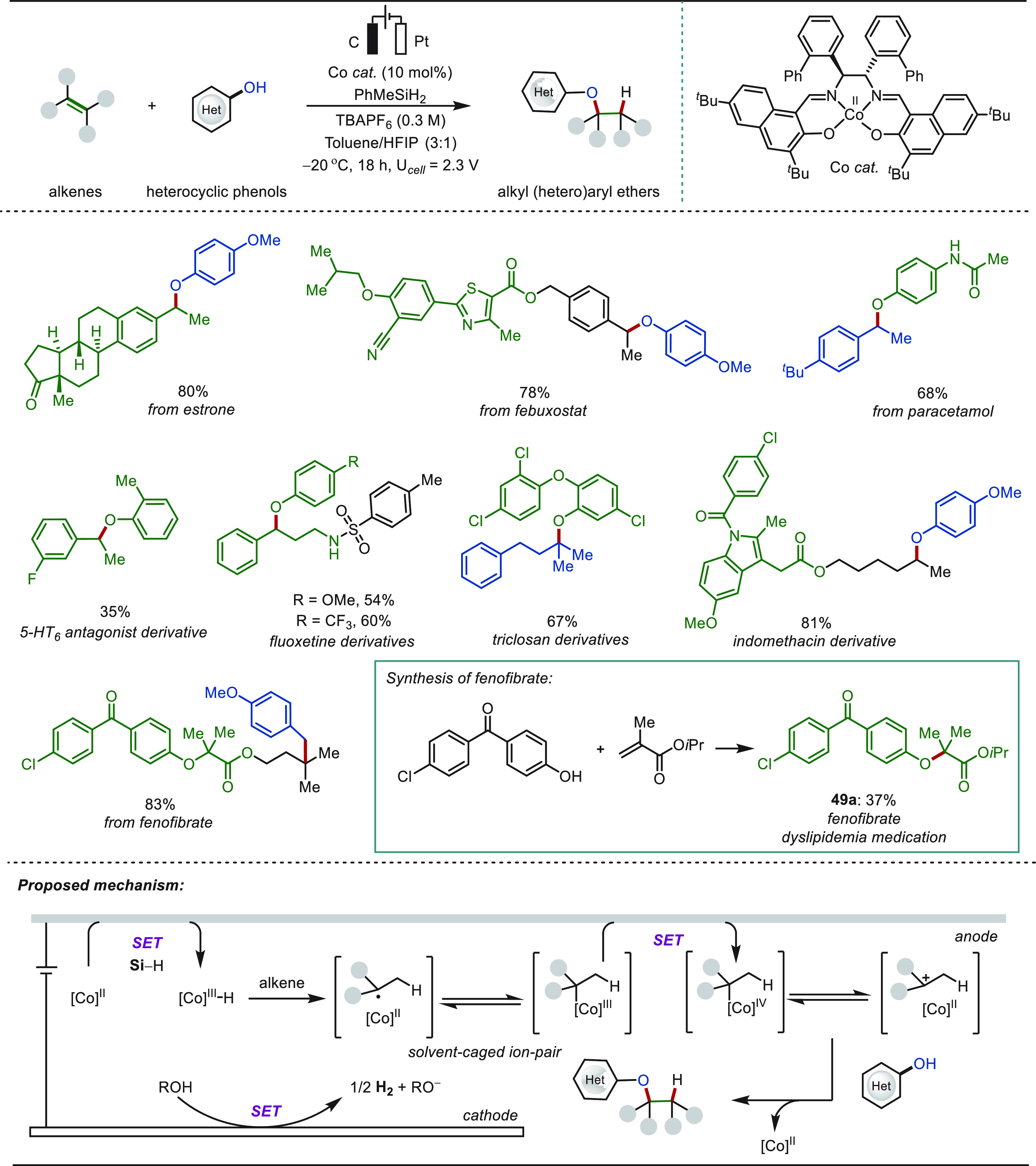
Electrochemical Cobalt-Catalyzed Late-Stage Hydroetherification of Olefins

Later, a cobalt-salen-catalyzed hydroetherification strategy was demonstrated by Kim and Shin combining MHAT and anodic oxidation (Scheme 49).^310^ Generally, in MHAT strategies, weak nucleophiles exhibit poor reactivity owing to the formation of a “solvent-caged radical pair”, which deflates the nucleophilic entrapment process. Anodic oxidation of the caged intermediate detoured the detrimental bimetallic disproportionation pathway and enabled the nucleophilic displacement process. The electrocatalysis involved a plausible cobalt(II/III/IV) pathway for product formation. This versatile strategy was employed for the late-stage hydroetherification of estrone, febuxostat, paracetamol, fluoxetine, triclosan, indomethacin derivatives including many other important organic molecules. The versatility of the hydroetherification strategy was also highlighted through the synthesis of fenofibrate **49a**, an oral medication for dyslipidemia.

**Scheme 50:**
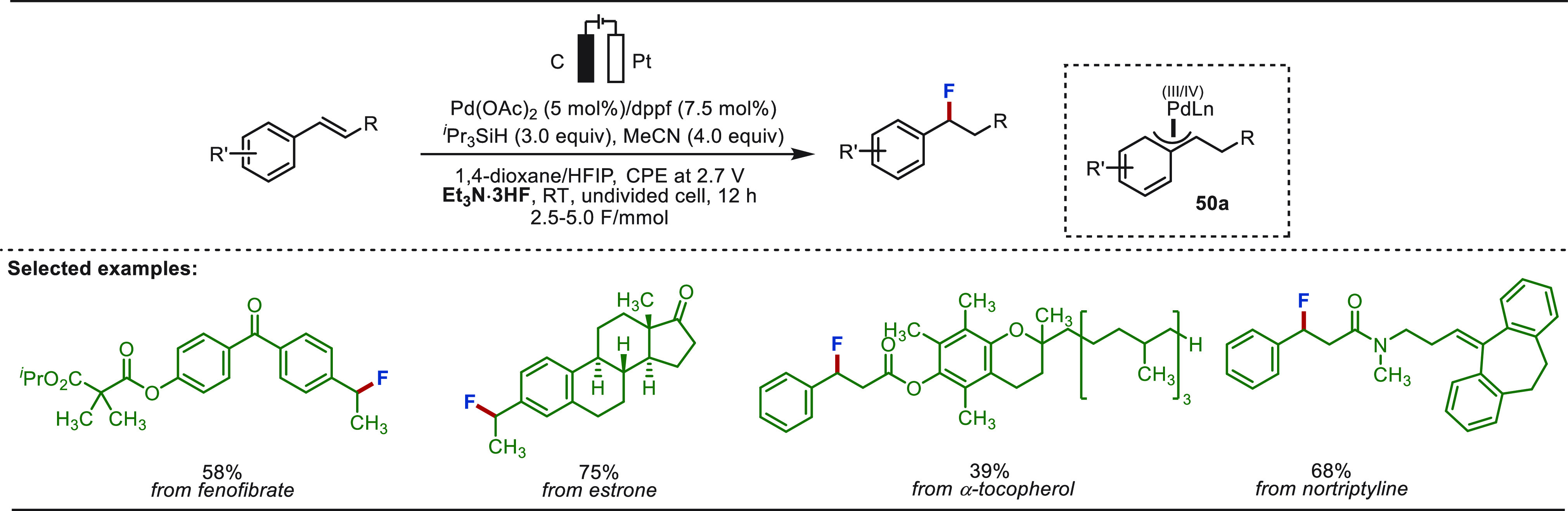
Electrochemical Palladium-Catalyzed Late-Stage Hydrofluorination of Olefins

They further illustrated an electro-oxidative palladium-catalyzed approach very recently to realize benzylic fluorinations in a straightforward manner using Et_3_N·3HF as a nucleophilic fluorinating agent (Scheme 50).^311^ Similar to the prior findings, this strategy operated through a metal hydride intermediate, which after migratory insertion with the olefin formed a high-valent η^3^-benzylpalladium intermediate. This intermediate under electro-oxidative conditions guided a nucleophilic displacement reaction with the nucleophilic fluorinating agent. This hydrofluorination strategy employed the dppf ligand and silane as the hydride source. The formation of intermediate **50a** was confirmed by cyclic voltammetry studies. This approach was employed for the selective benzylic fluorination of biologically relevant nortriptyline, fenofibrate, estrone, and α-tocopherol derivatives.

**Scheme 51:**
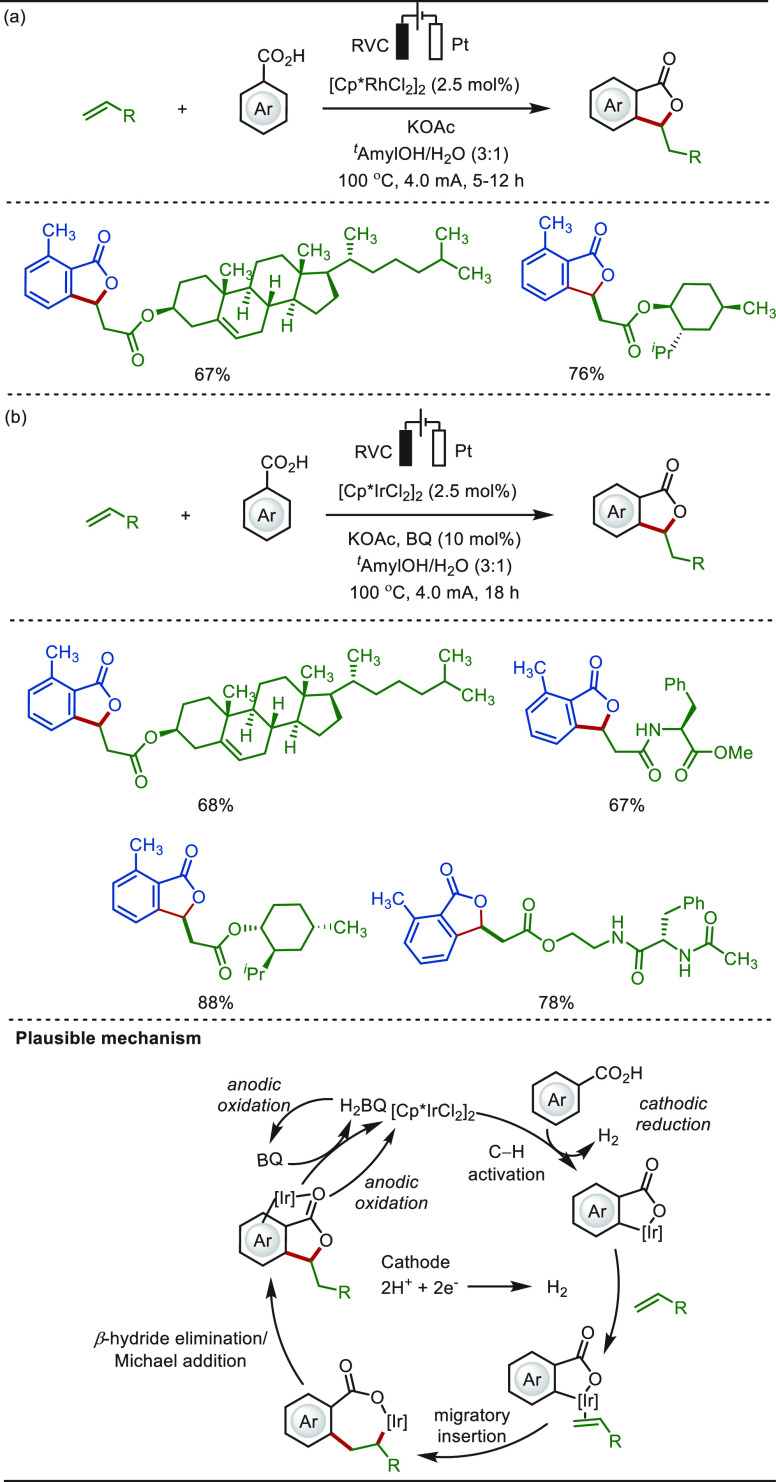
Electro-oxidative Late-Stage Annulation of Biologically Relevant Olefins

In 2018, Ackermann reported the versatile electro-oxidative olefination/annulation approach under rhodium and iridium catalysis, respectively (Scheme 51).^312,313^ These chemo- and site-selective strategies harvested electricity as the renewable terminal oxidant, converting easily accessible aromatic carboxylic acids to synthetically meaningful phthalides. Various acrylate analogues embracing naturally occurring complex terpenoids and amino acids were compatible to the reaction conditions generating respective phthalides in high yields. While the rhoda-electrocatalyzed approach relied on direct anodic oxidation to regenerate the rhodium(III)-catalyst, catalytic amounts of benzoquinone redox-mediator were necessary to promote an iridium-catalyzed transformation.

**Scheme 52:**
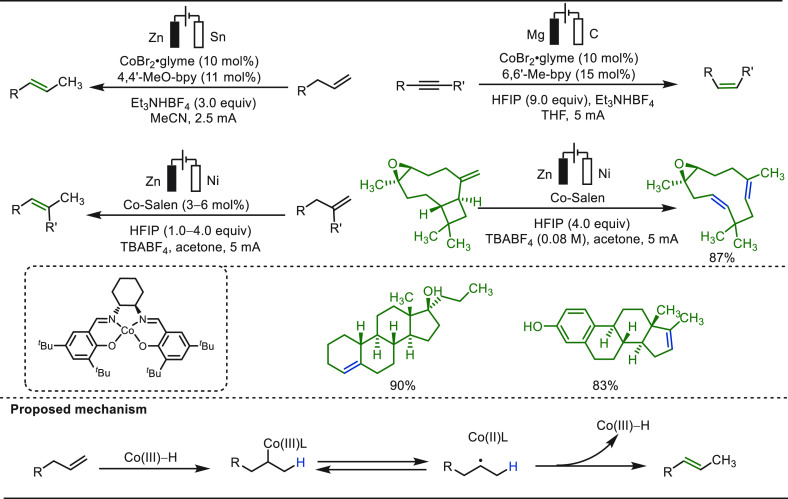
Electroreductive Cobalt-Catalyzed Late-Stage Functionalization of Olefins and Alkynes

The controlled isomerization of readily available terminal alkenes or reduction of alkynes is an effective and practical strategy to access internal olefins.^314−319^ Recently, Baran and co-workers established an electroreductive Co-catalyzed regioselective olefin isomerization strategy harnessing transition-metal hydride intermediates (Scheme 52).^320^ The cathodic reduction of high-valent Co(III)-species formed low-valent Co(I)-species, which can effectively reduce protons to form a Co(III)–H intermediate. This cobalt hydride intermediate when reacted with terminal olefins and alkynes, it selectively transformed them into corresponding internal olefins and *Z*-olefins, respectively. This simple and straightforward method proved to be applicable for the modification of a variety of substrates including the late-stage derivatization of structurally complex organic architectures.

**Scheme 53:**
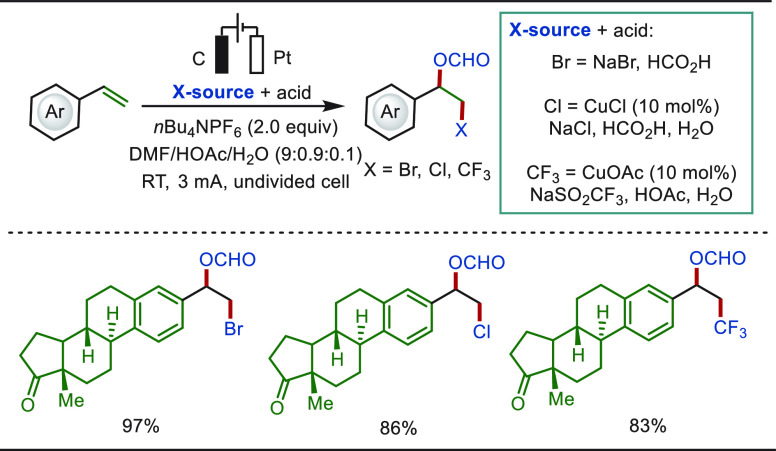
Late-Stage Difunctionalization of Steroid Derivatives

The electrochemical functionalization of alkenes under transition-metal-free conditions has also been extensively studied. In 2019, Fang and Hu reported a scalable difunctionalization of olefins harnessing anodic oxidation, in which the reaction presumably proceeded through a nucleophilic addition of dimethylformamide to the benzylic carbocation, formed after anodic oxidation of a benzylic radical (Scheme 53).^321^ This approach allowed for the bromination, chlorination, and trifluoromethylation-formyloxyation of naturally occurring steroids using bench-stable NaBr, NaCl, and NaSO_2_CF_3_ as corresponding radical sources.

**Scheme 54:**
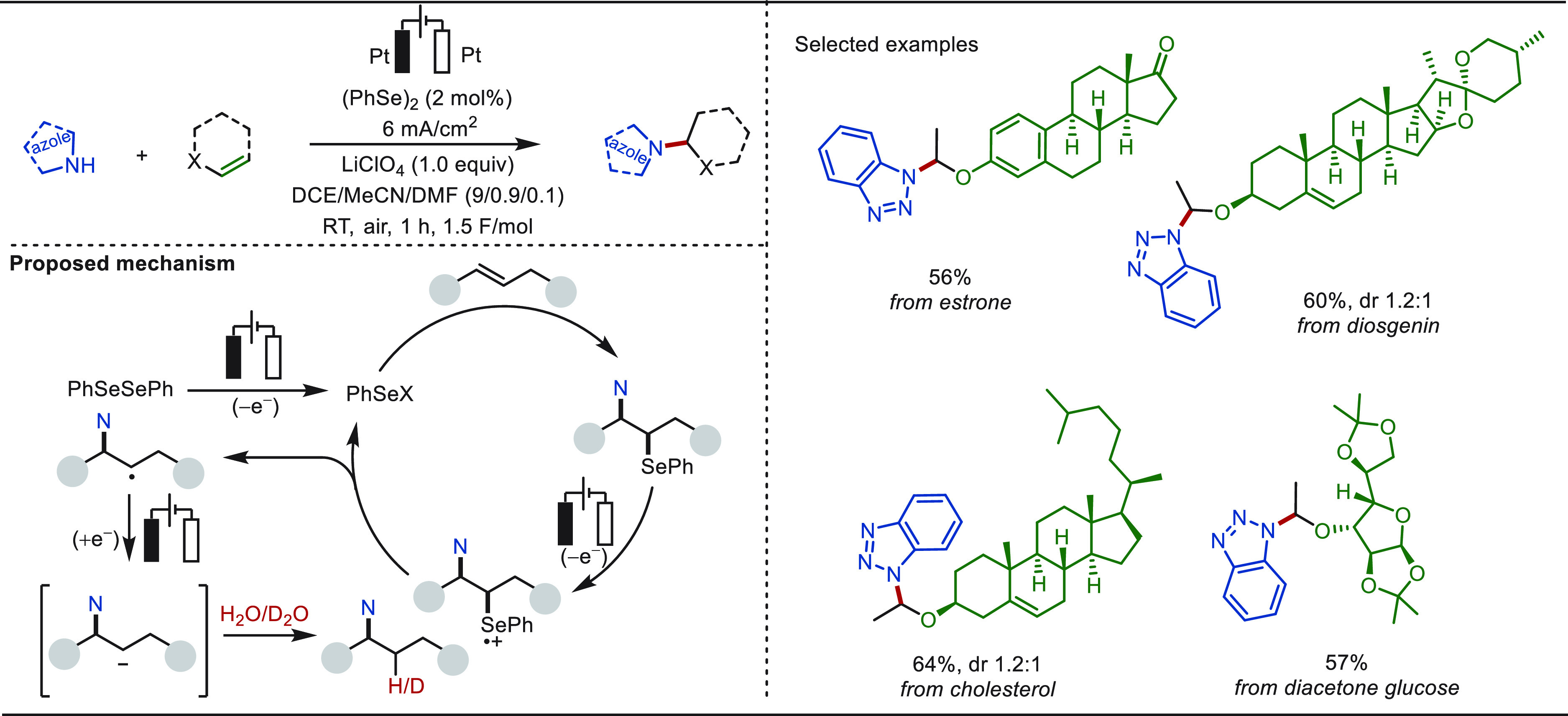
Electrochemical Selenium-Catalyzed Late-Stage Hydroazolation of Olefins

Recently, Xu and Zeng demonstrated a versatile electroseleno-catalytic hydroazolylation of olefins in the absence of external oxidants (Scheme 54).^322,323^ Electrochemical conditions acetivated the diselenide catalyst to PhSe^+^ or PhSe·, which triggered an electrophilic activation of the olefin followed by a nucleophilic addition with the azole substrate. The difunctionalized product then realized an anodic oxidation induced deselenylation generating the hydroaminated product. Deuterium labeling studies revealed the significance of the cathode in this transformation, which assisted in the formation of a carbanion. The role of the cathode was further concluded by executing the reaction in a divided cell, in which the substrate at the cathodic chamber was consumed and no product formation was observed in the anodic chamber. This electroseleno-catalyzed approach enabled the diversification of estrone, cholesterol, diosgenin, and diacetone glucose analogues with good yields.

**Scheme 55:**
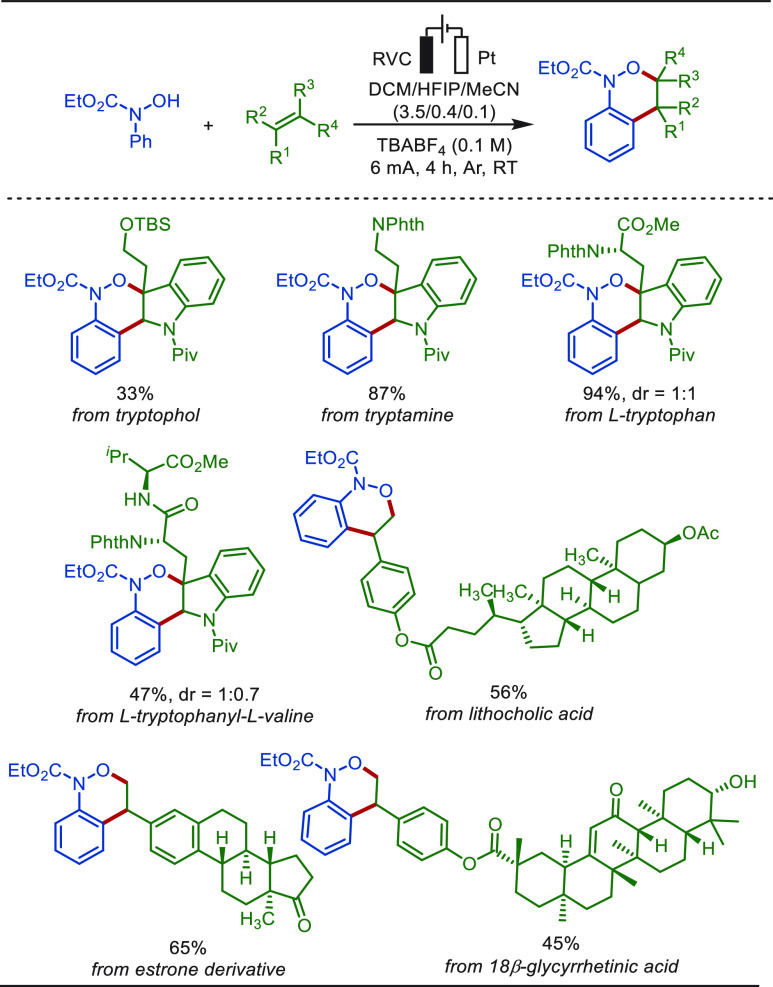
Electrochemical Late-Stage [4 + 2] Annulation of Olefins with Hydroxamic Acid

In 2021, Han reported a (4 + 2)-annulation strategy for the construction of benzo[c]-[1,2]oxazines in good yields (Scheme 55).^324^ Anodic oxidation of hydroxamic acid produced an amidoxyl radical intermediate. This intermediate reacted with the olefin substrate, constructing the oxazine core structures in decent yields. This mild, external oxidant-free approach was used to execute late-stage functionalization of tryptophol, tryptamine, tryptophan and its analogous peptides, and various steroid derivatives in high efficacy.

**Scheme 56:**
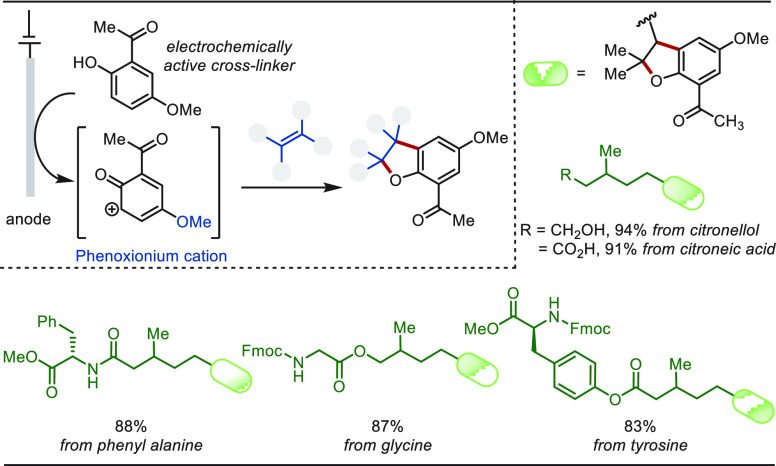
Late-Stage Electrochemical Labelling of Biologically Relevant Olefins

Anodic oxidation-based electrochemical functionalization of olefins was also transcended for a late-stage labeling of biologically relevant olefins (Scheme 56).^325^ An oxidizable phenol derivative was used as the cross-linker, which upon anodic oxidation formed a phenoxionium cation. This phenoxionium intermediate underwent a facile [3 + 2] addition with the olefin to form a fluorescent active dihydrobenzofuran moiety. The electrochemical labeling approach was able to cross-link citronellol, citroneic acid, and amino acid derivatives with high efficacy.

**Scheme 57:**
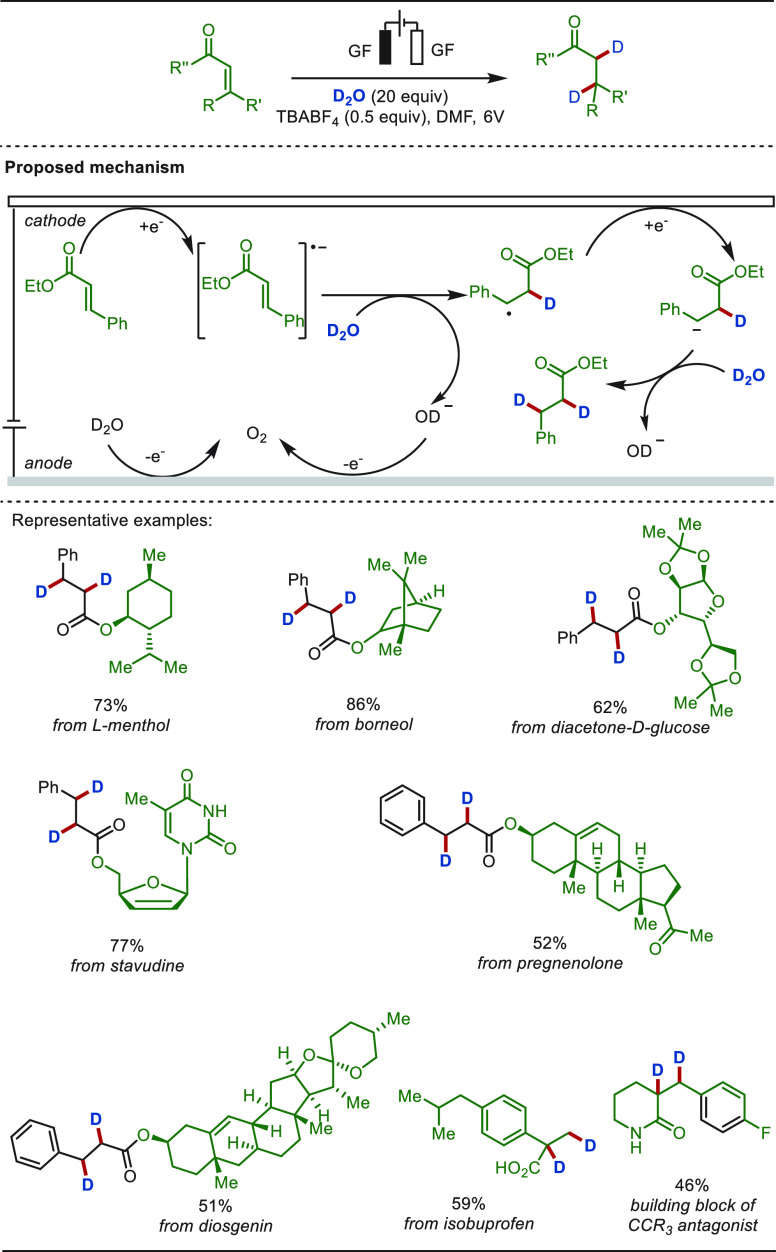
Electroreductive Late-Stage Hydrogenation of Olefins with D_2_O

Late-stage olefin functionalizations are not limited to electro-oxidative approaches. Indeed, electroreductive functionalizations of olefins are gaining significant momentum.^326^ In this context, Cheng reported selective electroreductive deuteration of α,β-unstaurated carbonyl compounds using D_2_O as the deuterium source (Scheme 57). This electroreductive approach used graphite-felt as the cathode as well as the anode and operated in the absence of an external catalyst, and thus obviated the need of stoichiometric metallic reductants unlike prior examples. An oxygen evolution was observed at the anode, confirmed using isotopically labeled water (H_2_^18^O), which regulated the need of an additional reductant along with maintaining the pH of the medium during the process. This simple and versatile deuteration method exhibited tremendous potential enabling the late-stage deuteration of a large variety of biologically relevant molecules and pharmaceuticals.

**Scheme 58:**
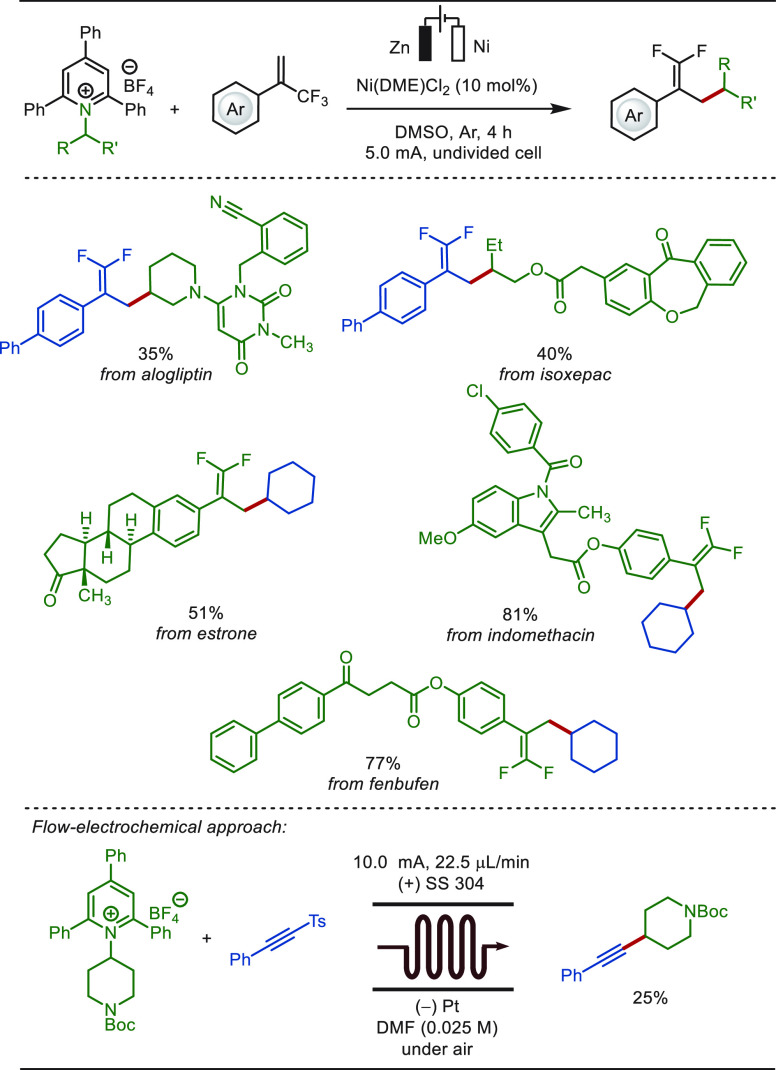
Electroreductive Late-Stage Defluorinative Alkylation of Trifluoromethylated Styrenes

In 2021, Pan disclosed a straightforward electroreductive defluorinative functionalization of trifluoromethylated styrenes (Scheme 58).^327^ Notably, straightforward synthetic routes to C–C bonds harvesting sp^3^-hybridized carbon-centered radicals have always been considered as versatile approaches in organic synthesis. The authors used easily accessible Katritzky salt as a useful source for the generation of C(sp^3^)-centered radicals. This reductive deaminative approach required a sacrificial zinc anode and obviated the need for external electrolytes. Single electron reduction of Katrizky salts generated alkyl radicals, which were intercepted by the olefin forming benzylic radical. This benzylic radical under electroreductive conditions underwent SET reduction, which facilitated the defluorination of the CF_3_ unit, generating 1,1-difluoro substituted olefins. Interestingly the method was also operative under flow-electrochemical conditions. The synthetic utility of the method was reflected by the late-stage modification of alogliptin, isopexac, estrone, indomethacin, and fenbufen analogues.

**Scheme 59:**
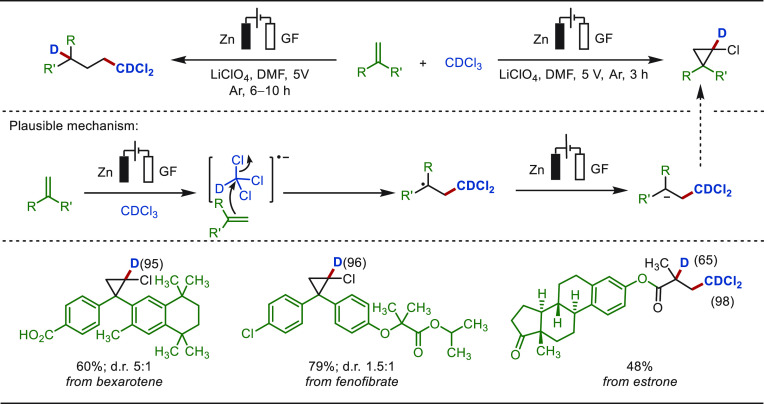
Electroreductive Late-Stage Functionalization of Olefins with Deuterochloroform

Recently, Cheng has developed an electroreductive cyclopropanation of olefins, where deuterated chloroform was used as the C1-synthon (Scheme 59).^328^ This electrochemical method employed a sacrificial Zn-anode for the SET reduction of CDCl_3_ forming a CDCl_2_ radical. The transient CDCl_2_ radical then reacted with the olefin and forged an alkyl radical, which upon eletroreduction constructed the deuterated cyclopropane derivative plausibly through the formation of a carbanion intermediate and subsequent substitution of a chloride group from the CDCl_2_ unit. Alternatively, the carbanion intermediate was also trapped using suitable proton/deuterium sources and CDCl_3_ for one-carbon elongation of terminal olefins. The current method was susceptible to afford various deuterated cyclopropane analogues with high labeling of deuterium. This cyclopropanation strategy enabled the late-stage functionalization of estrone, bexarotene, and fenofibrate analogues.

**Scheme 60:**
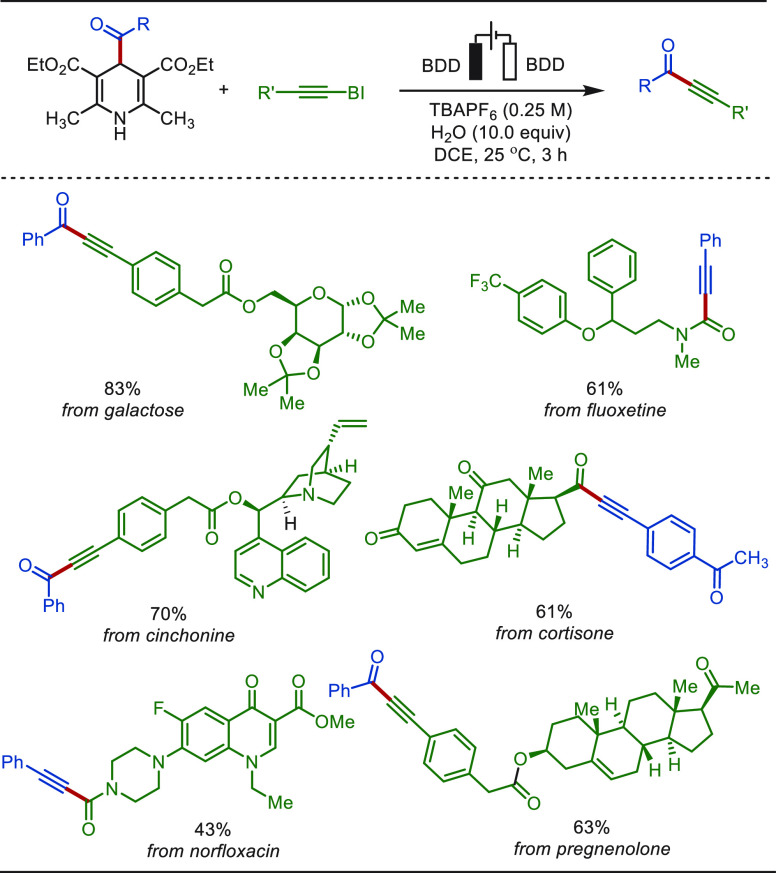
Electrochemical Synthesis of Ynones

Alkynes are present in a variety of natural products and biologically relevant molecules.^329^ Thus, late-stage alkynylation reactions and the diversification of alkynes have also remained an attractive target in the synthetic regime. Wang delineated the conversion of 4-acyl-1,4-dihydropyridines (DHPs) into ynones through an anodic oxidation-based approach (Scheme 60).^330^ This reaction proceeded through the electro-oxidation of DHPs, which then produced acyl radicals. The acyl radical intermediate was then intercepted with a hypervalent iodine derived alkynyl group transfer reagent and bestowed the ynones in good to excellent yields. Notably, boron-doped diamond (BDD) was used as the electrode for this electrochemical process. Various pharmaceuticals and biologically relevant molecules were diversified by following this approach.

### 3.2 eLSF of Organic Halides

For years, organic halides have served as convenient synthetic handles for a diverse range of functionalizations using transition-metal catalysis.^331−333^ Manipulations over these moieties result in controlled and selective functionalization processes. Notably, electrochemical conditions have also appeared as a unique transformative tool to harvest these functionalities for late-stage functionalization reactions, and several such strategies have been reported in recent years.

**Scheme 61:**
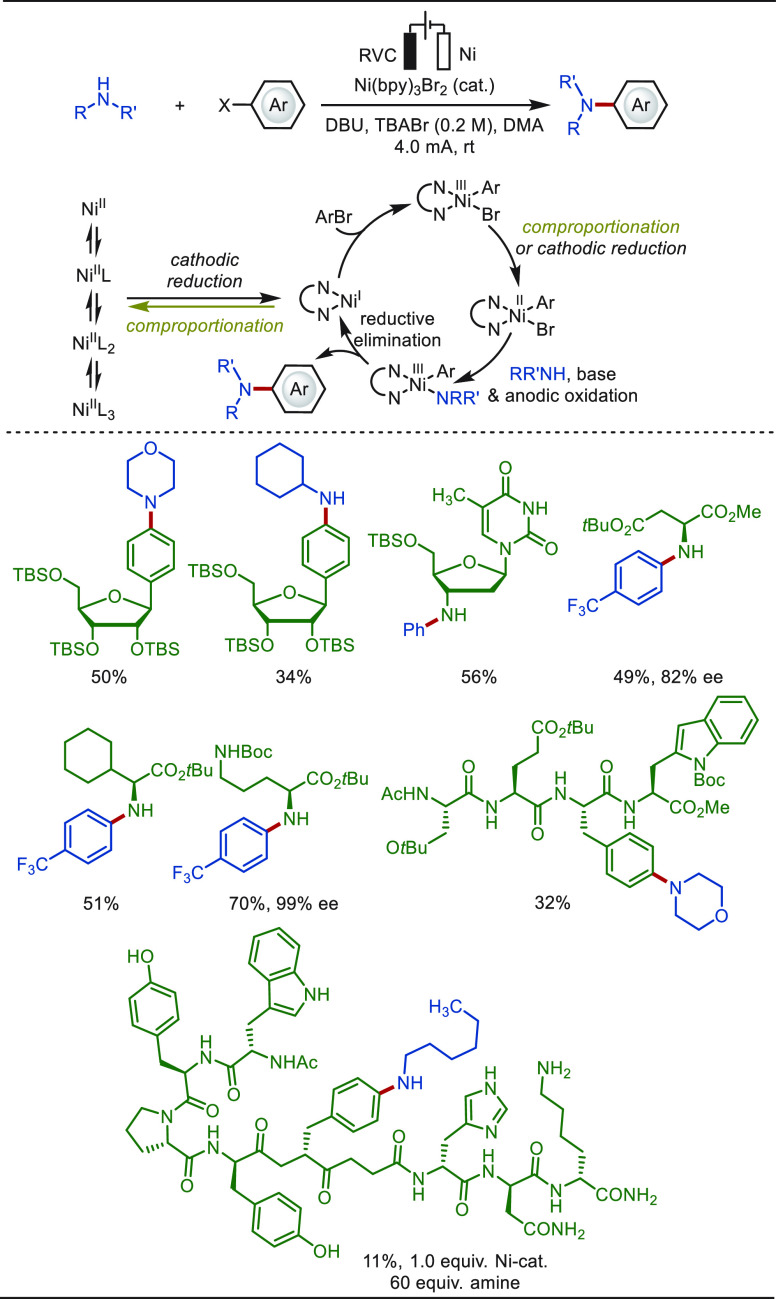
Nickel-Catalyzed Electrochemical Amination of Aryl Halides

Amines and their analogues are of considerable synthetic relevance in terms of their medicinal properties.^334^ Notably, a large variety of pharmaceuticals or biologically relevant molecules are analogues of amines. Thus, the sustainable construction of C–N bonds have gained tremendous attention in synthetic chemists’ repertoire.^256,335−338^ Despite the presence of numerous synthetic strategies to gain access to these fundamental units, general and economic approaches for late-stage amination reactions have unfortunately remained elusive. In this context, Baran reported a versatile nickel-catalyzed amination of aryl halides under electrochemical conditions (Scheme 61).^339^ This electrocatalytic protocol harvested both cathodic reduction and anodic oxidation during the catalytic cycle to enable the transformation. The proposed catalytic cycle is initiated by the cathodic reduction of nickel(II)-precatalysts to form the nickel(I) active catalyst. The oxidative insertion of the aryl bromides onto the nickel(I) catalyst followed by comproportionation or cathodic reduction of nickel(III)-species generated nickel(II)-species. The nickel(II) intermediate after ligand exchange with the amine and reductive elimination yielded the desired aminated product. The amination reaction was successfully realized with diverse amino acids, peptides, and sugar derivatives.

**Scheme 62:**
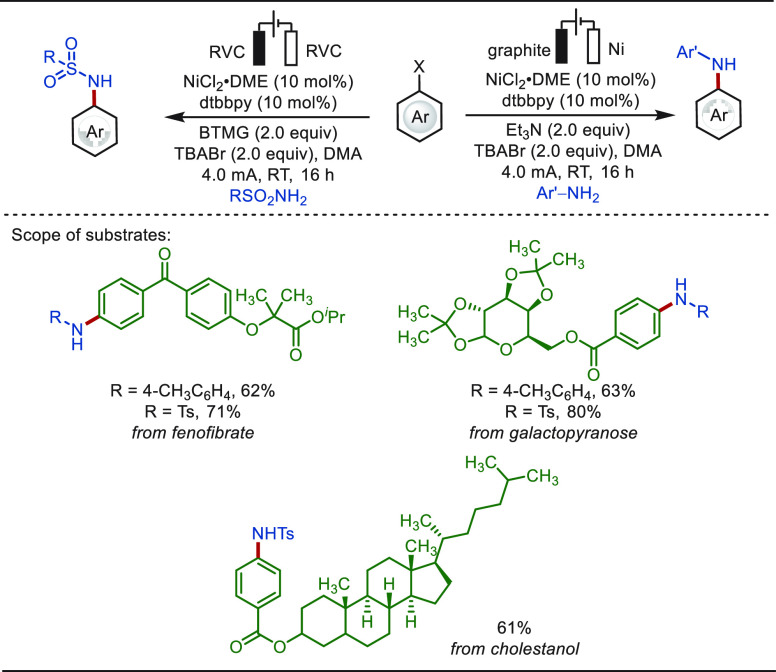
Nickel-Catalyzed Electrochemical Amination with Weak *N*-Centered Nucleophiles

In 2021, Rueping reported an electrochemical amination of aryl bromides with weak *N*-centered nucleophiles (Scheme 62).^340^ This nickel-catalyzed cross-coupling strategy was also amenable to accommodate more challenging aryl tosylates as electrophiles, harnessing anilines, sulfonamides, sulfoximines, carbamates, and imines as nucleophiles. Interestingly, the protocol was proven to be applicable for the late-stage modification of fenofibrate, galactopyranose, and cholestanol derivatives.

**Scheme 63:**
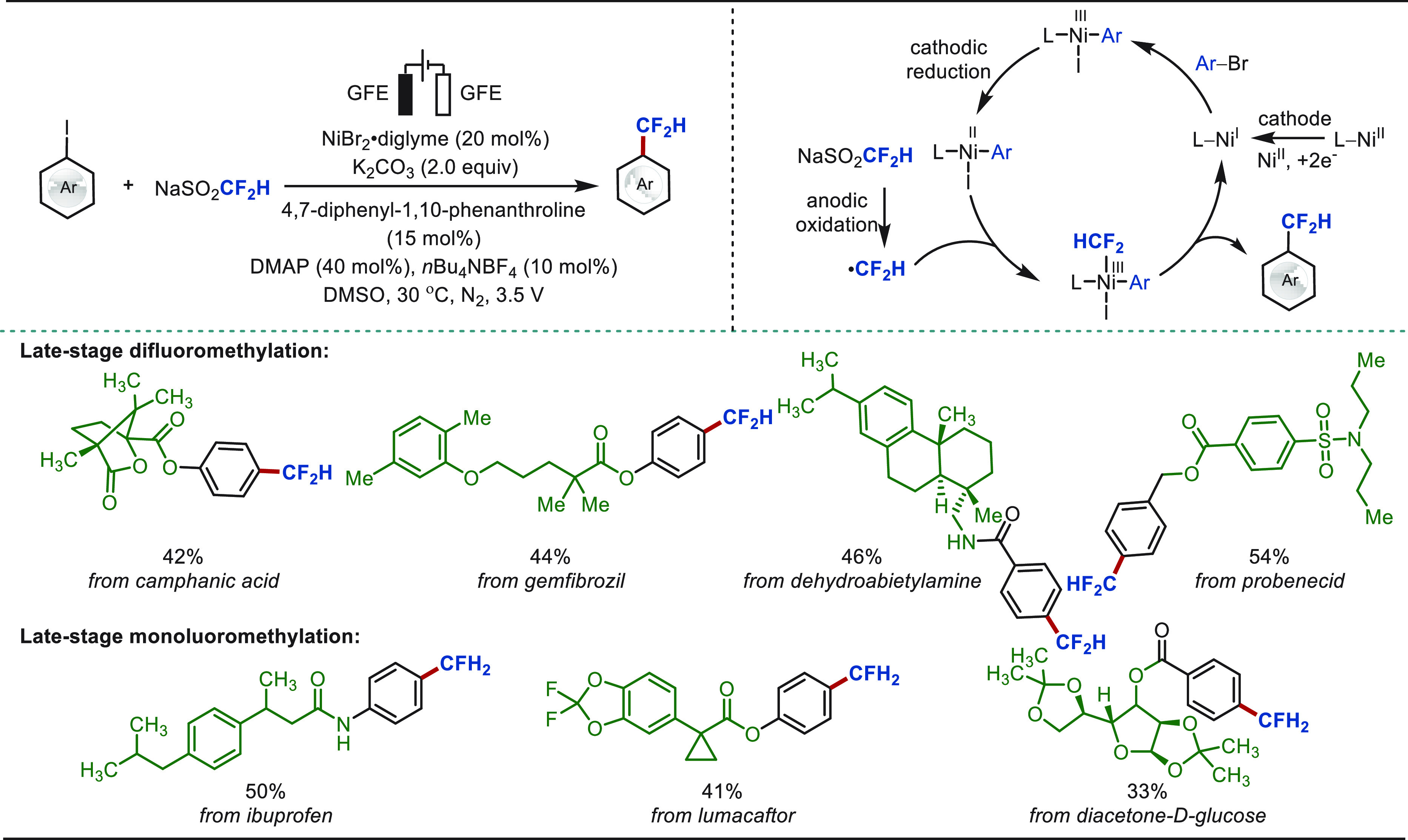
Ni-Catalyzed Late-Stage Fluoroalkylation of Aryl Iodides

Later, Wang and Zhang developed a general nickel-catalyzed fluoroalkylation strategy with aryl iodides (Scheme 63).^341^ This strategy was operative via paired electrolysis, where the sulfinate salt was oxidized at the anode, forming a fluoroalkyl radical, while the cathodic reduction allowed the low-valent nickel catalysis. This method displayed good functional group compatibility and high substrate diversity. The synthetically meaningful strategy for the incorporation of difluoromethyl and monofluoromethyl groups to arenes enabled the functionalization of a broad range of natural product analogues and biologically relevant molecules.

**Scheme 64:**
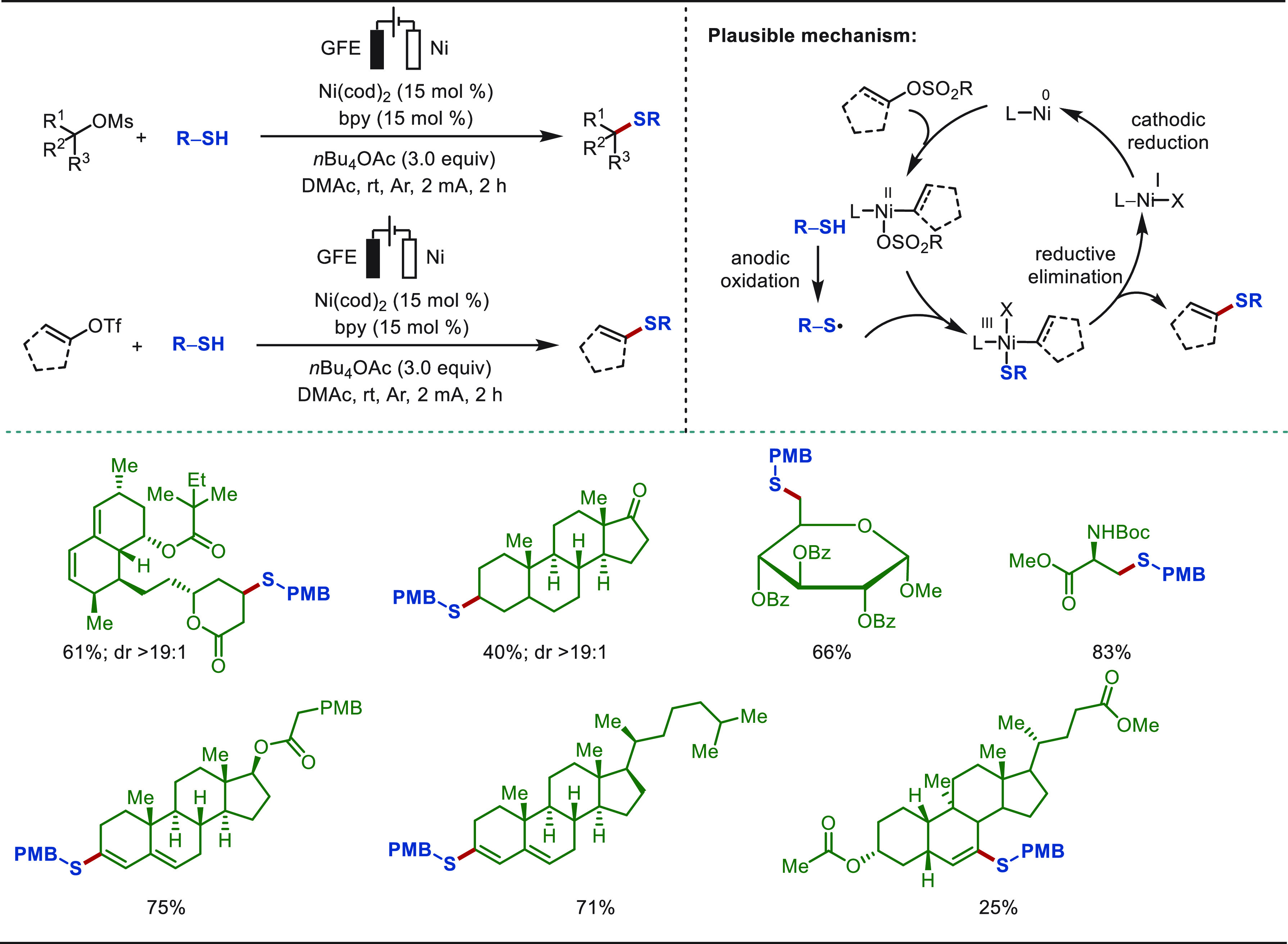
Electrochemical Nickel-Catalyzed Deoxygenative Thiolation Reactions

Electrochemical nickel-catalyzed functionalization strategies were not limited to organic halides. Indeed, the corresponding pseudohalides also served well as useful substrates for such transformations.^342−344^ In 2021, Wang and co-workers reported one such example, in which electrochemical nickel-catalysis was employed for deoxygenative thiolation of ketones (Scheme 64).^345^ In both of the cases the deoxygenation was facilitated through an initial activation of alcohols and ketones converting them to alkyl mesylate and vinyl triflate analogues, respectively. These reactive pseudohalide functionalities efficiently participated in the nickel-catalyzed thiolation reactions, generating the corresponding thioethers in high yields. Both approaches showed excellent functional group compatibility and enabled the late-stage diversification of a glucide derivative and various steroid analogues.

**Scheme 65:**
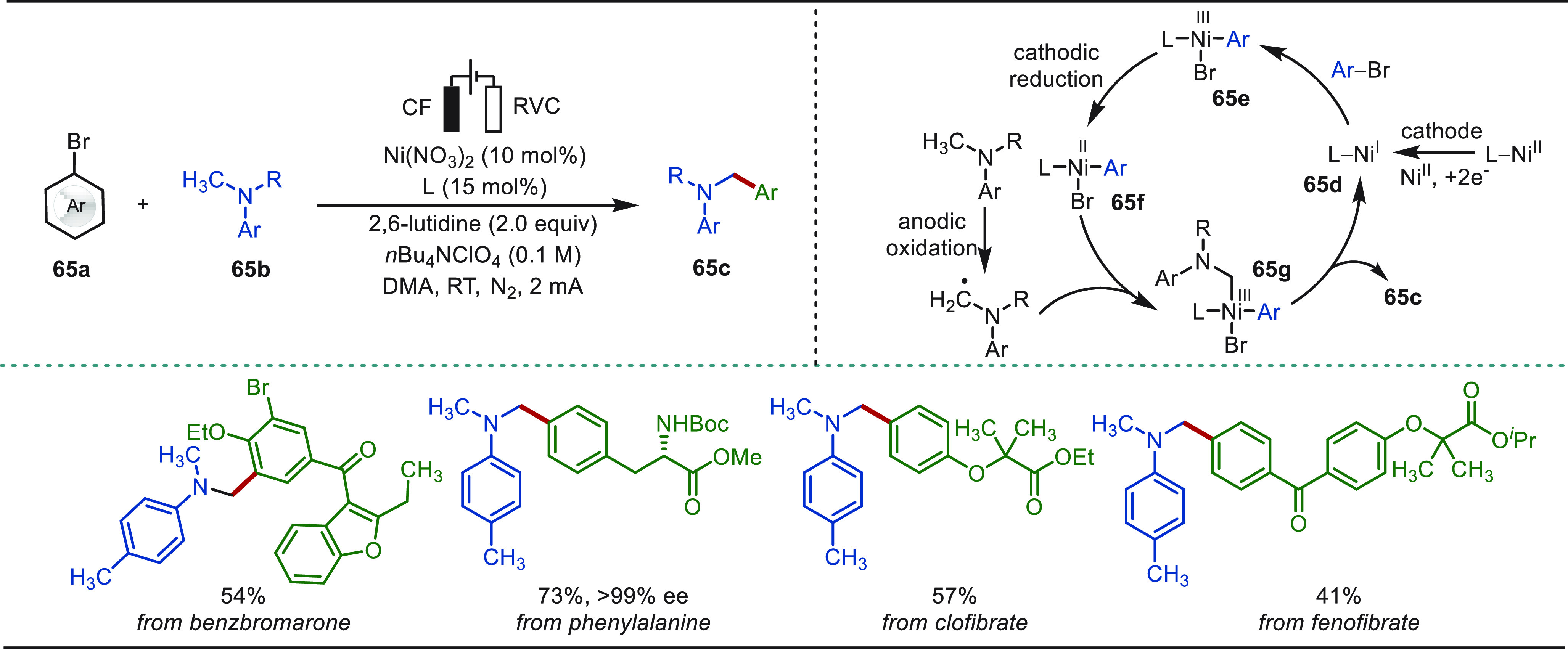
Nickel-Catalyzed Late-Stage Aminomethylation of Aryl Bromides

In 2021, Ye and Li reported an electrochemical nickel-catalyzed aminomethylation reaction of aryl bromides (Scheme 65).^346^ The reaction was proposed to proceed through a cathodic reduction of the nickel(II)-precatalyst, which upon oxidative addition to the aryl bromide gives intermediate **65e**. Intermediate **65e** after another cathodic reduction was intercepted by the aminomethyl radical, generated through anodic oxidation of the corresponding tertiary amine, producing intermediate **65g**. Reductive elimination of **65g** gave the desired aminomethylated product. This strategy was particularly powerful for the late-stage derivatization of benzobromarone, phenylalanine, clofibrate, and fenofibrate analogues.

**Scheme 66:**
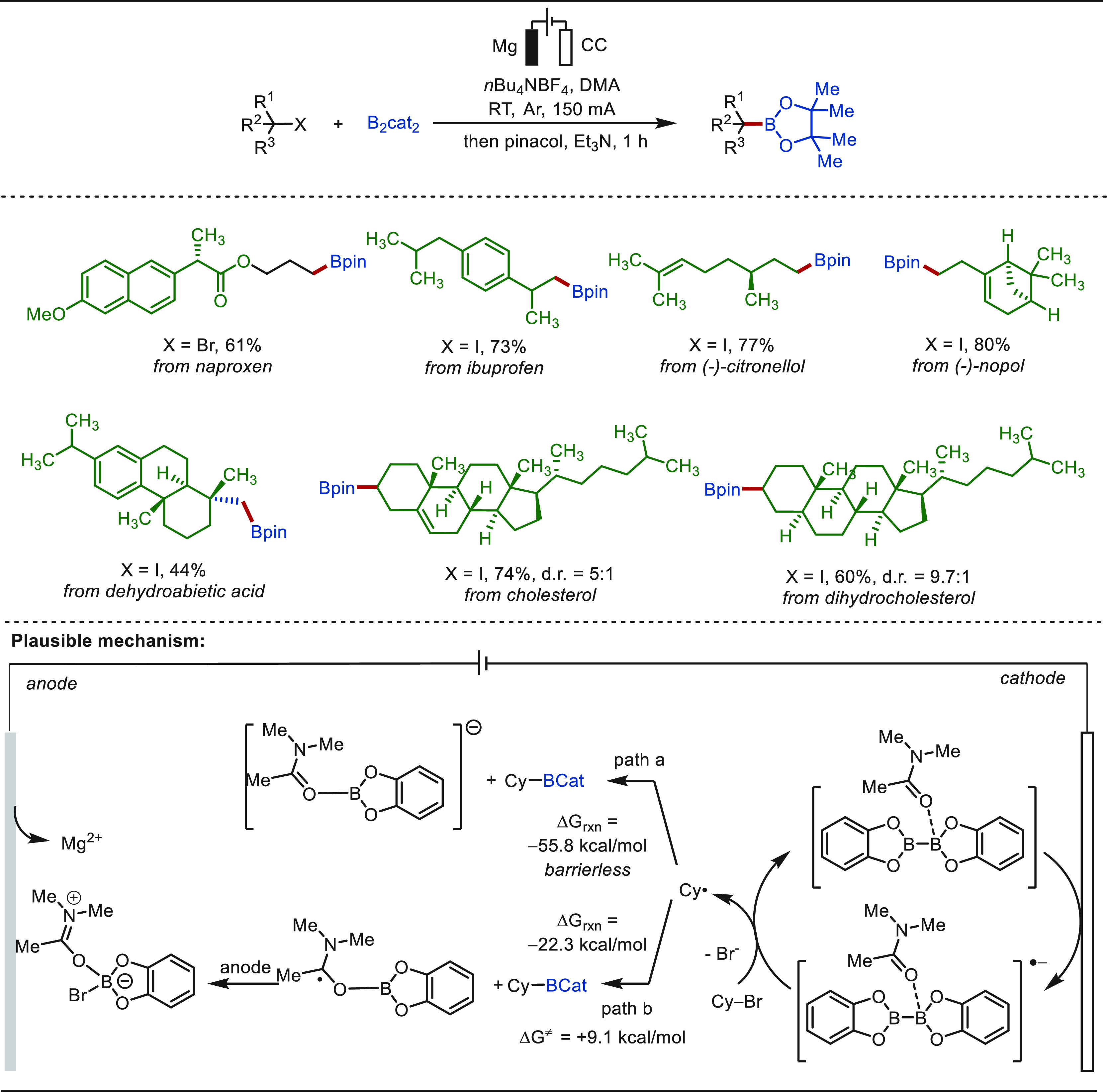
Electrochemical Late-Stage Borylation of Alkyl Halides

In 2021, a practical borylation was developed by Qi and Lu under electroreductive conditions (Scheme 66).^347^ The reduction of organic halides was induced with the aid of a sacrificial magnesium-anode, generating an alkyl radical, which was then intercepted with the diborane to construct the borylated product. The protocol operated under a high current (∼150 mA) with alkyl chlorides, bromides, and iodides. The efficiency of the approach was demonstrated through efficient late-stage borylation of natural products and drug analogues. Notably, the borylating agent served both as a boron source and a mediator controlling the reactivity of the process. The DMA stabilized B_2_cat_2_ mediated the single-electron reduction of the alkyl halide generating the alkyl radical, which then realized a barrierless radical–radical cross-coupling with B_2_cat_2_. Detailed DFT studies rendered the possibility of path b unlikely to be operative, with an activation energy barrier of 9.1 kcal/mol.

**Scheme 67:**
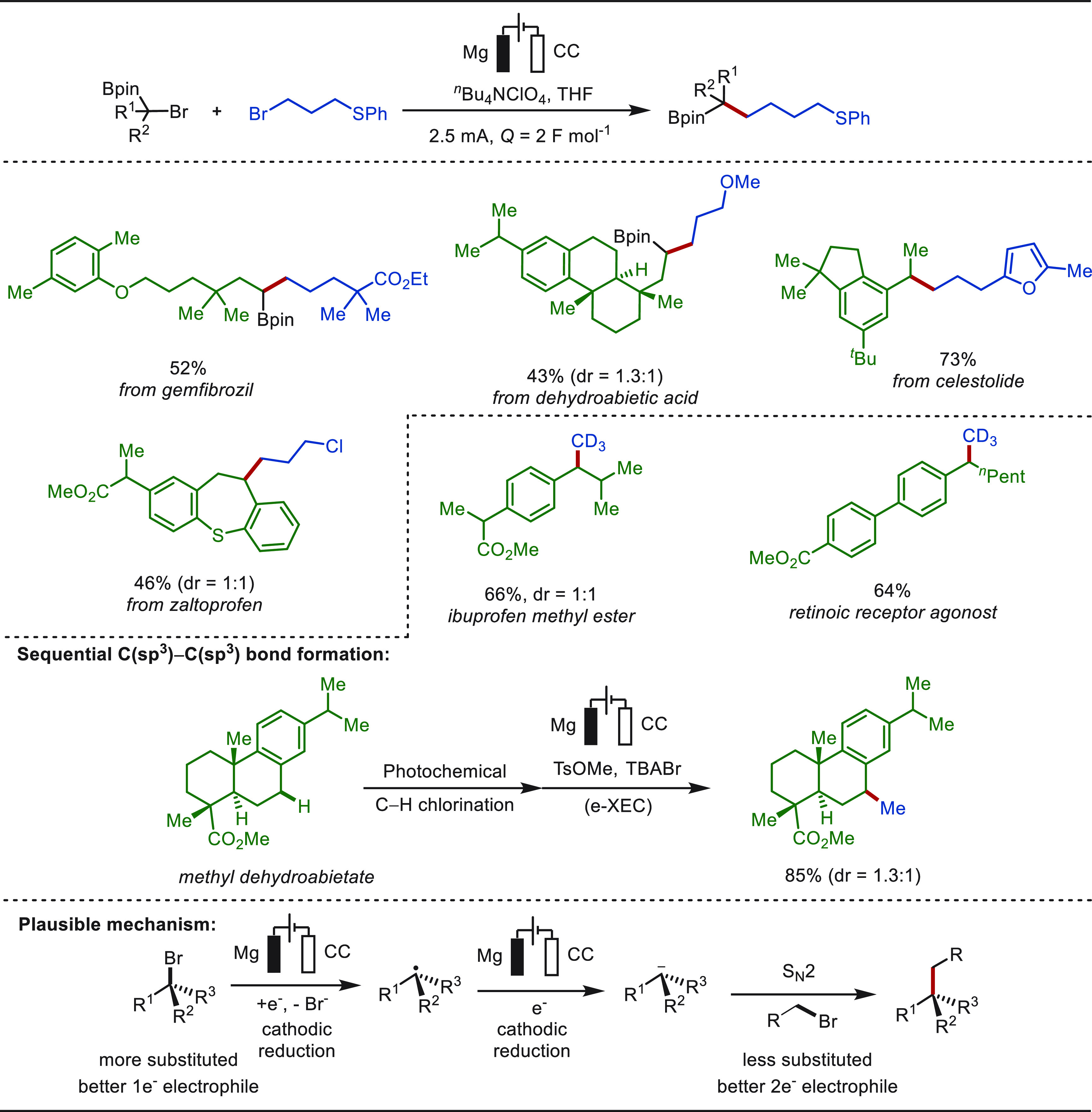
Electrochemically Driven Late-Stage C(sp^3^)–C(sp^3^) Bond Formation

The presence of C(sp^3^)-rich organic molecules may improve efficacy for the drug-candidates in clinical trials.^348^ Thus, constant effort has been devoted to devising methods for selective formation of C(sp^3^)–C(sp^3^) bonds. In 2022, Lin and co-workers developed an electroreductive strategy for the construction of C(sp^3^)–C(sp^3^) bonds harvesting easily accessible alkyl halides as the alkyl source (Scheme 67).^349^ A selective cathodic reduction of the more substituted alkyl halide to the corresponding carbanion governed a preferential substitution of comparatively less substituted alkyl halide, forging the C(sp^3^)–C(sp^3^) bond with high precision. Altering the transition-metal-catalyzed approach with the direct electrolysis of alkyl halides avoided the typical β-hydride elimination pathway, offering a modular selective C(sp^3^)–C(sp^3^) bond formation. A sacrificial Mg-anode effectuated this transformation and the mild reaction conditions enabled the late-stage modification of complex organic molecules and drug derivatives. Further, a sequential photochemical chlorination of benzylic C–H bond followed by electroreductive methylation of methyl dehydroabietate exhibited the broad synthetic utility of this electrochemically driven cross-electrophile coupling (e-XEC). Interestingly, this sequential electrochemical alkylation process was also successful for the late-stage deuteromethylation of ibuprofen and retionic acid receptor agonist.

**Scheme 68:**
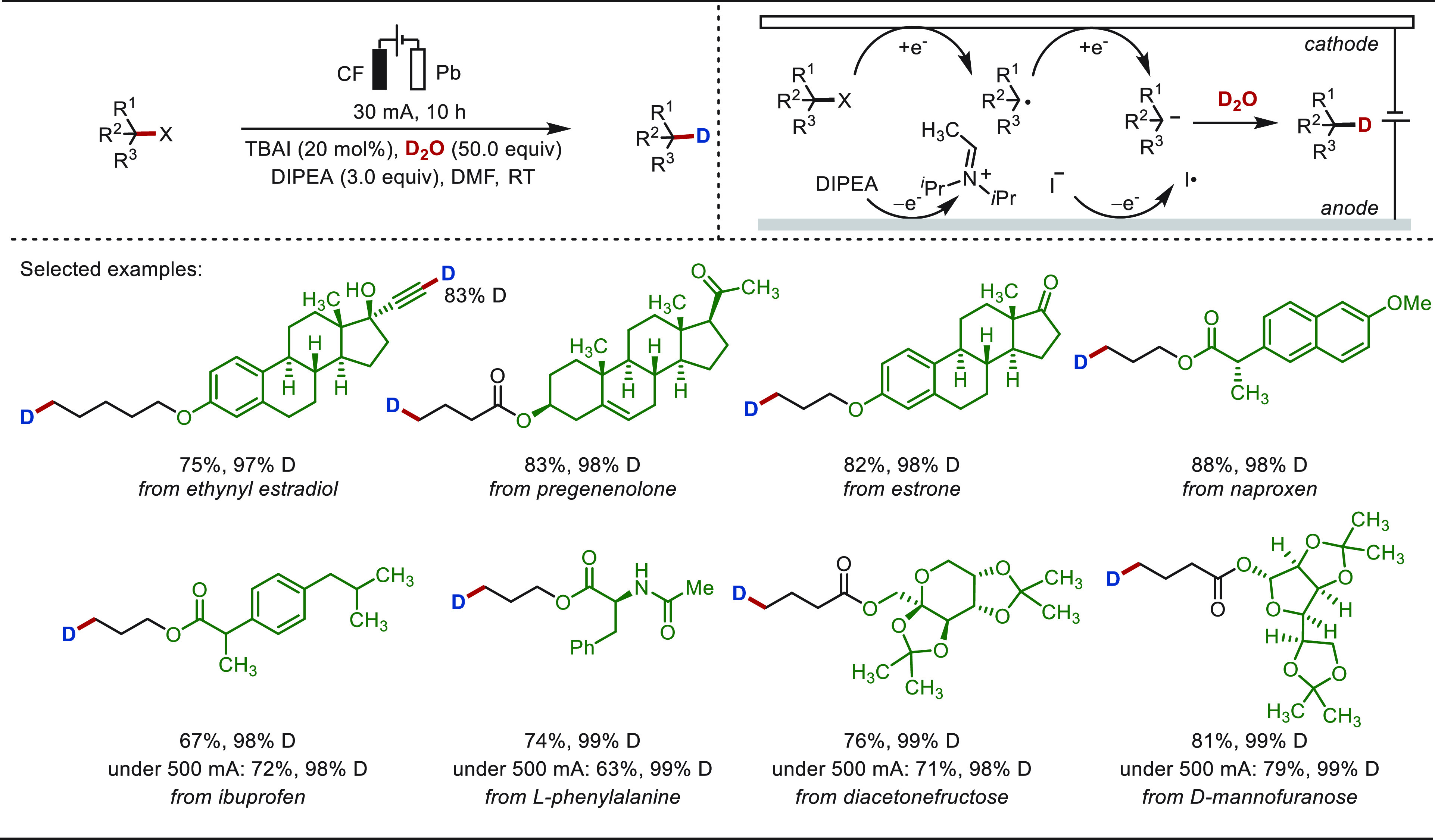
Electrochemical Late-Stage Deuteration of Alkyl Halides

Reductive deuteration of organic halides^331,350−352^ constitutes a promising method to prepare deuterated molecules, which are widely used in pharmaceutical research.^353−355^ In 2022, Qiu described an interesting example of late-stage deuteration of organic halides, where high deuterium incorporation (up to 99%) in the product was achieved using simple D_2_O as the deuterium source (Scheme 68).^356^ The plausible reaction mechanism involved a 2-fold cathodic reduction of the organic halide forming a carbanion intermediate, which is quenched with the D_2_O present in the medium. This protocol was adopted for the deuterium labeling of various pharmaceuticals and their intermediates along with other complex substrates. The strategy also functioned well under 500 mA current with high selectivity, which intimated the applicability in industrial application. Under related conditions, the electrochemical reductive deuteration of aryl halides and benzylic chlorides was achieved by Lei^357^ and Lin,^358^ respectively.

**Scheme 69:**
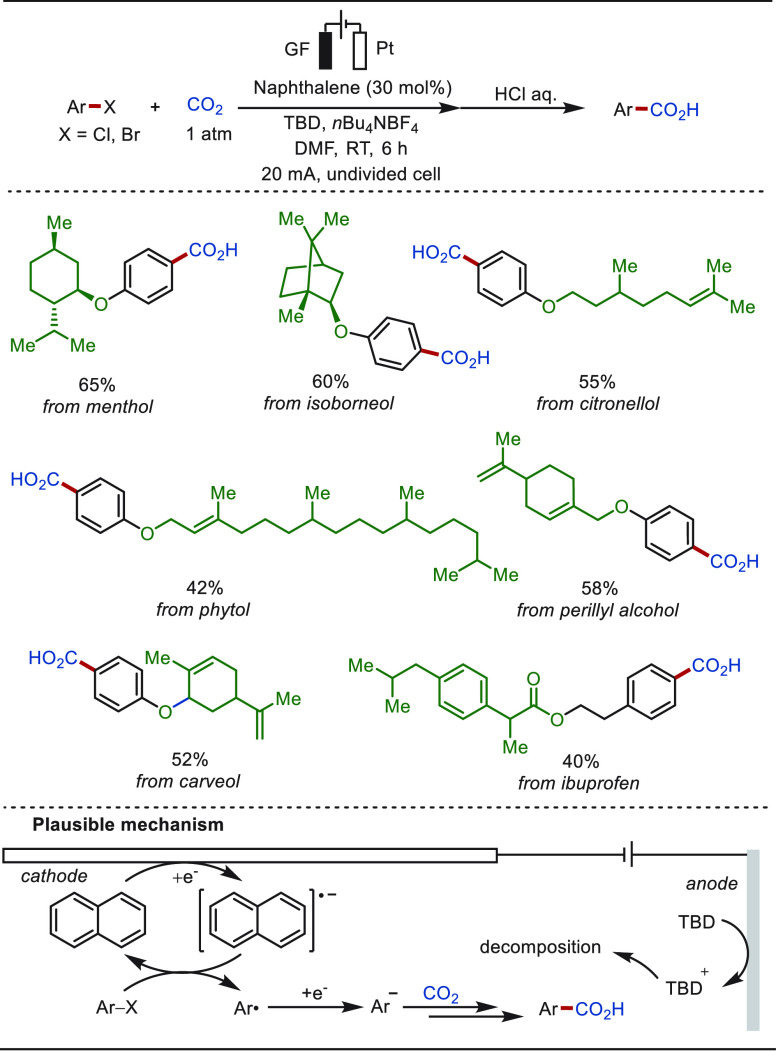
Electrochemical Carboxylation of Aryl Halides

Recently, a metal-free electroreductive carboxylation of aryl halides was devised by Qiu (Scheme 69).^143,359^ This electroreducitve protocol functioned by utilizing naphthalene as a catalytic mediator without any sacrificial anode material. The naphthalene mediator under the catalytic conditions formed a strong reductant naphthalene anion radical, which reduced the aryl halide generating aryl radical. Consequently, the aryl radical was quenched with CO_2_ delivering the carboxylic acid. This simple dehalogenative strategy was effective for the late-stage carboxylation of several natural products, drugs, and bioactive compounds.

### 3.3 eLSF of Organic Carboxylic Acid and Derivatives

Carboxylic acids are one of the most versatile feedstocks in modern organic synthesis.^360−362^ This functionality is also highly abundant in natural products, bioactive molecules, and pharmaceuticals.^363^ Thus, electrochemical decarboxylative strategies, since its inception from popular Kolbe electrolysis, have flourished significantly for the late-stage functionalization of valuable organic molecules.^50,364^

**Scheme 70:**
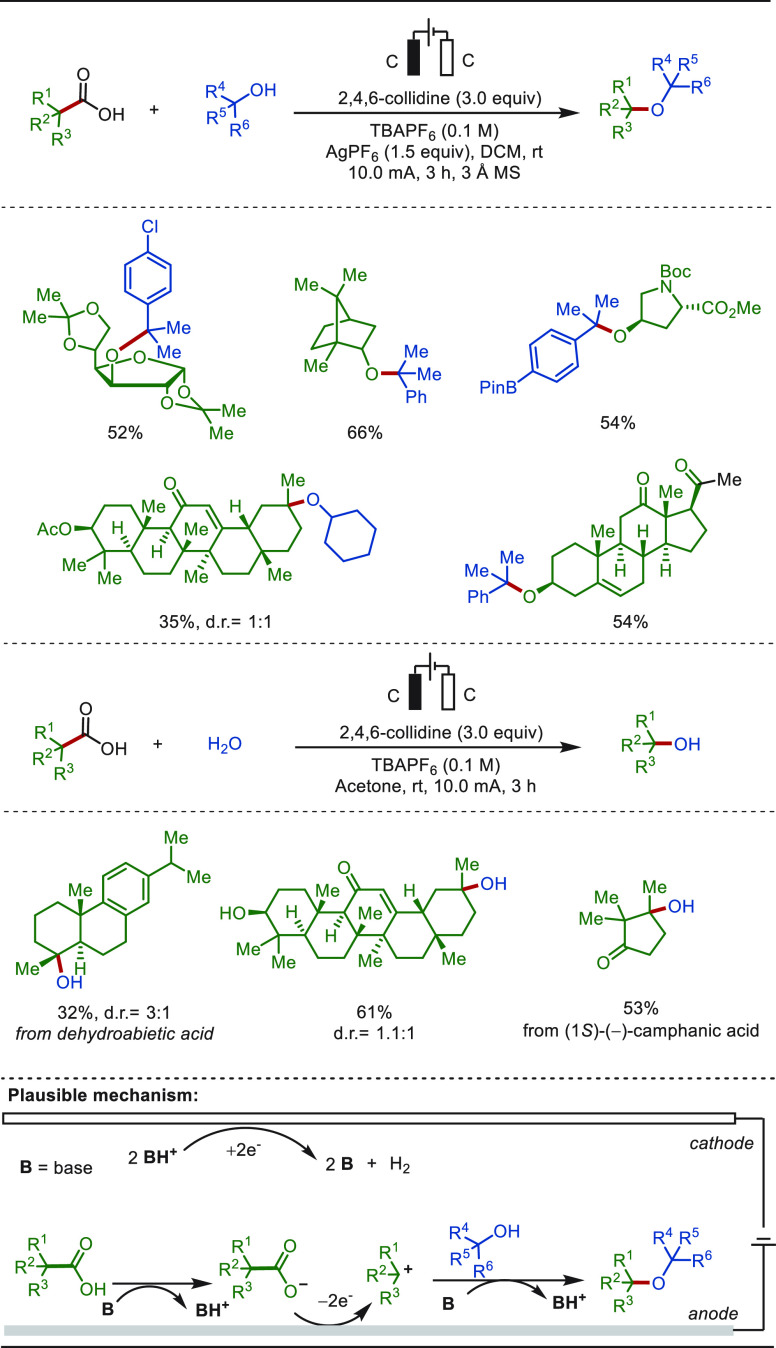
Decarboxylative Synthesis of Sterically Hindered Ethers

In 2019, Baran reported an electrochemical decarboxylative synthesis of hindered aliphatic dialkylethers harnessing electrogenerated carbocation intermediates (Scheme 70).^365^ Sterically hindered dialkylethers, though highly coveted motifs owing to their medicinal importance, are challenging to access through conventional synthetic approaches.^366^ However, the trapping of the carbocation intermediate, generated from the direct electrochemical oxidation of easily accessible carboxylic acids, with alcohols furnished these valuable organic molecules in a straightforward manner. This method operated under the most user-friendly and mild conditions, tolerating diverse common functional groups in the substrates. This electro-oxidative reaction was successful in accomplishing late-stage functionalization of a large variety of complex and biologically relevant organic molecules. Further, water was also an effective nucleophile under these conditions to deliver late-stage decarboxylative hydroxylation of complex organic molecules.

**Scheme 71:**
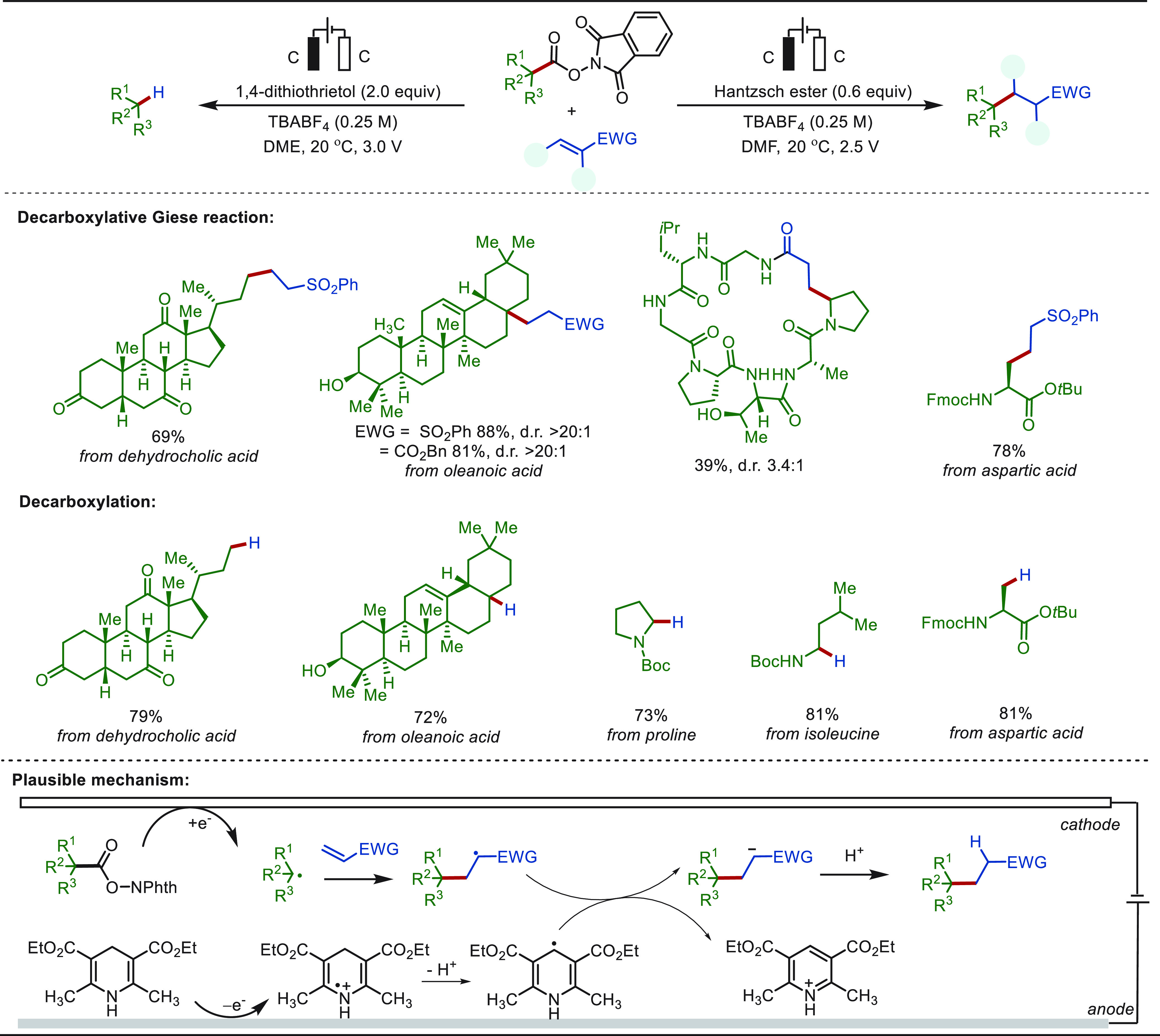
Electrochemical Decarboxylative Functionalization of Bioactive Carboxylic Acids

In this context, the Wang group reported a protodecarboxylation as well as a decarboxylative Giese reaction of aliphatic carboxylic acids, which were efficient for the functionalization of various amino acids and natural products (Scheme 71).^367^ The decarboxylation proceeded through the single-electron reduction of the redox-active ester (RAE), which upon decarboxylation generated the alkyl radical. The alkyl radical was then trapped with the activated olefin to produce the desired product. In the absence of the olefin, decarboxylated products were obtained.

**Scheme 72:**
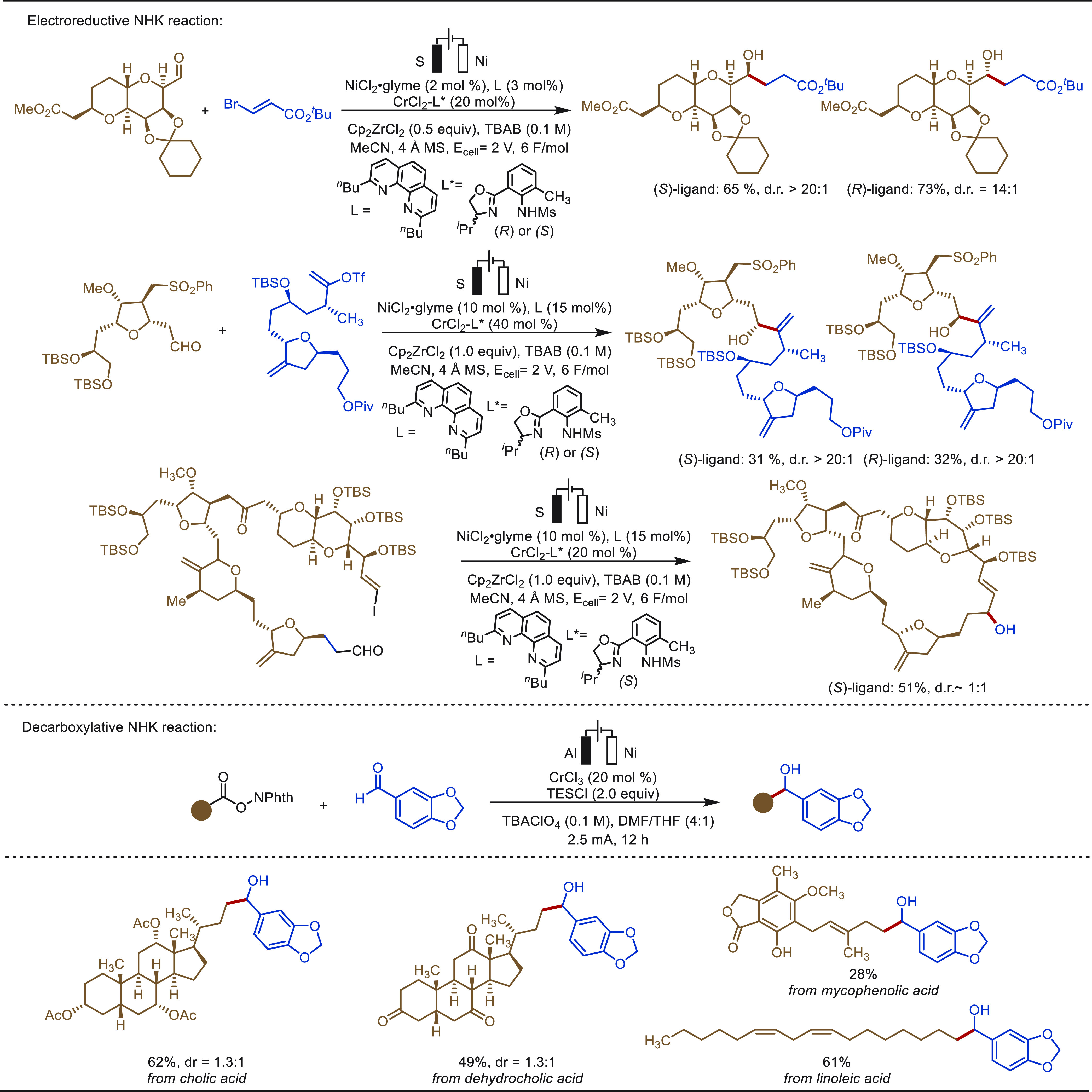
Late-Stage Modification through Electrochemical Nozaki–Hiyama–Kishi Reaction

In 2021, Baran reported an electroreductive Nozaki–Hiyama–Kishi (NHK) reaction, a popular strategy often applied in natural product synthesis, generating complex allylic alcohols from easily accessible vinyl halides and aldehydes (Scheme 72).^368^ This approach used catalytic amounts of chromium salts, avoiding stoichiometric metallic reductants. While similar transformations had previously been achieved by Grigg, Tanaka, and Périchon/Durandetti, these approaches mainly suffered in terms of the broader synthetic applicability and complex reaction settings.^369−372^

**Scheme 73:**
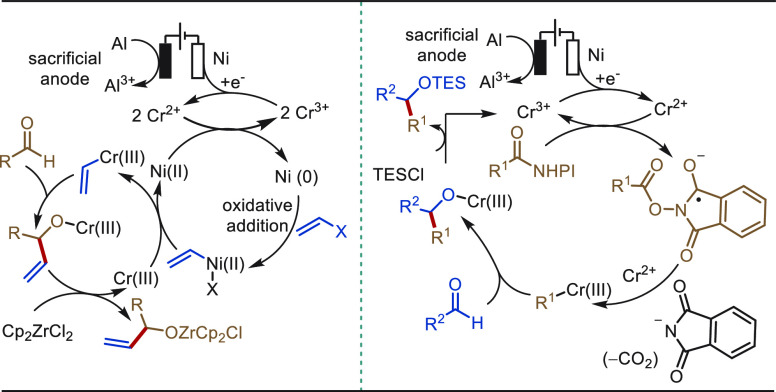
Plausible Mechanism for Electrochemical NHK Reaction

The combination of nickel- and chromium-salts under constant potential enabled this transformation, which was applied for the synthesis of complex chiral allylic alcohols including the synthesis of a Halaven intermediate. This electroreductive approach was also effective to harness noncannonical redox-active esters as useful substrates for the generation of secondary alcohols in a straightforward manner. Mechanistic investigations revealed that the reaction rate of the e-NHK reaction was faster compared to classical NHK reactions (Scheme 73). Spectro-electrochemical studies justified the presence of chromium(III)-species in the catalytic process, while the presence of Ni(II)-species significantly influenced the electron transfer process to chromium(III). The e-NHK reaction commenced with the cathodic reduction of chromium(III)-salt to chromium(II), which reduced the nickel(II) cocatalyst to nickel(0). This nickel(0) species underwent a facile oxidative addition with the vinyl halide to form respective intermediate, which upon transmetalation with chromium(III) followed by 1,2-addition with the aldehyde released the allyl alcohol analogue. The chromium(III)-catalyst was regenerated with another transmetalation with Cp_2_ZrCl_2_. The decarboxylative variant of this transformation also followed a similar mechanistic pathway, in which the Cr(II)-species facilitated the single-electron reduction of the redox-active ester to form an alkyl-Cr(III) species. This intermediate bestowed the product after 1,2-addition and silylation of the alkoxy-Cr(III) intermediate.

**Scheme 74:**
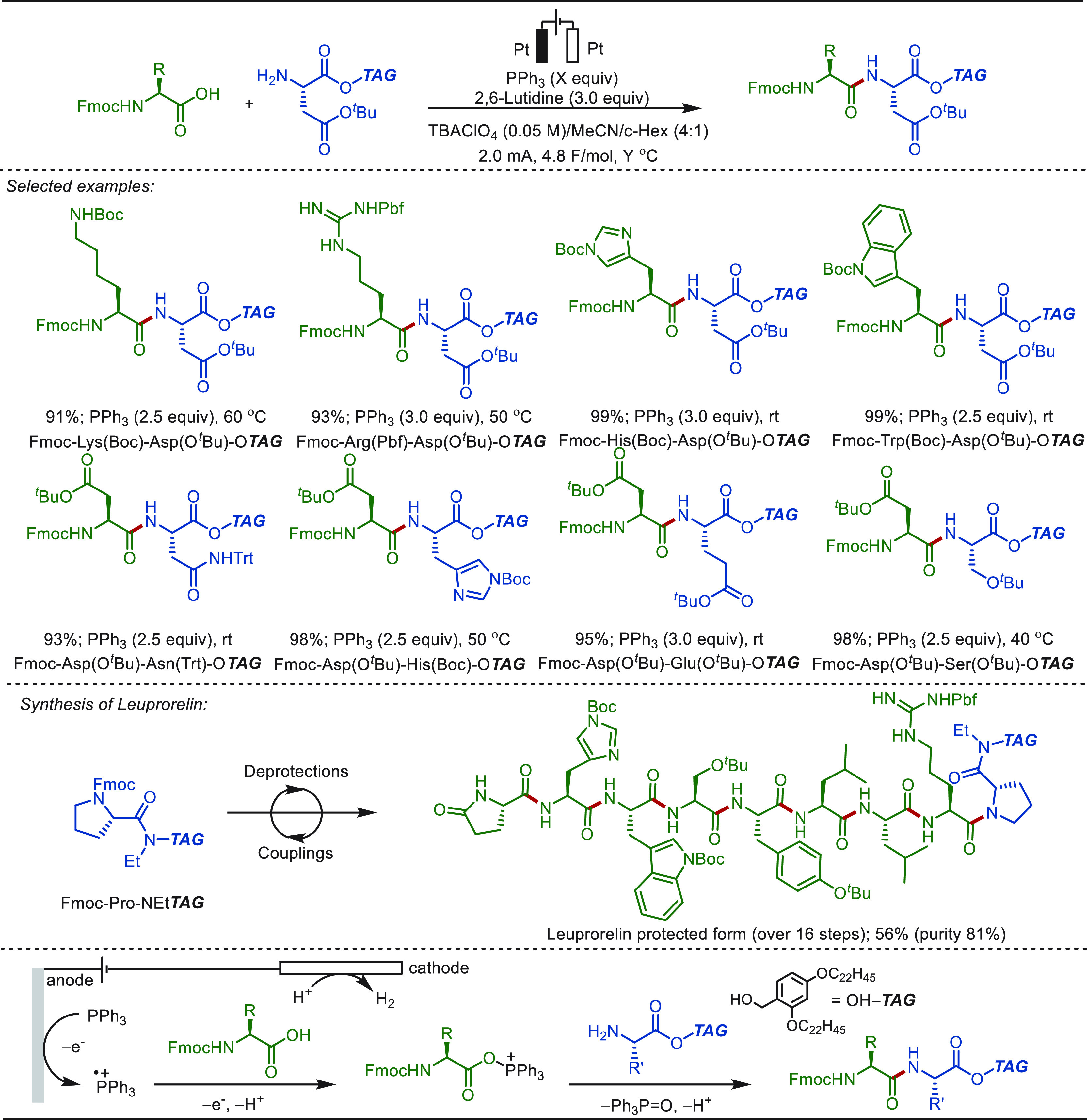
Electrochemical Synthesis of Peptides

In 2021, Chiba described a green biphasic peptide synthesis protocol using electro-oxidative conditions (Scheme 74).^373^ Stoichiometric amounts of PPh_3_ were used as the additive, which under electrochemical oxidation formed a triphenylphosphine radical cation. This intermediate activated the terminal carboxylic acid of the amino acid, and then a nucleophilic displacement reaction at the carbonyl center with another amino acid constructed a peptide bond along with a reusable Ph_3_PO byproduct. This process proved viable to access a library of small peptides and successfully implemented for the synthesis of active pharmaceutical ingredient (API), leuprorelin without using traditional expensive peptide synthesis reagents.

**Scheme 75:**
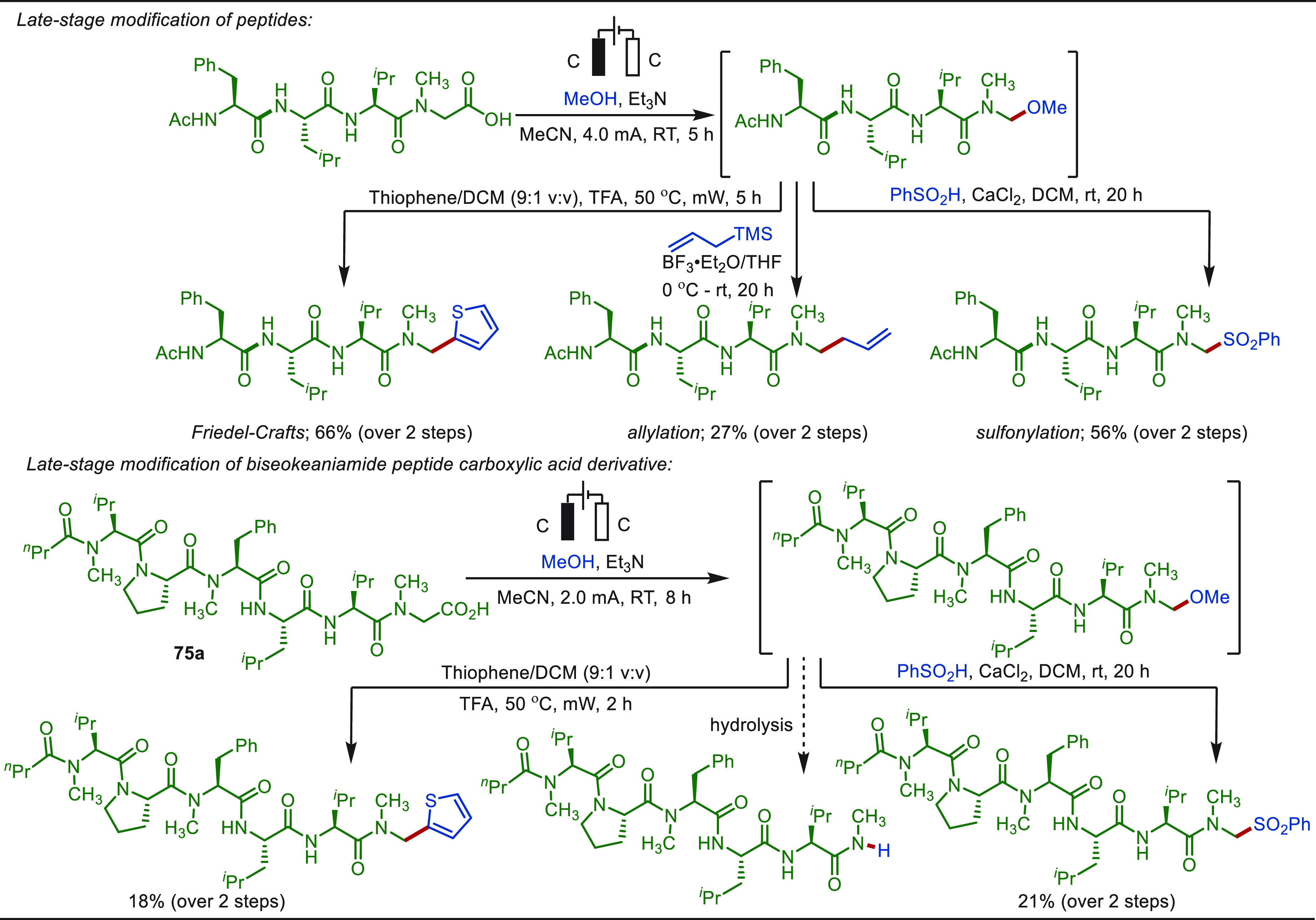
Electrochemical Decarboxylative Functionalization of Peptides

In 2020, Malins developed an electrochemical decarboxylation-nucleophilic addition approach (Scheme 75).^374^ First the oxidative decarboxylation at the *C*-terminal led to the formation of *N*,*O*-acetal intermediates, which after the treatment of nucleophiles under acidic conditions forged the functionalized product. The synthetic utility of this method was mirrored by the divergent synthesis of various bioactive peptides, including biseokeaniamide analogues **75a**.

**Scheme 76:**
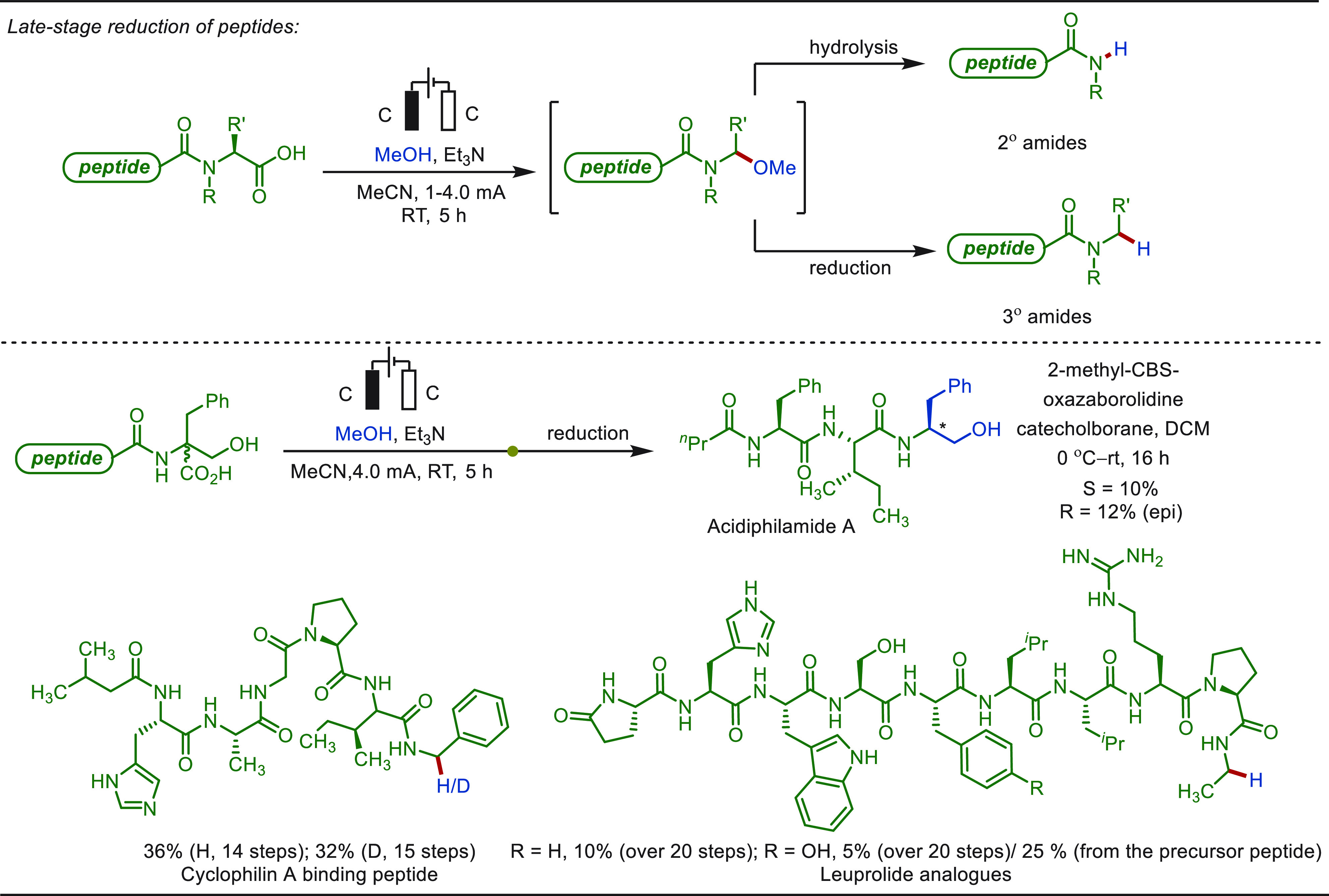
Electrochemical Decarboxylative Modification of *C*-Terminal Peptides

This oxidative decarboxylative approach was further extended by Malins for the synthesis of designer *C*-terminal peptides (Scheme 76).^375^ Decarboxylation followed by a reduction of the *N*,*O*-acetal under acidic conditions led to the formation of the desired peptides in decent yields. The innate reactivity of the *C*-terminal carboxylate analogue was exploited under robust conditions, where a large variety of proteinogenic functionalities were tolerated and the designer peptides were obtained without epimerization. Utilizing this electrochemical strategy, natural product acidiphilamide A as well as an anti-HIV peptide and the cancer therapeutic leuprolide were synthesized.

**Scheme 77:**
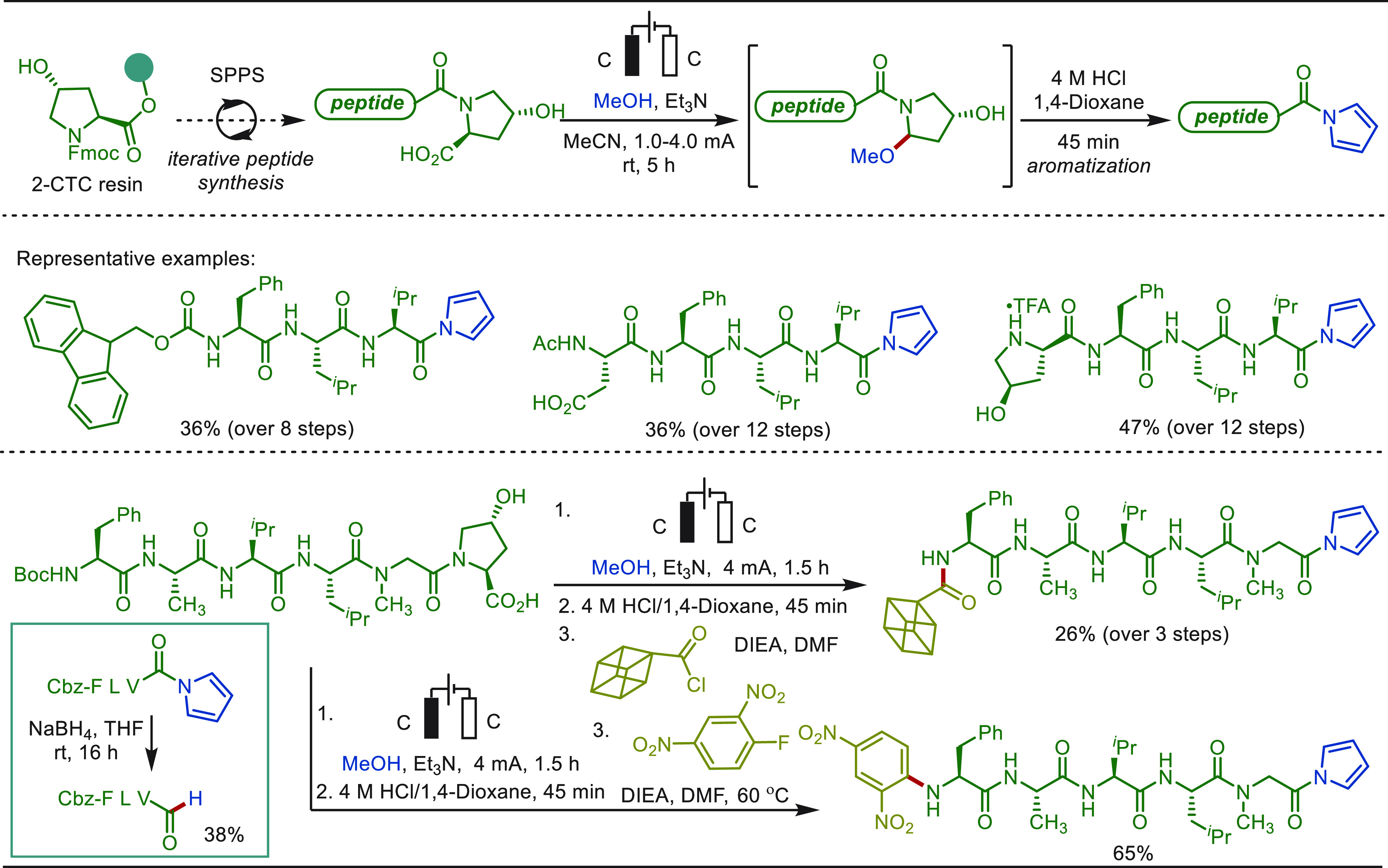
Electrochemical Decarboxylative Aromatization of *C*-Terminal Hydroxyproline Containing Peptides

Recently, Malins has further extended the decarboxylative functionalization strategy combining with an acid promoted aromatization for late-stage modification of *C*-terminal hydroxyproline containing peptides to access a library of *C*-terminal *N*-acylpyrrole derivatives (Scheme 77).^376^ Identical to prior examples, this strategy was also operationally simple, compatible with various common protecting groups in the peptide-chain, and useful to incorporate bioisosteres and peptide labels. Respective aldehyde analogues were amenable through the reduction of the *C*-terminal *N*-acylpyrrole containing peptide derivatives.

**Scheme 78:**
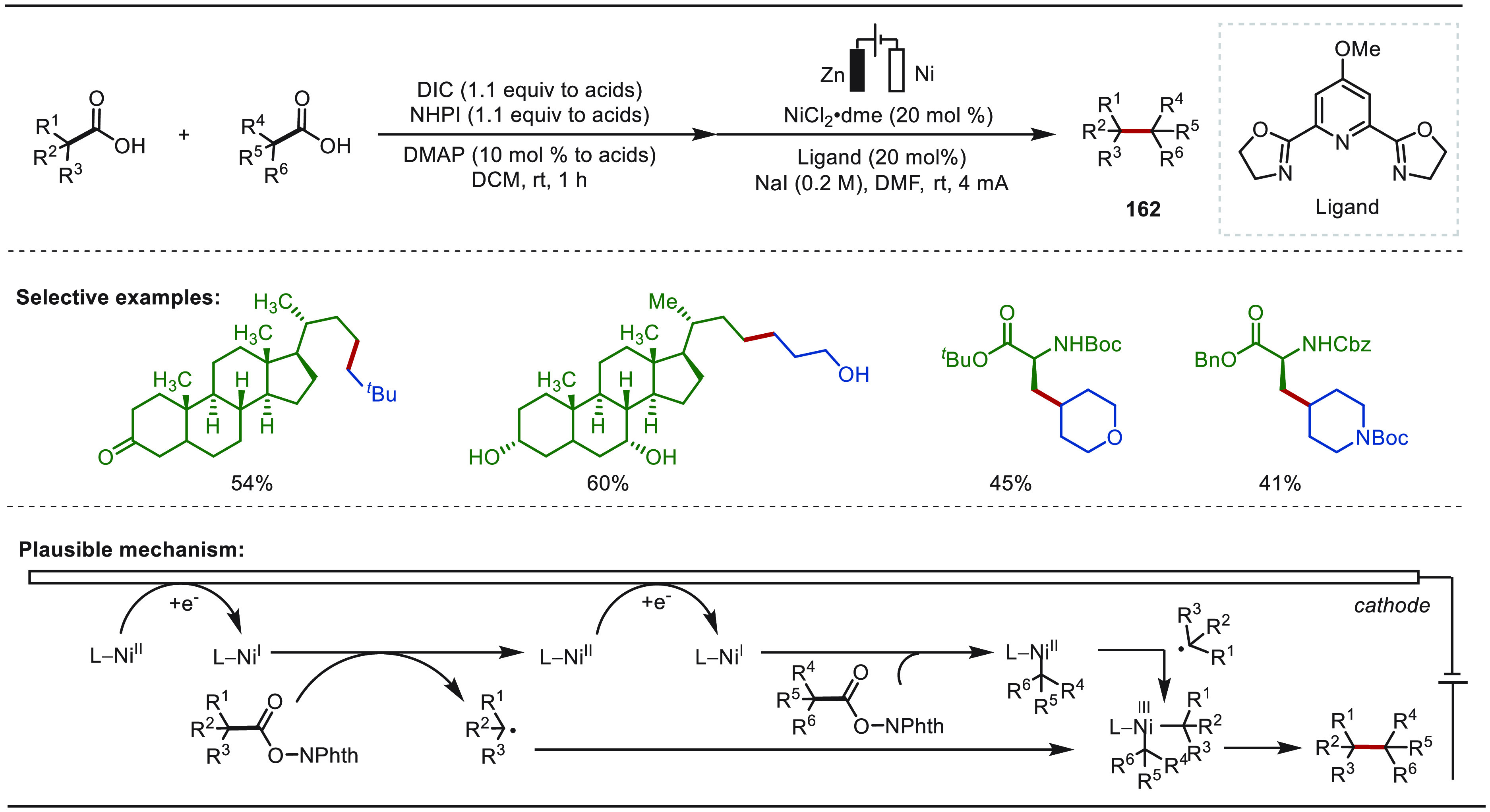
Electroreductive Nickel-Catalyzed Decarboxylative C(sp^3^)–C(sp^3^) Bond Formation

Baran further contributed in this area depicting a nickel-catalyzed decarboxylative C(sp^3^)–C(sp^3^) bond formation reaction (Scheme 78).^377^ The synthetic method used redox-active *N*-hydroxyphthalimide protected carboxylic acid ester derivatives as the alkyl source, where two distinct alkyl groups were stitched together by the nickel-catalyst. This electroreductive process effectively combined primary, secondary, and even tertiary alkyl radicals for selective C(sp^3^)–C(sp^3^) bond formation. This simple single-step process tolerated diverse common functional groups exploiting widely available carboxylic acid derivatives as useful synthons. Notably, several complex natural products and biologically relevant molecules were easily manipulated utilizing this nickel-catalyzed strategy.

### 3.4 eLSF of Alcohols and Derivatives

**Scheme 79:**
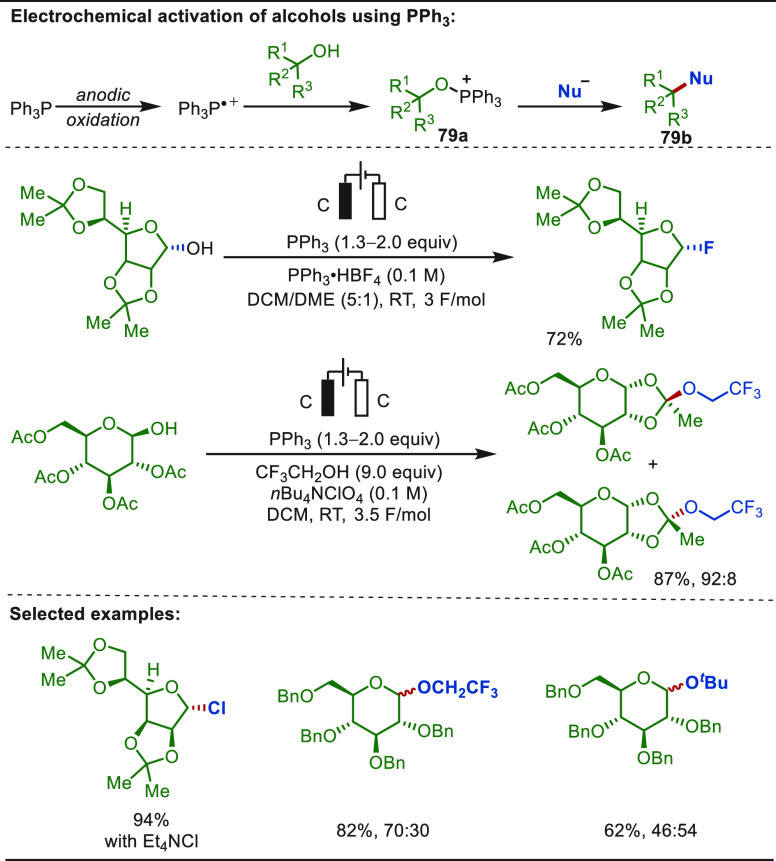
Late-Stage Dehydroxylative Halogenation and Alkoxylation

**Scheme 80:**
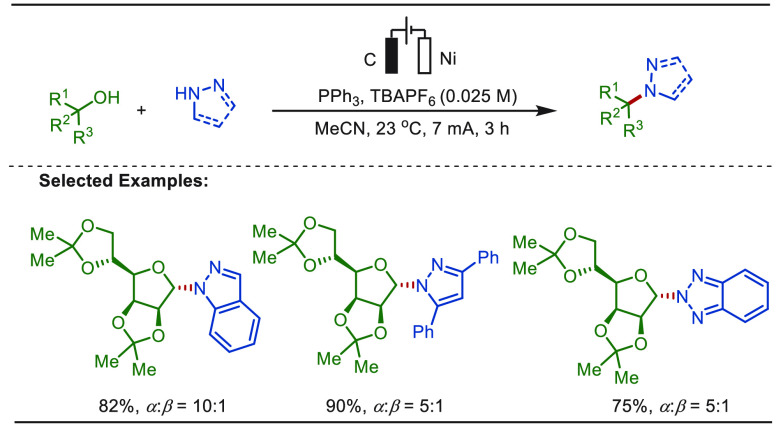
Electrochemical Late-Stage Dehydroxylative Azolation

Alcohols, and analogues thereof, have always remained in the general focus of synthetic chemists, as they are prevalent motifs in a multitude of natural products, pharmaceuticals, and biologically relevant molecules.^378^ Thus, the synthesis and derivatization of this prevalent functionality attract significant attention. However, direct functionalization over these synthetic handles is cumbersome due to the high C–O bond strength, which can be accomplished through the activation of the C–O bonds by other means. In this domain, electrochemical approaches have served well, providing some practical transformative alternatives, which are also extended for late-stage functionalization reactions.^379^ An early example by Ohmori and co-workers highlighted that anodic oxidation of triphenylphosphine could execute an efficient C–O bond activation through alkoxy phosphonium salt formation.^380−382^ Oxidation of PPh_3_ generated a triphenylphosphine radical cation, which was intercepted by the alcohol to form the alkoxy phosphonium salt intermediate **79a**, nucleophilic substitution of which bestowed functionalized product **79b** (Scheme 79). The method was pertinent in combination with a large variety of nucleophiles. This versatile process was applied for the late-stage deoxygenative functionalization of glycosides with fluoride and chloride nucleophiles.^383^ Recently, Wang and Tian harnessed this electro-oxidative approach for a deoxygenative C–N bond formation reaction, which thereby allowed for the glycosylation of azoles in straightforward manner under mild conditions (Scheme 80).^384^

**Scheme 81:**
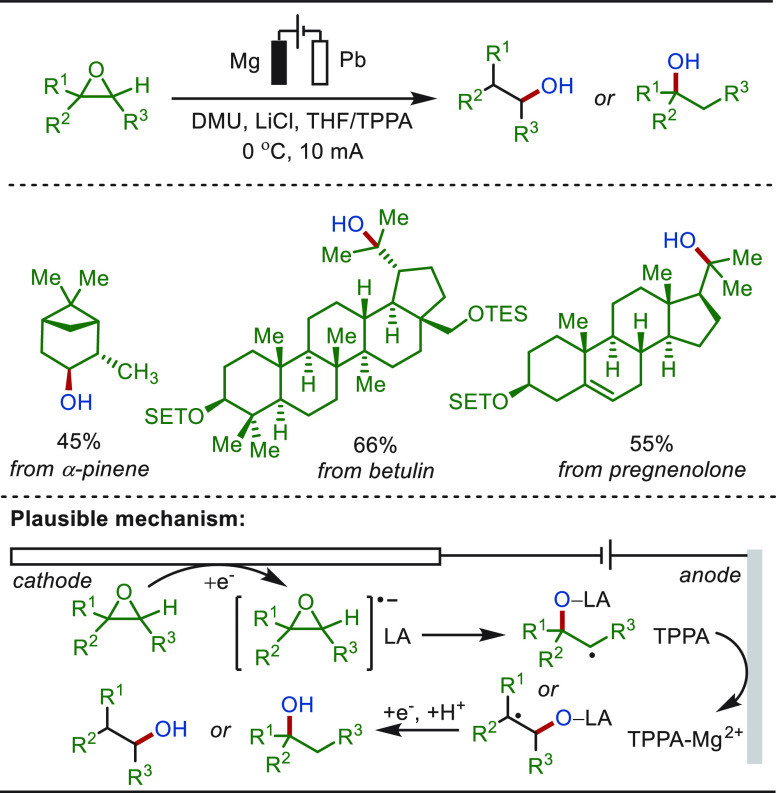
Electrochemical Transition-Metal Free Reduction of Epoxides

The epoxide functionality is a highly reactive synthetic handle for the diversification of organic molecules.^385^ While nucleophilic displacement reactions are one of the most common approaches in modifying epoxides, recently electroreductive approaches are also gaining significant momentum for the late-stage functionalization of epoxides of biologically relevant molecules. In 2022, Lu and Qi described an electrochemical transition-metal free reduction of epoxide to generate primary, secondary, and tertiary alcohols (Scheme 81).^386^ This approach delivered both regioisomeric ring-opening products, where the thermodynamic stability of the benzylic radical was decisive for aryl epoxides and the alkyl epoxides realized ring-opening following a kinetic manifold. The electroreductive method was able to deliver the corresponding alcohols of natural products α-pinene, betulin, and pregnenolone. A plausible mechanism of this reaction involved the single-electron reduction of the epoxide in the presence of a Lewis acid followed by protonation.

**Scheme 82:**
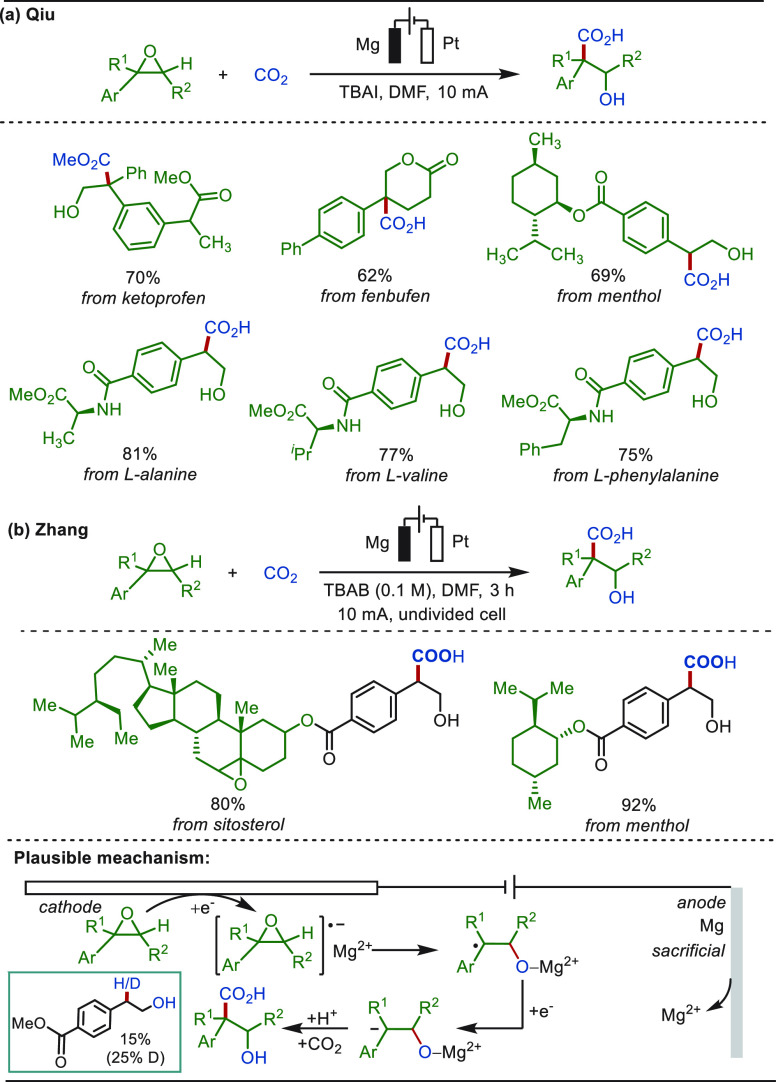
Electrochemical Transition-Metal Free Carboxylation of Epoxides

Recently, efficient electroreductive protocols to access β-hydroxycaboxylic acids from readily available aryl epoxides were reported by Qiu^387^ and Zhang,^388^ independently (Scheme 82). The strategy used a sacrificial magnesium anode to promote the cathodic reduction of epoxide, forming a benzylic radical, which upon another cathodic reduction generated a carbanion intermediate. The carbanion intermediate was quenched by CO_2_ constructing the desired product. The formation of carbanion intermediate was confirmed by a deuterium labeling study. This method was able to functionalize aryl epoxides derived from drug molecules and amino acids giving access to corresponding β-hydroxycaboxylic acids in good to excellent yields.

### 3.5 Late-Stage Reduction of Arenes and Ketones

**Scheme 83:**
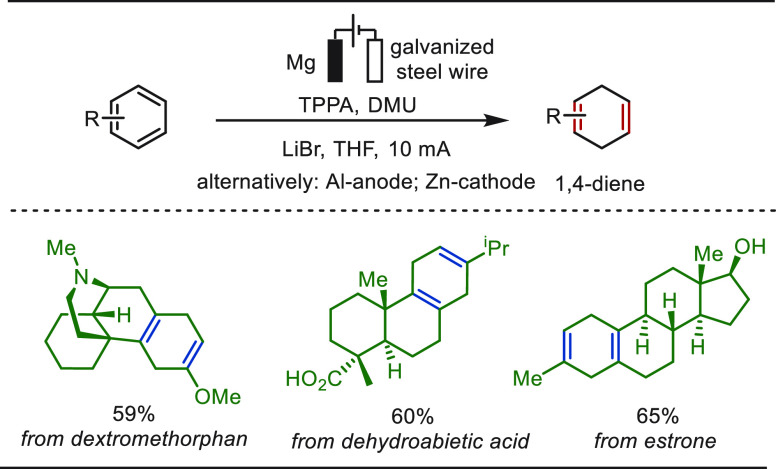
Electrochemical Late-Stage Birch Reduction

The Birch reduction is an important method for the dearomatization of arenes into sp^3^-rich organic molecules.^389,390^ However, the direct use of hazardous alkali metals is necessary for typical Birch reduction. In 2019, Baran reported an exquisite example of electrochemical Birch reduction harvesting Li-ion battery materials and additives (Scheme 83).^391^ The *e*-Birch reduction method operated by consuming a combination of sacrificial anode (Mg or Al), inexpensive proton source dimethylurea, and tris(pyrrolidino)phosphoramide additive for overcharge protection. This method was operationally simple, avoided the direct use of alkali metals maintaining a similar reactivity trend, and exhibited high functional group compatibility along with the application toward the late-stage manipulation of pharmaceutically relevant molecules.

**Scheme 84:**
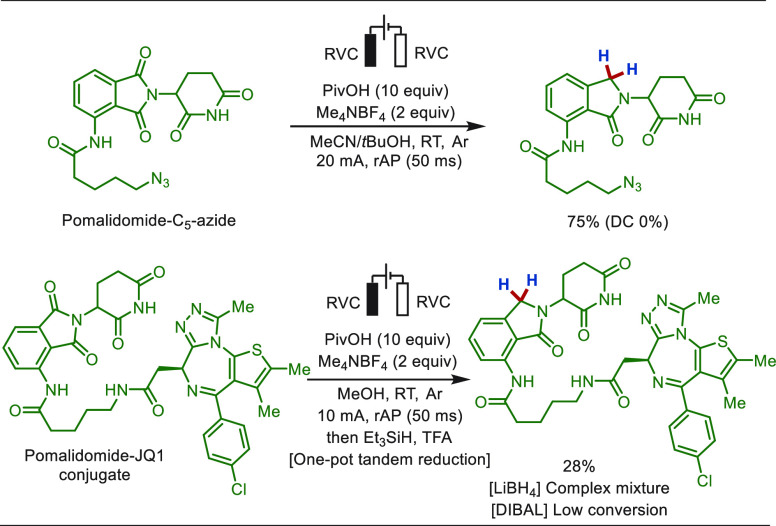
Selective Reduction of Phthalimides under Alternating Current

The vast majority of electrocatalyzed reactions are enabled by direct current, where the polarity of the electrodes remains constant over time and the flow of electrons in the reaction media is unidirectional. While alternating current (AC) was used to realize decarboxylation, nitro reduction, and electrolysis of propylene in the early 20th century,^392−395^ it was rarely explored in mainstream organic synthesis. In 2021, Baran studied the use of AC for a controlled reduction of phthalimides (Scheme 84).^396^ The use of alternating current offered precise control on the transformation, and various sensitive functional groups remained untouched, only selectively reducing the phthalimide motif. Overall, this transformation displayed a broad scope and was employed for the synthesis of PROTAC-relevant molecules.

### 3.6 Other eLSF of Functional Groups

**Scheme 85:**
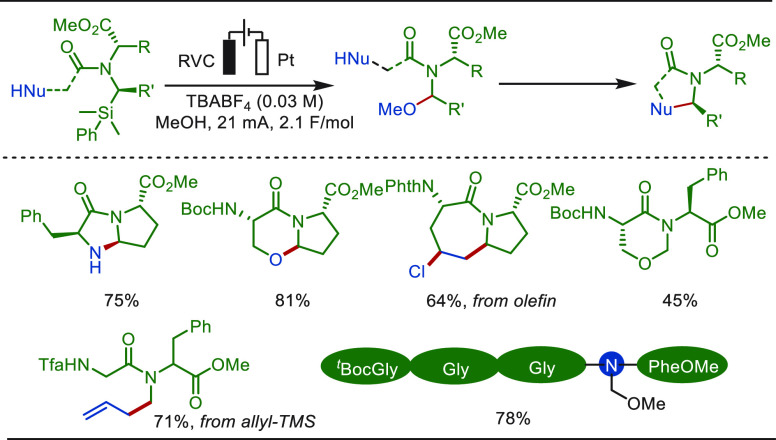
eLSF of Silylated Amino Acids

The direct modification of amino acids and peptides is cumbersome owing to a similar range of oxidation potentials in most of the peptide linkages, leading to site-selectivity issues. The incorporation of a silyl group to the α-position of the amine functionality provides an alternative to execute late-stage modification of peptides. In 2002, Moeller devised an elegant electro-oxidative modification of silylated amino acids, where monocyclic or bicyclic peptidomimetics were easily constructed through an anodic oxidation-based approach (Scheme 85).^397,398^ The electrochemical oxidation of the silylated amino acid led to the formation of acyliminium ion intermediates. The presence of the silyl electroauxiliary reduced the general oxidation potential for the synthesis of acyliminium intermediates from the respective variants without having the electroauxiliary, which amplified the selectivity for the ring construction. These acyliminium intermediates then underwent intramolecular nucleophilic attack with nucleophilic functionalities present in the molecule, forging mono- or bicyclic peptidomimetics.

**Scheme 86:**
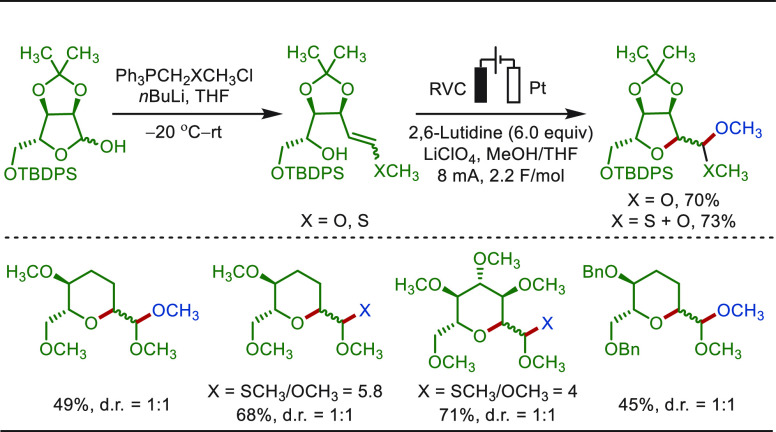
Electrochemical Synthesis of C-Glycosides

Later, Moeller reported on a two-step method involving anodic oxidation as a key step to convert a sugar derivative into C-glycosides consisting of a masked aldehyde functionality (Scheme 86).^399^ The reaction sequence involved a Wittig reaction forming enol ethers, which under electro-oxidative conditions realized an intramolecular nucleophilic attack with the free hydroxy functionality present in the molecule, constructing five- and six-membered C-glycosides in moderate to good yields.

**Scheme 87:**
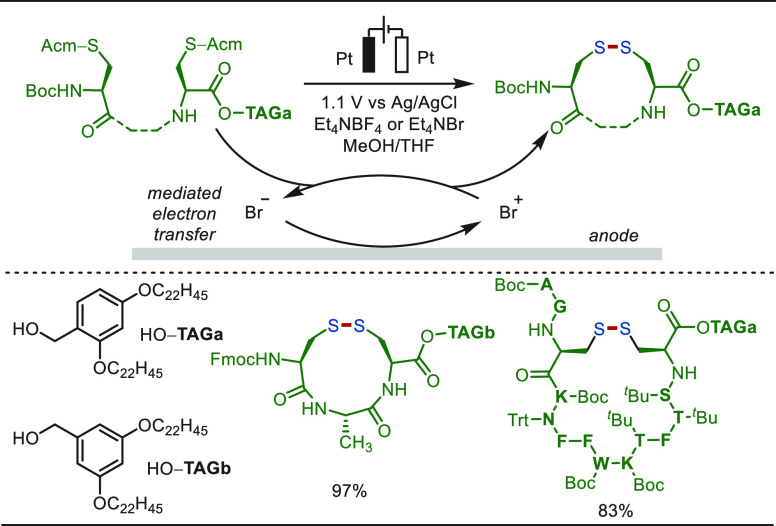
Electrochemical Construction of Disulfide Linkages in Peptides

In 2012, Chiba described an electro-oxidative soluble-support assisted synthesis of disulfide bonds in peptides (Scheme 87).^400^ The method involved bromide ion assisted electron transfer, where the oxidized bromide ion led to disulfide bond formation. Alternatively, the strategy also worked in the absence of bromide ion, where direct oxidation of the substrate was relevant. After the transformation was completed, dilution with acetonitrile, followed by simple filtration, was sufficient to recover the product.

**Scheme 88:**
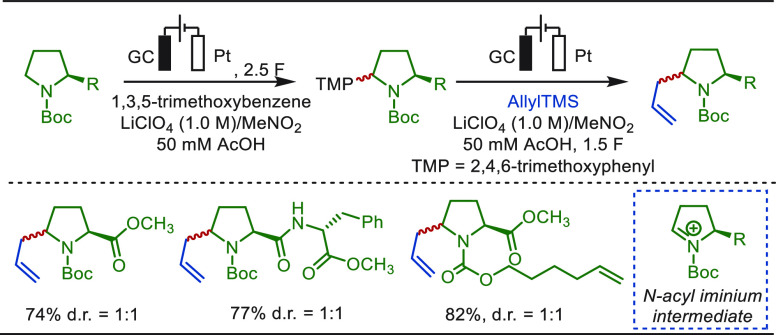
Selective C-5 Functionalization of Proline and Analogues

Another electro-oxidative C–C bond forming approach was unveiled by Chiba group for the modification of *C*- and *N*-terminal proline containing peptides (Scheme 88).^401^ The electrochemical incorporation of the 2,4,6-trimethoxyphenyl (TMP) moiety in the C-5 position of proline led to the selective generation of *N*-acyl iminium intermediates through electro-oxidation of the TMP moiety, which was then trapped with allylTMS to fabricate the allylated products in good yields.

**Scheme 89:**
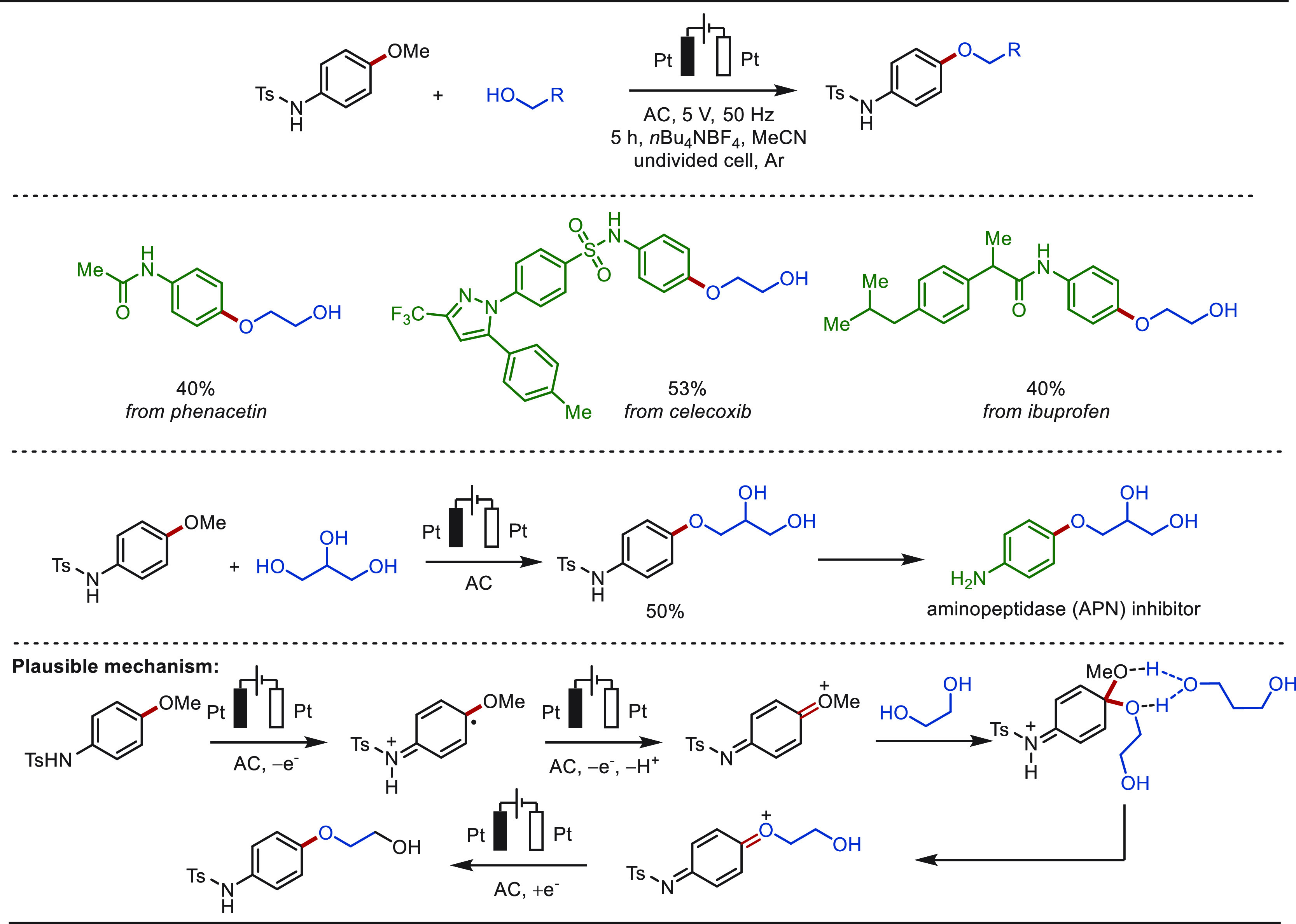
Selective C–O/O–H Metathesis under Alternating Current

Recently, Lei and Huang demonstrated AC promoted C–O/O–H cross-metathesis reactions (Scheme 89).^402^ This AC-based approach allowed for easy transformation of 4-alkoxyanilines into high-value products through electro-oxidation. Single-electron oxidation of these electron-rich arenes generated quinonoid intermediates, which realized a facile nucleophilic attack with the alcohol present in the medium, replacing the methoxy functionality with a new alkoxy functionality. The reaction was operationally simple and chemo- and regioselective. This cross-metathesis reaction was successfully employed for the late-stage diversification of pharmaceuticals and their derivatives.

**Scheme 90:**
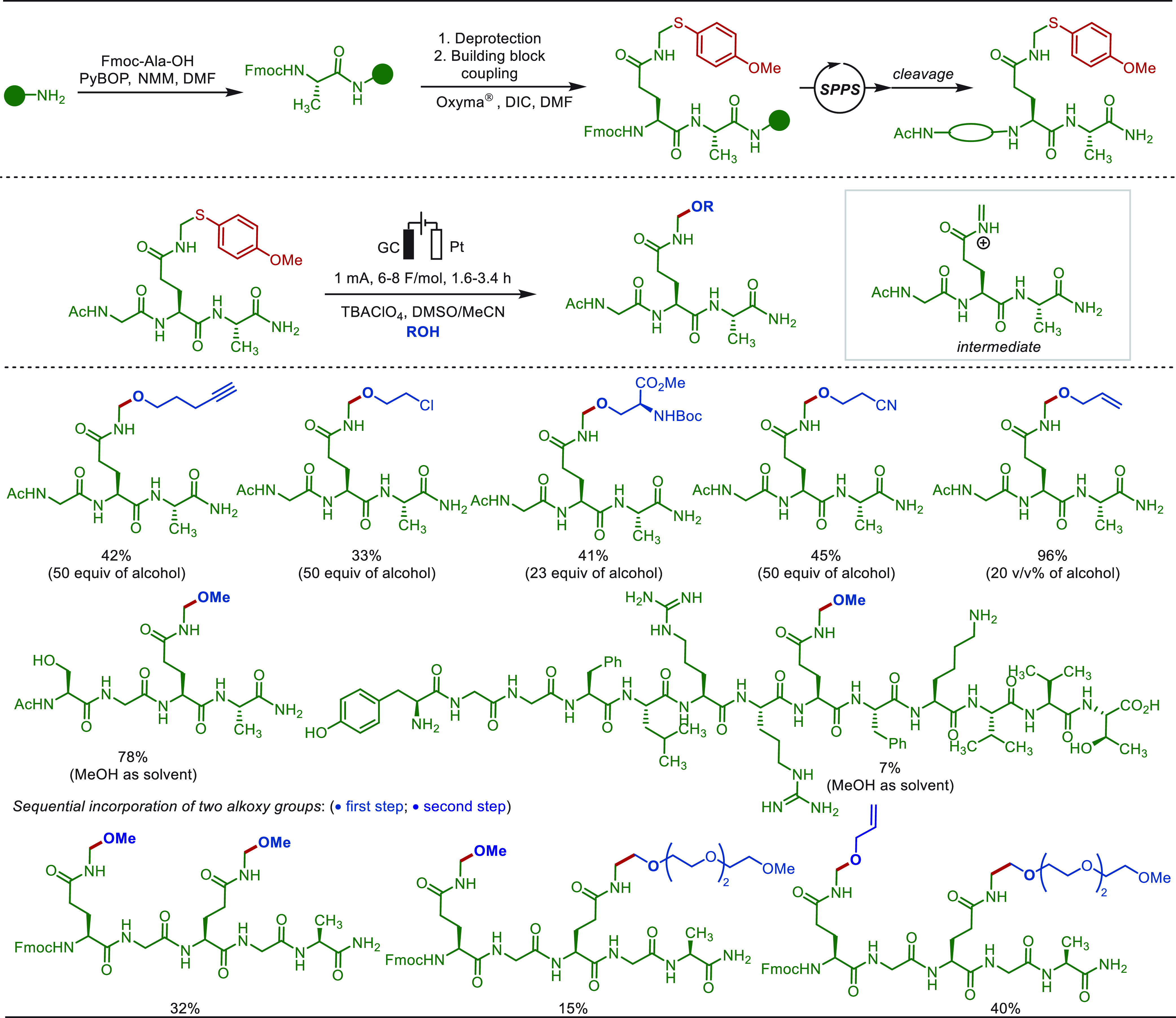
Electroauxiliary-Assisted Late-Stage Functionalization of Peptides

Very recently, Malins and Connal described an electroauxiliary-assisted late-stage diversification strategy of glutamine residues of peptides (Scheme 90).^403^ Peptides consisting of *N*,*S*-acetals in the glutamine residues under electro-oxidative conditions realized a facile cleavage of the thio-functionality to form an iminium intermediate, which in the presence of an alcohol produced respective *N*,*O*-acetals in decent yields. The oxidation potential of electron-rich *N*,*S*-acetals is significantly low, which allowed the strategy to be mild and to tolerate various common oxidation-sensitive functional groups in the peptide chain. This electroauxiliary-based approach served well for the late-stage functionalization of various bioactive peptides.

## 4 Photoelectrochemical LSF of Drug-like Molecules

The photoelectrochemistry is an efficient and sustainable tool for organic synthesis.^98,404−411^ The merger of electrochemistry with photocatalysis^411−417^ combines their advantages and enhances the utility, which has grown rapidly in the past few years and inaugurated a new frontier in synthetic chemistry. Photoelectrochemical catalysis, which requires no exogenous chemical oxidant and generally exhibits a broad functional group compatibility and high selectivity, is ideally suited for the LSF of structurally complex molecules.^418−435^

### 4.1 Photoelectrochemical LSF of C(sp^2^)–H Bonds

**Scheme 91:**
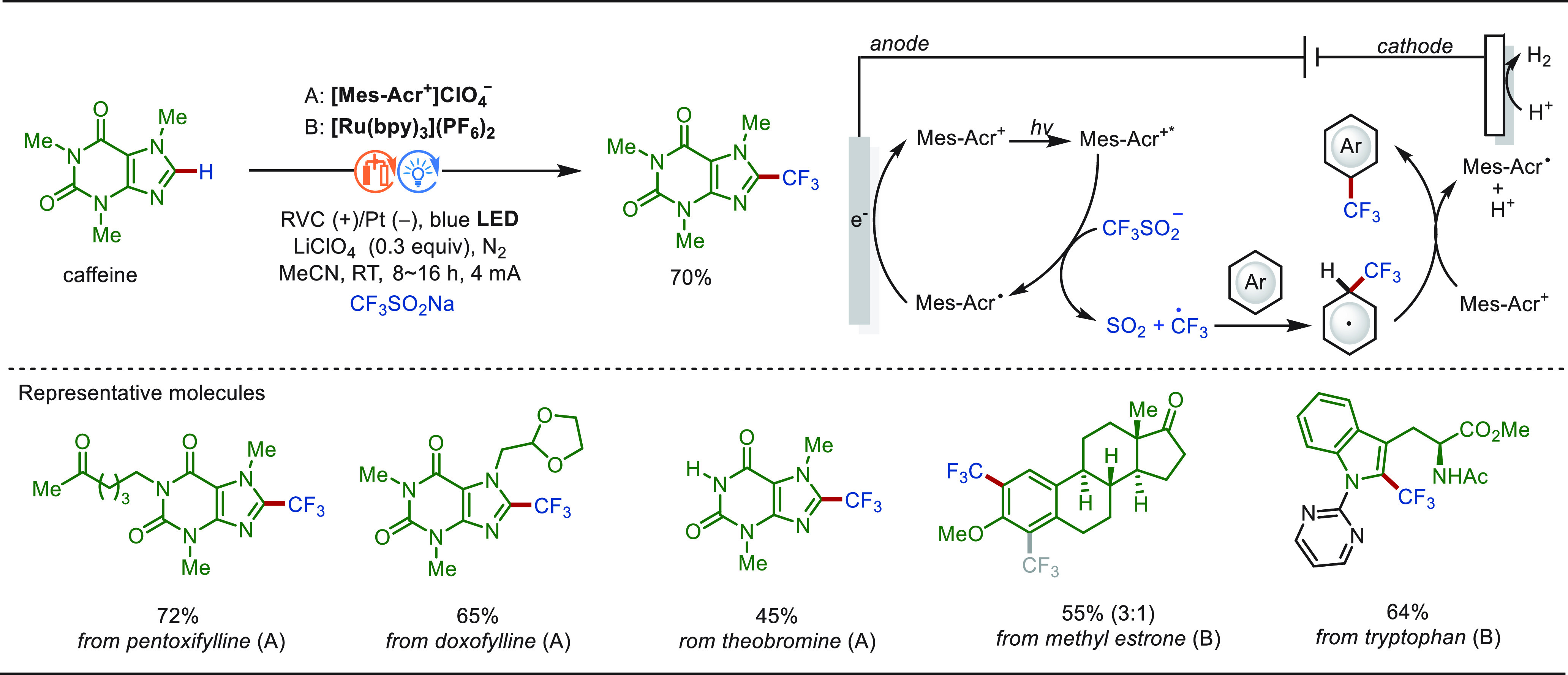
Photoelectrochemical C(sp^2^)–H Trifluoromethylation via Minisci-Type Reactions

In 2020, Ackermann developed a mild photoelectrochemical C(sp^2^)–H trifluoromethylation of arenes with CF_3_SO_2_Na (Scheme 91).^436^ This approach featured a broad substrate scope and high functional groups tolerance. The pe-LSF of natural products, including pentoxifylline, doxofylline, theobromine, methyl estrone, and tryptophan, occurred efficiently. Notably, this photoelectrochemical transformation was also achieved in a flow setup with operationally simple online NMR-monitoring. Mechanistic studies indicated that irradiation of photocatalyst Mes-Acr^+^ led to its highly oxidizing excited state Mes-Acr^+*^. Then, SET between CF_3_SO_2_Na and Mes-Acr^+*^ gave the acridinyl radical Mes-Acr· and a controlled sulfinate radical, which was rapidly converted to a fluoroalkyl radical by cleavage and releasing SO_2_. The stable radical Mes-Acr· was oxidized at the anode to regenerate Mes-Acr^+^, while the alkyl radical was trapped by the heteroarene to give a radical cation, which lost a proton and an electron to give the final product.

**Scheme 92:**
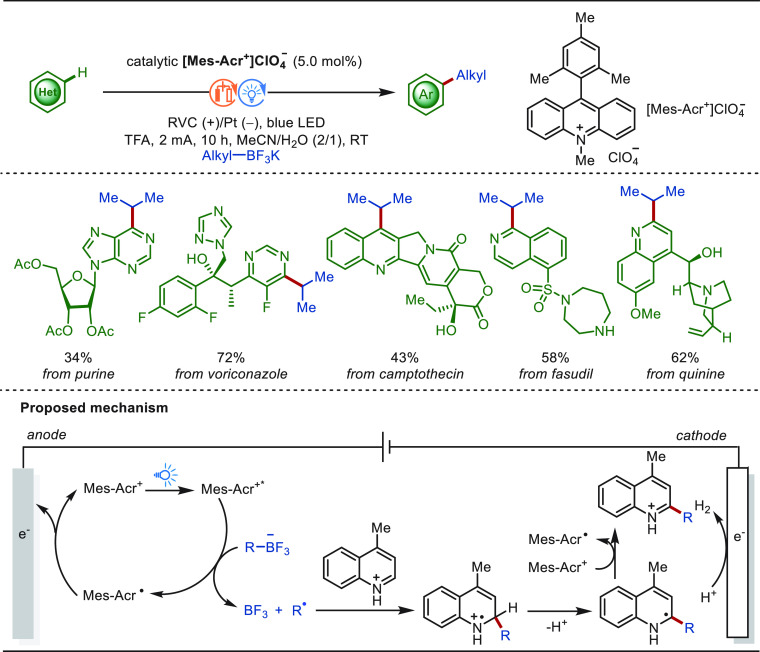
Photoelectrochemical C–H Alkylation via Minisci Reactions

Minisci-type reactions, which furnish net C–H alkylation in heteroarenes by addition of carbon-centered radicals to electron-deficient heterocycles, have attracted broad interest as they provide rapid and direct access to functionalized heterocycles without the need for *de novo* synthesis.^437,438^ In 2019, Xu and co-workers uncovered a photoelectrochemical approach for the late-stage C(sp^2^)–H alkylation of bioactive heteroarenes with stable and easily available organotrifluoroborates (Scheme 92).^439^ This approach featured the generation of alkyl radicals from organotrifluoroborates without an exogenous chemical oxidant, and various heteroarenes were functionalized with excellent site selectivity and chemoselectivity. Notably, the challenging α-alkoxyl and tertiary radicals also reacted successfully under mild conditions.^440^ The catalytic cycle is initiated by the irradiation and oxidation of Mes-Acr^+^, and thus they are prone to undergo single-electron transfer with organotrifluoroborates.

**Scheme 93:**
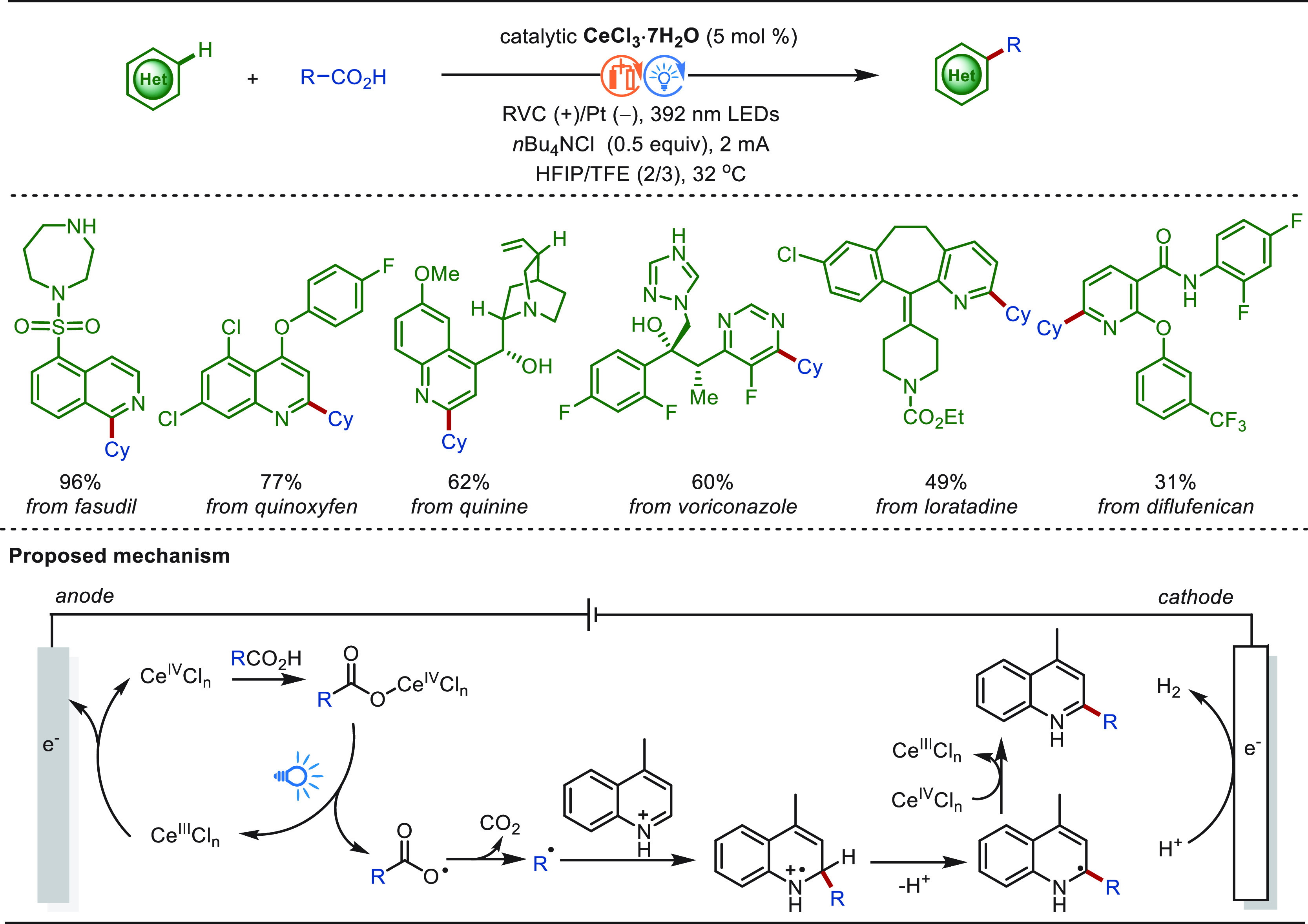
Photoelectrochemical Decarboxylative C–H Alkylation

In 2020, Xu and co-workers described a photoelectrocatalytic decarboxylative C(sp^2^)–H alkylation of heteroarenes under the catalysis of CeCl_3_·H_2_O and 4CzlPN in a mixture of HFIP/TFE (Scheme 93).^441^ Carboxylic acids and oxamic acids were used as the alkyl and carbamoyl sources, respectively. These reactions proceed through photoinduced ligand-to-metal charge transfer (LMCT),^442−444^ which upon decarboxylation generate the alkyl or carbamoyl radicals. The radicals are then trapped with the protonated lepidine and undergo the HER to produce the desired product. Advantageously, this efficient method was scalable to decagram amounts and applicable for the direct C–H alkylation and carbamoylation of drug molecules, such as fasudil, quinoxyfen, quinine, and voriconazole.

**Scheme 94:**
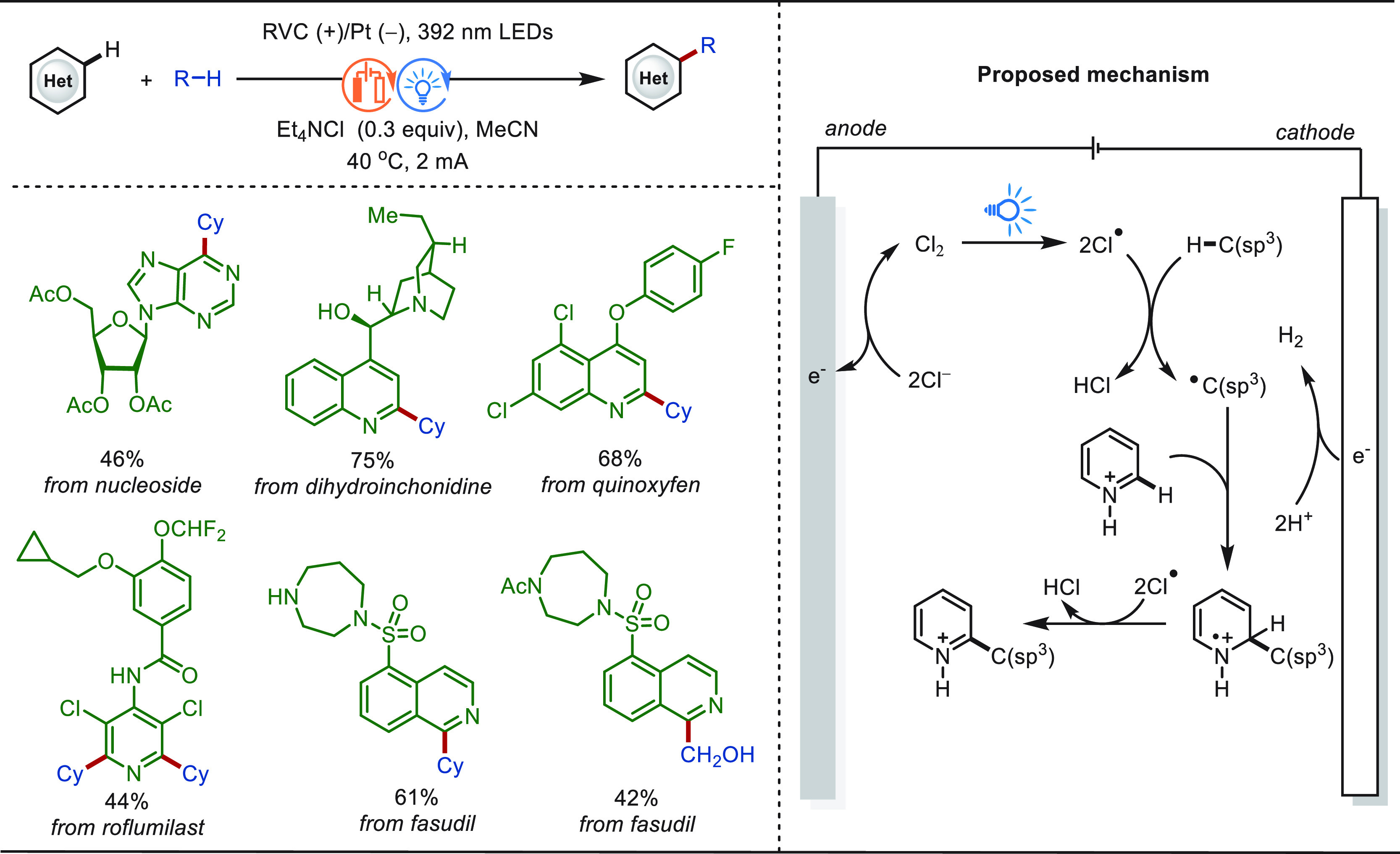
Photoelectrochemical C–H Alkylation with Alkanes

Subsequently, the same group reported an elegant dehydrogenative alkylation of heteroarenes with aliphatic C(sp^3^)–H bonds under photoelecrtochemical conditions (Scheme 94).^445^ This strategy obviated the use of transition-metal catalysts or chemical oxidants and achieved efficient coupling of a variety of C(sp^3^)–H donors with a wide range of heteroaromatic drug molecules including quinoxyfen, roflumilast, and fasudil. Mechanistic studies indicated that the C(sp^3^)–H donor was transformed to a nucleophilic carbon radical through hydrogen-atom transfer (HAT) with chlorine radical, which was generated by light irradiation of anodically produced Cl_2_.^446^ The key carbon radical then underwent radical substitution to the protonated heteroarene to afford the alkylated products.

**Scheme 95:**
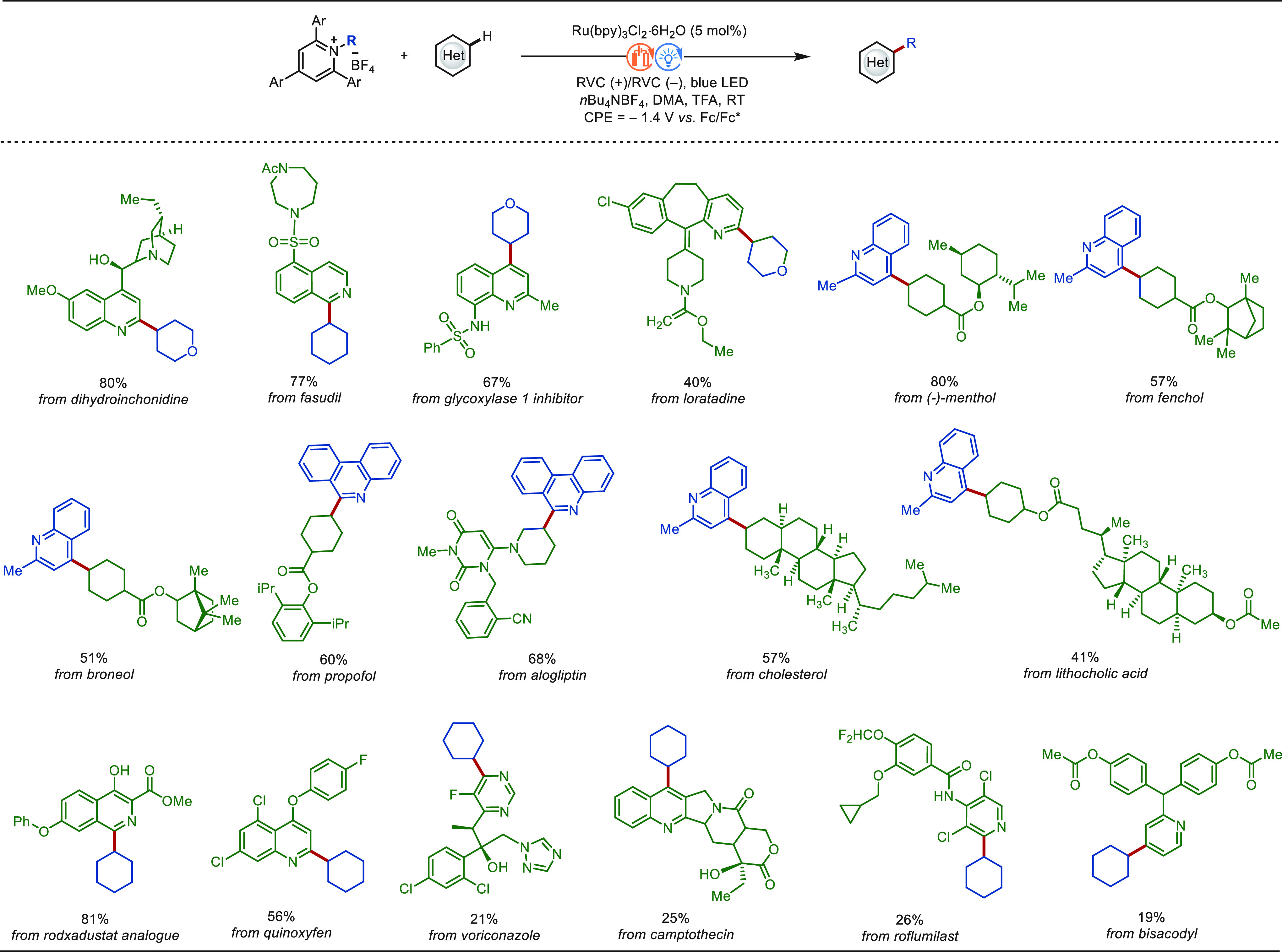
Photoelectrochemical C–H Alkylation with Bench-Stable Katritzky Salts

Bench-stable Katritzky salts have been used as an alkylation reagent in various transformations. In this context, Chen and co-workers recently developed a photoelectrocatalytic deaminative alkylation approach (Scheme 95).^447^ Mechanistic studies indicated that the homogeneously dispersed photocatalyst was essential for the efficient generation of alkyl radicals. Notably, the practicability and robustness of this method were highlighted by the late-stage functionalization of a great number of bioactive molecules.

**Scheme 96:**
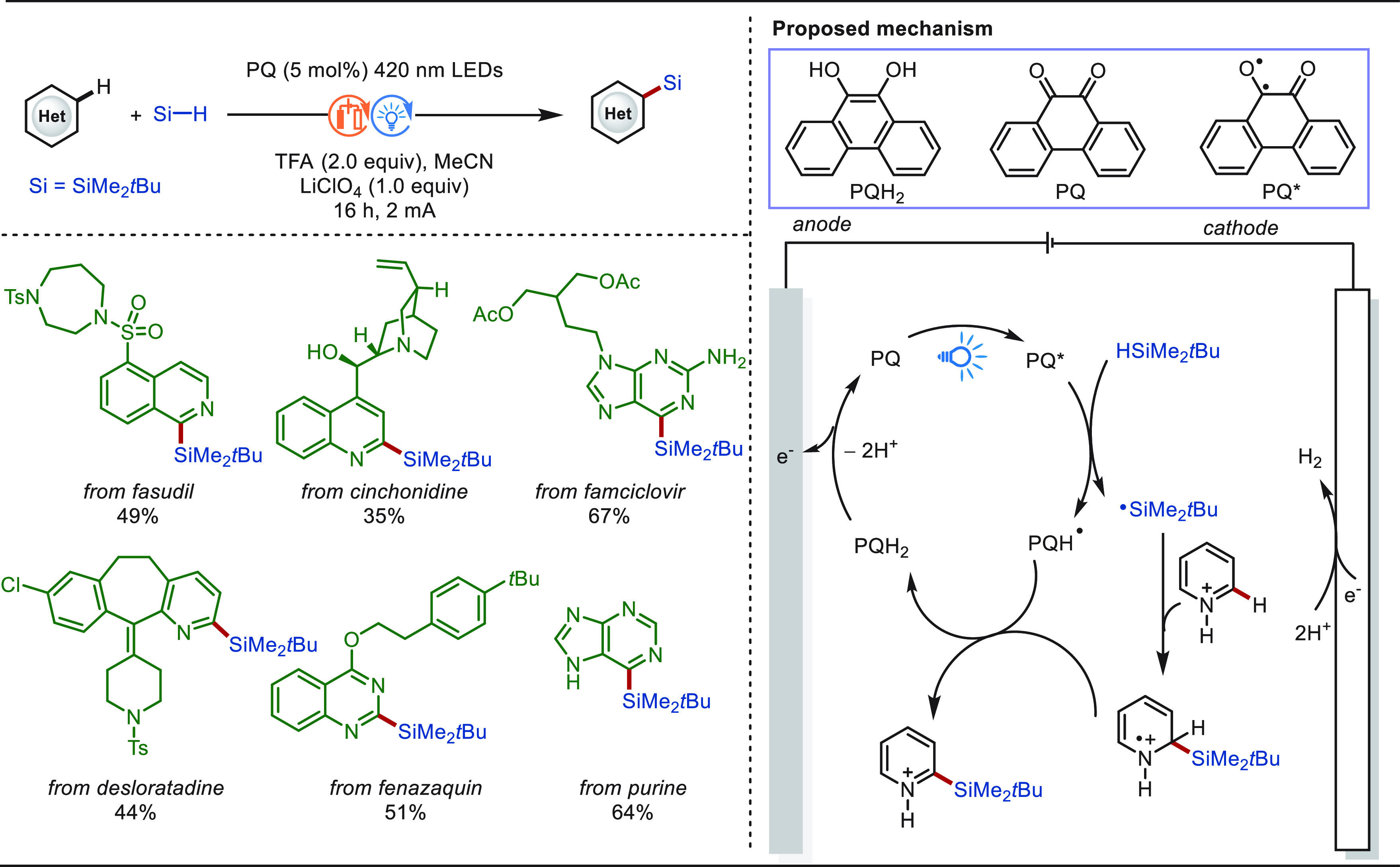
Photoelectrochemical C–H Silylation of Heteroarenes

Recently, Wang and Hou developed a photoelectrocatalytic C–H silylation of heteroarenes by dehydrogenative cross-coupling with H_2_ evolution, which employed 9,10-phenanthrenequinone (PQ) as a photoelectrocatalyst (Scheme 96).^448^ This strategy avoided the use of an external oxidant or HAT reagent, and a variety of heteroaromatic molecules can be compatible with excellent site-selectivity and desirable yields. Mechanistic studies indicated that the dual function of 9,10-phenanthrenequinone (PQ) enabled the process of the photoelectrocatalytic cycle and hydrogen atom transfer from *t*BuMe_2_SiH to afford *t*BuMe_2_Si· and PQH·, which later reacted with heteroarenes to afford the ultimate product. The photoelectrocatalytic late-stage C–H silylated tactics could be well wielded in the formation of heteroaromatic bioactive molecules, including fasudil, cinchonidine, famciclovir, desloratadine, fenazaquin, and purine.

**Scheme 97:**
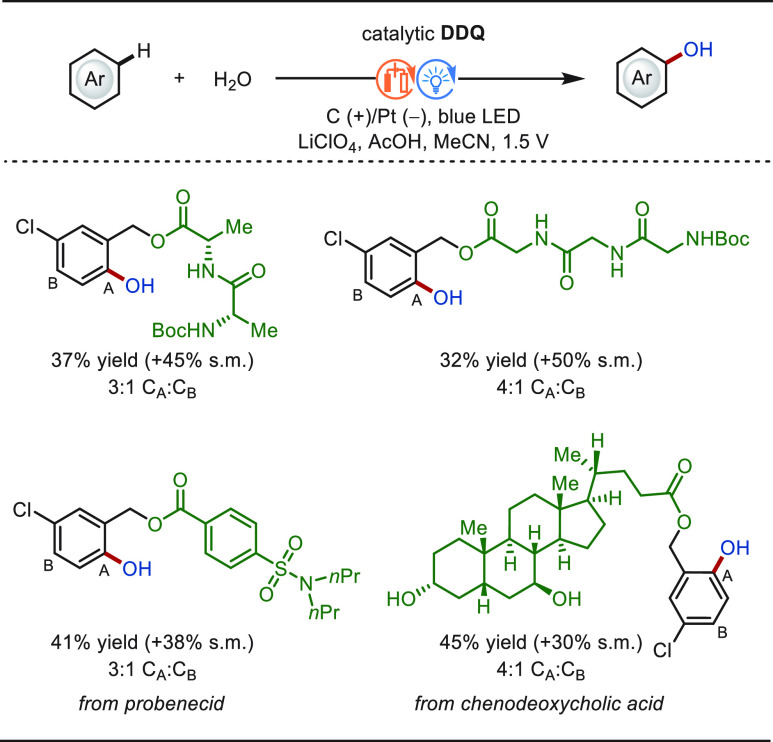
Photoelectrochemical Hydroxylation of Aryl C–H bonds

In 2021, Lambert reported a photoelectrocatalytic hydroxylation of arenes by the catalysis of 2,3-dichloro-5,6-dicyanoquinone (DDQ) with visible-light irradiation (Scheme 97).^449^ Mechanistic studies indicated that the process would be implemented by recycling of DDQ, wherein photoexcited DDQ oxidized arenes through single-electron transfer (SET) to furnish a radical cation that underwent nucleophilic capture. DDQ would be regenerated by anodic oxidation, with H_2_ released at the cathode.

### 4.2 Photoelectrochemical LSF of C(sp^3^)–H Bonds

**Scheme 98:**
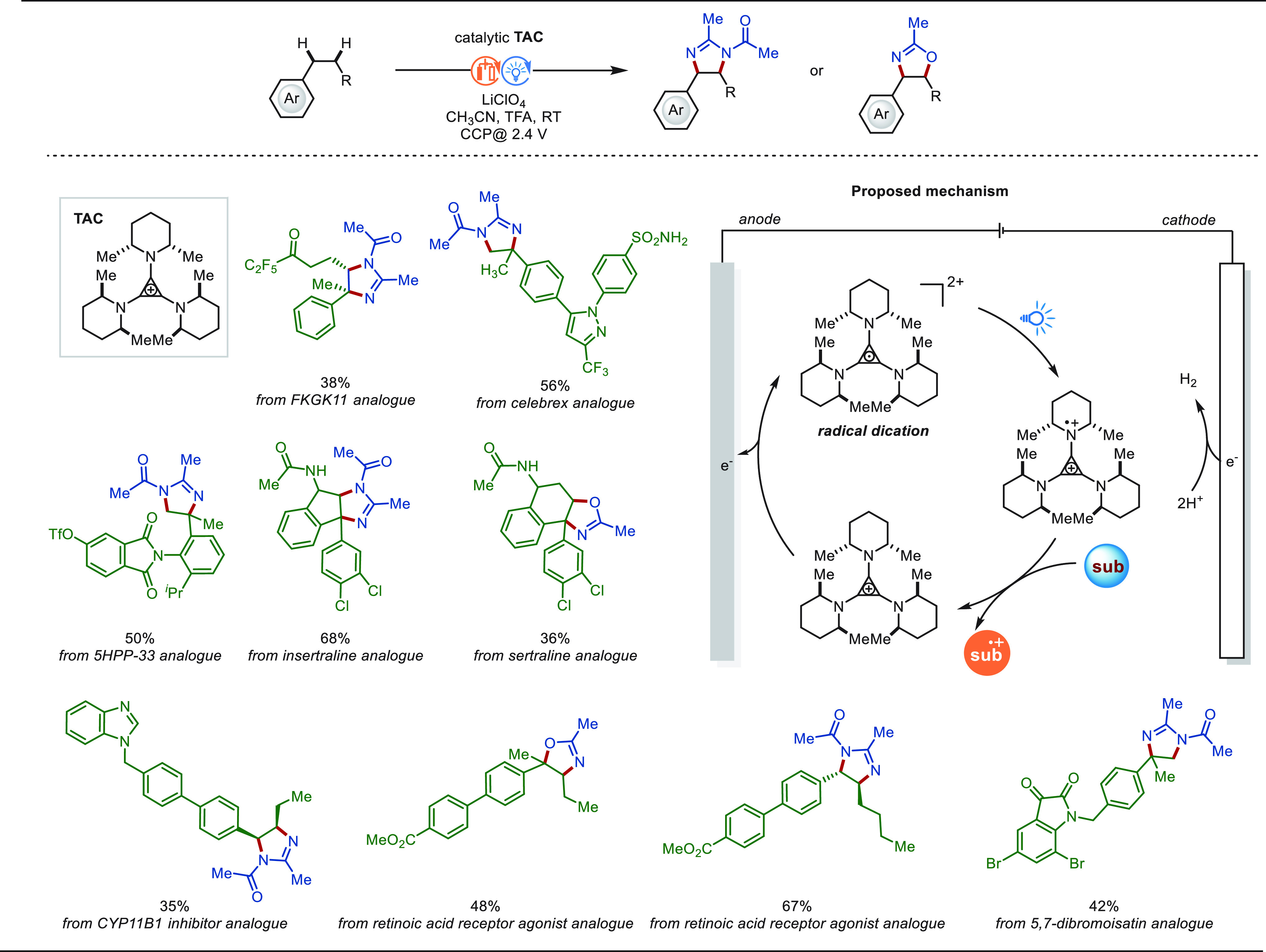
Photoelectrochemical Diamination of Vicinal C–H Bond

In 2021, Lambert demonstrated a versatile photoelectrocatalytic diamination of vicinal C–H bonds under the catalysis of trisaminocyclopropenium (TAC) ion in the absence of external oxidants (Scheme 98).^450^ Photoelectrochemical conditions activate the TAC catalyst to a stable radical dication by anodic oxidation, while the cathodic reaction reduces protons to H_2_. Irradiation of the TAC radical dication with a light generates a strongly oxidizing photoexcited intermediate, which was an extremely potent oxidant and underwent an electron transfer with olefin followed by a Ritter-type functionalization of C–H bonds with the acetonitrile, a common solvent, as the nitrogen source. Notably, depending on the nature of the electrolyte, both 3,4-dihydroimidazole and 2-oxazoline products were obtained by using simply visible light and a mild electrochemical reaction condition. This photoelectrocatalyzed approach enabled the difunctionalization of a number of antitumor and antiviral active medicinal compound.

**Scheme 99:**
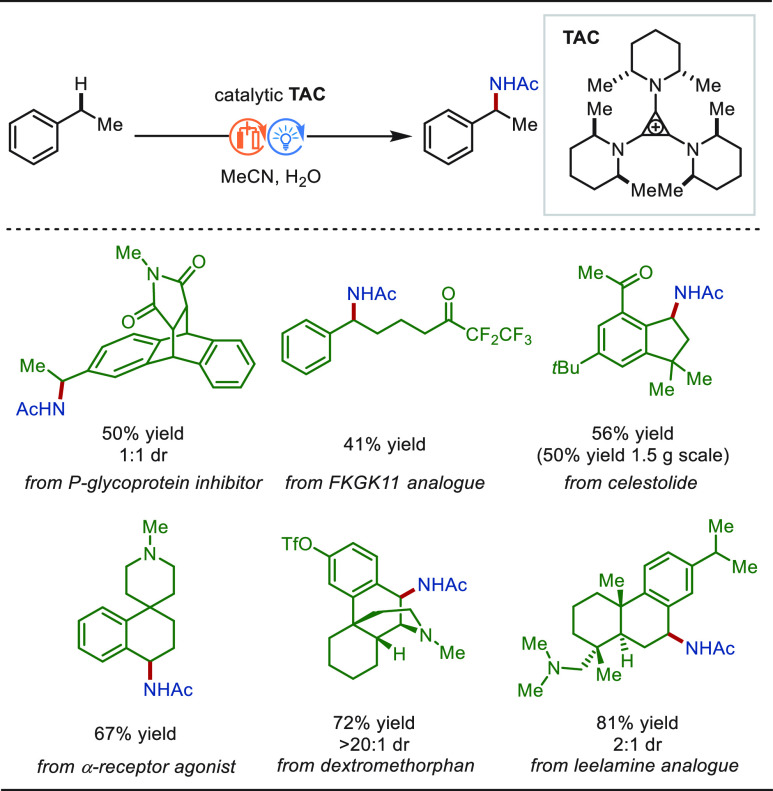
Photoelectrochemical Amination of Benzylic C(sp^3^)–H Bond

On a different note, Lambert reported an elegant C–H bond amination by the catalysis of trisaminocyclopropenium (TAC) ion in an electrochemical divided cell under white-light compact fluorescent light, where the reaction proceeded through anodic oxidation generating the stable radical dication (Scheme 99).^451^ Irradiation of radical dication leads to the photoexcited intermediate TAC*, which engages in single-electron oxidation of the arene substrate to generate the key radical cation. Subsequently, the reaction proceeds through the classic Ritter steps with an acetonitrile solvent to form the aminated product. It should be stressed that the introduction of an acetamide moiety could mitigate the kinetic rate of single-electron oxidation by TAC, which would avoid the risk of overoxidation of the C–H amination chemistry. Photoelectrocatalytic processes are more efficient and suitable for the late-stage functionalization of complex natural products than the direct electrochemical one.

**Scheme 100:**
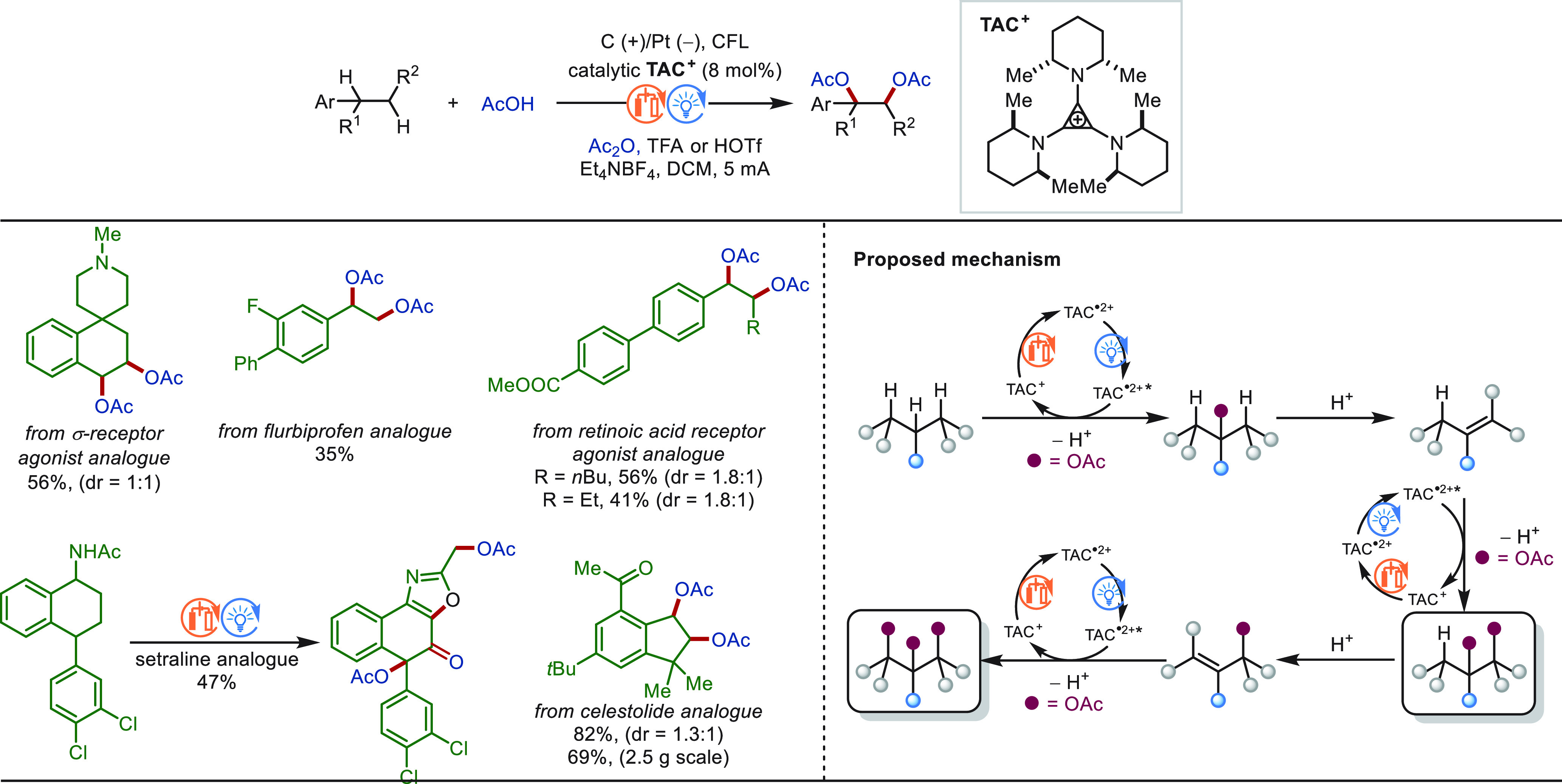
Photoelectrocatalytic Multiple C–H Bonds Oxygenations

Subsequently, Lambert and Ye reported photoelectrocatalytic selective oxygenation of either two or three contiguous C(sp^3^)–H bonds, which exploited a trisaminocyclopropenium (TAC) ion intermediate as a potent oxidative catalyst with excellent selectivity to restrain overoxidative decomposition (Scheme 100).^452^ Mechanistic investigations reveal that the process involves the sequential oxidation of C(sp^3^)–H bonds through a relay mechanism. In this intricate process, electro-oxidative and photoexcited triplet aryl cation (TAC) species facilitate the oxidation of C(sp^3^)–H bonds through a single-electron transfer (SET) process to form radical cations capable of nucleophilic trapping, followed by the giving rise to monooxygen products. In the presence of acidic conditions, the monooxygenated intermediate could undergo a gradual and reversible elimination process, leading to the formation of an olefin. As a result, it can further react to form the dioxygenated adduct or even undergo multiple oxidation events, leading to the formation of di- or trioxygenated products. Notably, the choice of acid (TFA or HOTf) allows for the selective synthesis of two or three contiguous C–O bonds. E1-type elimination was believed to be a critical step and is able to achieve a third C–H oxygenation by using the stronger HOTf acid. The utility of this method was demonstrated by the late-stage modification of bioactive molecules.

**Scheme 101:**
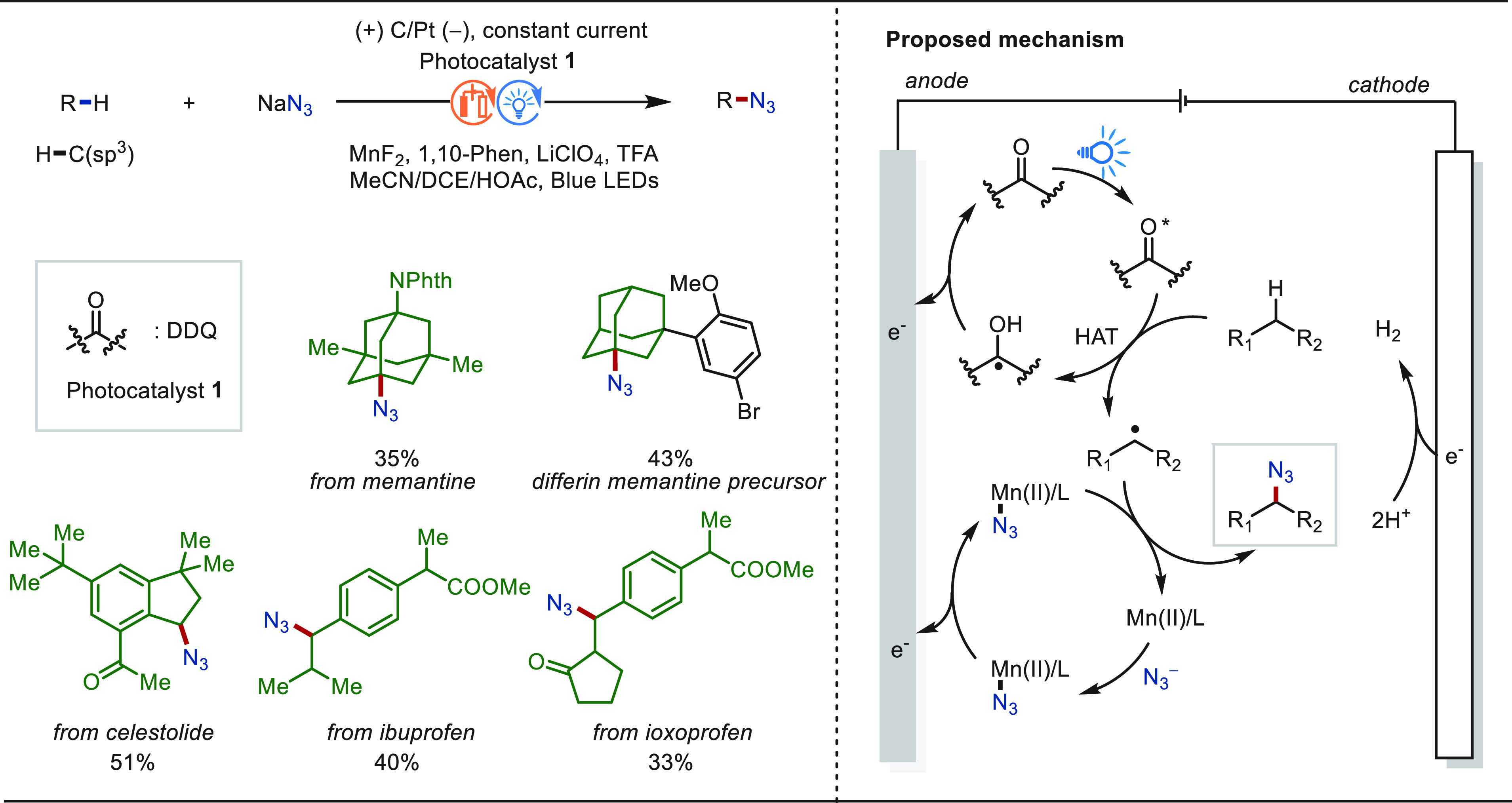
Photoelectrochemical Manganese-Catalyzed C(sp^3^)–H Azidation

The azide group is a versatile moiety that can be easily reduced to free primary amines, which are featured in pharmaceutical discovery and late-stage functionalization. In 2020, Lei and co-workers have disclosed a manganese-catalyzed azidation of C(sp^3^)–H bond by merging of visible-light catalysis and electrochemical oxidation (Scheme 101).^453^ The ketone photocatalysts (DDQ, 9-fluorenone, or 4,4′-dimethoxybenzophenone) were used to generate a C(sp^3^)-centered radical intermediate via a HAT pathway. In this process, coordination of NaN_3_ to the manganese(II) followed by anodic oxidation of manganese(II)/L–N_3_ intermediate generated the manganese(III)/L–N_3_ intermediate, which furnished a suitable azide radical to generate the azide product. Meanwhile, molecular hydrogen was evaluated at the cathode. Under the photoelectrocatalytic conditions the late-stage azidation of various valuable drug-like molecules could be achieved.

**Scheme 102:**
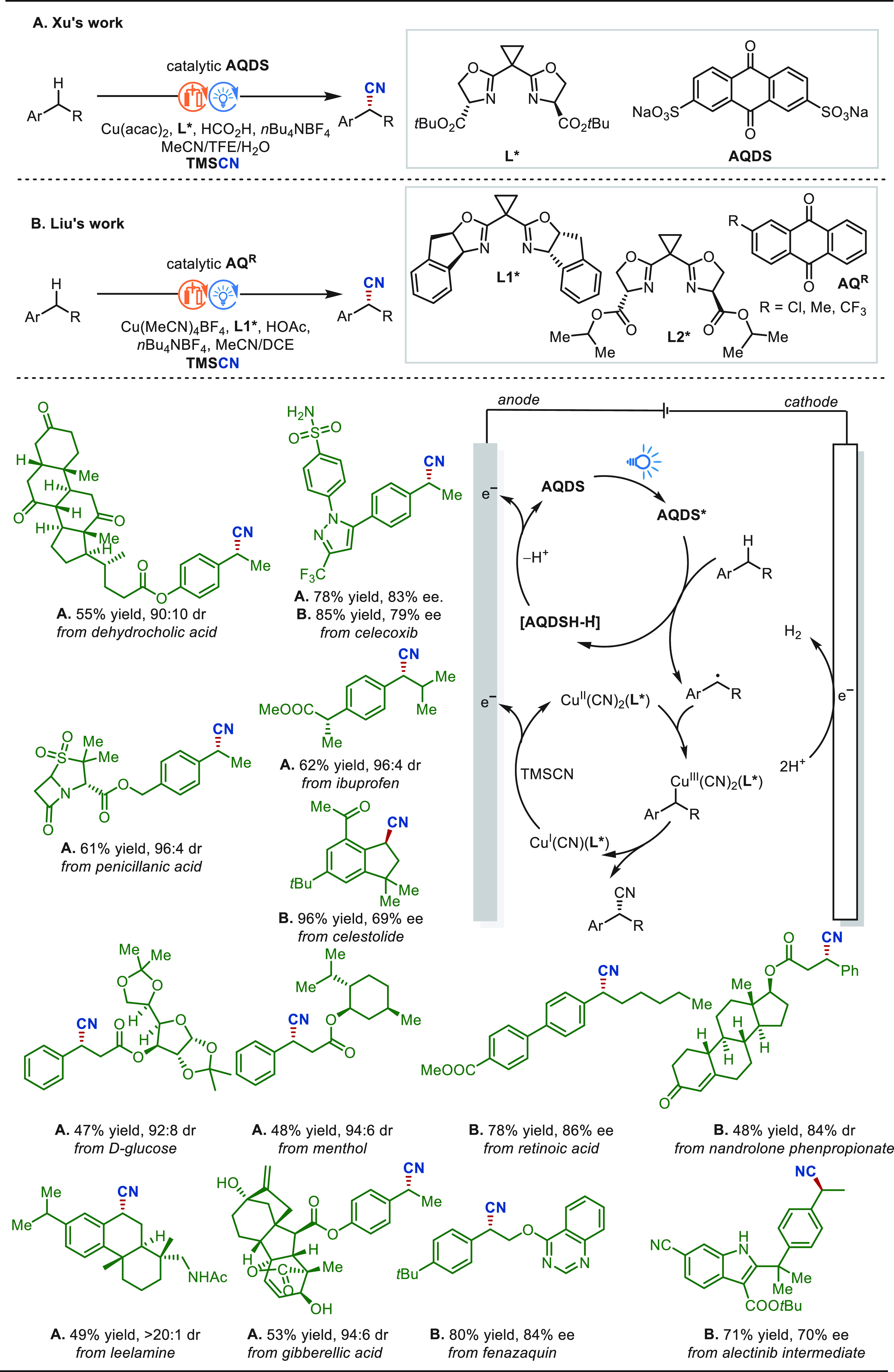
Photoelectrochemical Asymmetric Cyanation of Benzylic C–H Bonds

Asymmetric C(sp^3^)–H functionalization has proven to substantially shorten the process of late-stage modification of complex molecules.^4,454,455^ Recently, Xu and Liu independently disclosed the first photoelectrocatalytic asymmetric functionalization of C–H bonds (Scheme 102).^456,457^ The asymmetric photoelectrocatalytic cyanation reaction proceeded in the presence of a copper catalyst in combination with a photocatalyst, featuring a broad substrate scope without the use of any chemical oxidant. The late-stage cyanation of a range of complex structures was achieved in excellent yields with high site selectivity and enantioselectivity. Mechanistic studies suggested the following mode of action: photocatalyst AQDS upon irradiation formed the electronically excited photocatalyst ADQS*, which underwent a single-electron oxidation generating a benzylic radical. The benzylic radical then combined with the chiral copper complex (L*)copper(II)(CN)_2_ and then after reductive elimination released the desired cyanation product.^458,459^ The reduced (L*)copper(I)(CN) and intermediate (AQDS–H)· underwent anodic oxidation to regenerate (L1*)copper(II)(CN)_2_ and AQDS, along with H_2_ evolution at the cathode. This merger of photoelectrocatalysis and asymmetric copper-catalyzed radical cyanation^460^ enabled modular control to the radical relay catalysis of various C–H functionalizations.^461,462^

### 4.3 Miscellaneous Photoelectrochemical LSF

**Scheme 103:**
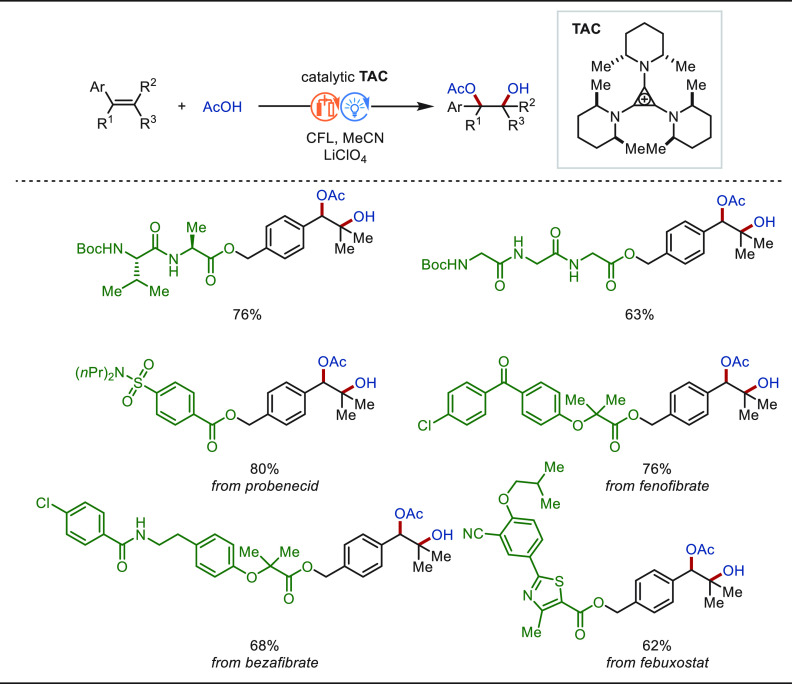
Photoelectrochemical Acetoxyhydroxylation of Aryl Olefins

In 2019, Lambert exhibited an elegant approach to acetoxyhydroxylation of aryl olefins with the photoelectrocatalytic trisaminocyclopropenium (TAC) ion intermediate with a controlled electrochemical potential under visible light irradiation (Scheme 103).^463^ Specifically, this method is triggered by single-electron transfer of the olefin substrate by strongly oxidizing intermediate TAC·^2+^* to generate an olefin radical cation, which is subsequently trapped with AcOH and further oxidized to an oxocarbenium intermediate, followed by hydrolysis to release the acetoxyhydroxylative product. This approach was applied to the derivatization of complex structures and a range of functional modification.

**Scheme 104:**
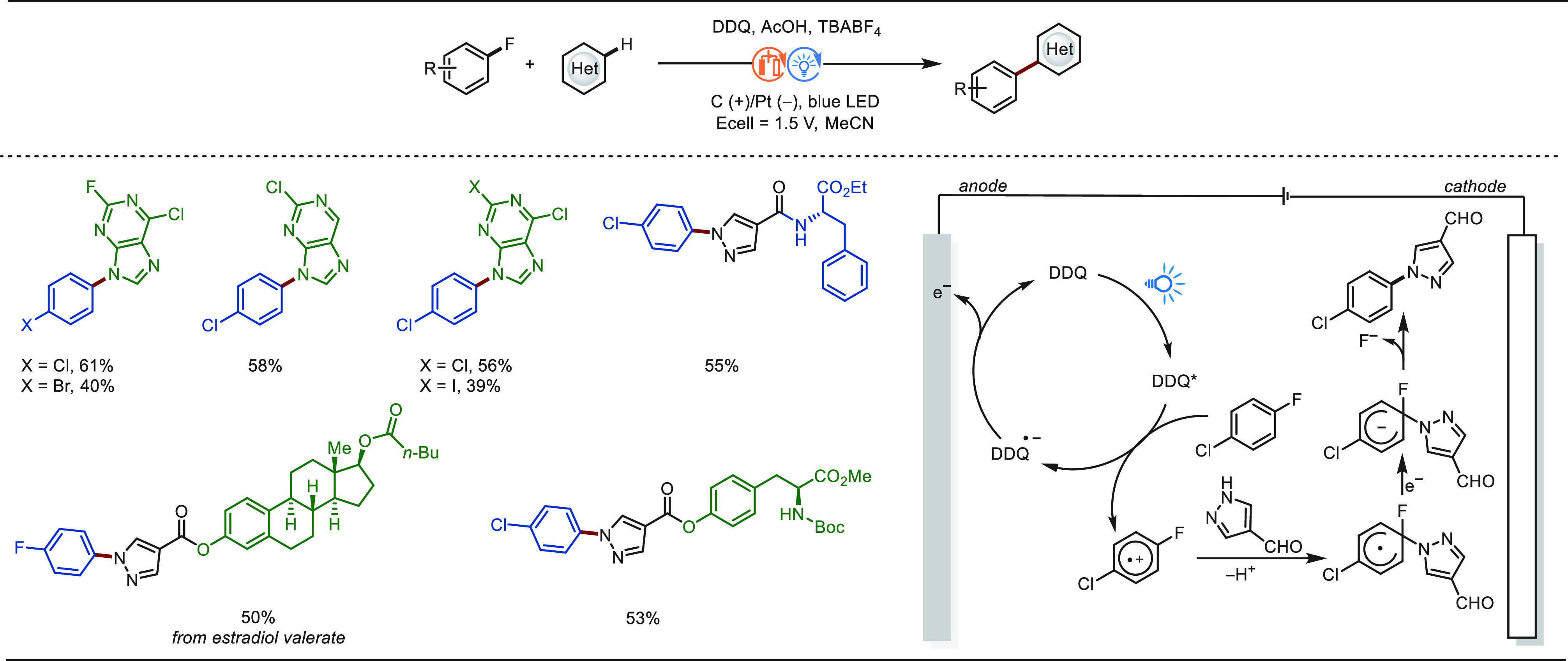
Photoelectrocatalytic Nucleophilic Aromatic Substitution Reaction

In 2020, Lambert disclosed an photoelectrocatalytic nucleophilic aromatic substitution reactions of unactivated aryl fluorides under exceedingly mild conditions (Scheme 104).^464^ Mechanistic investigations revealed that the photoexcitation of DDQ produced an excited state species, which is sufficient to facilitate the single-electron oxidation of fluoroarene. The resulting radical cation processed nucleophilic attack by pyrazole and then generated an aryl radical, which underwent cathodic reduction and the fluoride group left to furnish the corresponding product. The applicability of this approach was demonstrated by the late-stage diversification and syntheses of the drug molecules.

**Scheme 105:**
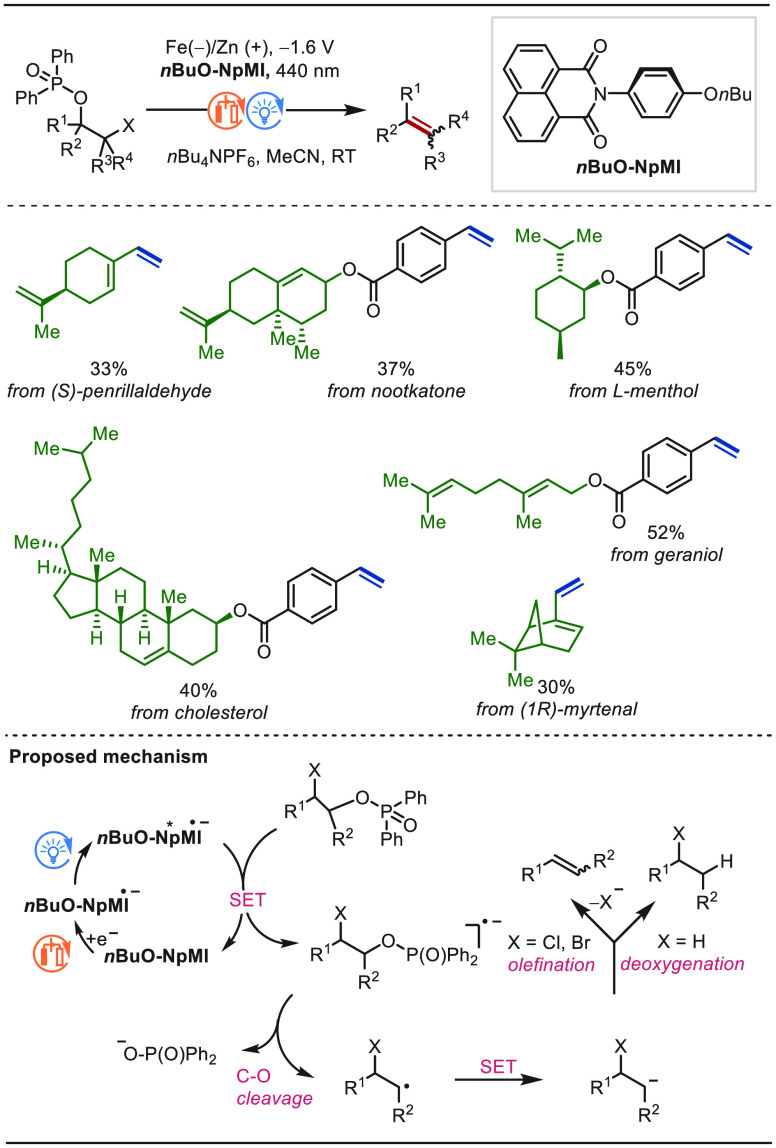
Photoelectrochemical C(sp^3^)–O Cleavages of Phosphinated Alcohols to Carbanions

In 2021, Barham and co-workers reported an efficient photoelectrocatalytic method in the reductions of phosphinates derived from α-chloroketones toward olefination and deoxygenation by using 2,6-diisopro-pylphenyl-containing naphthalenemonoimide (NpMI) as a catalyst (Scheme 105).^465^ A plausible mechanism of the photoelectrocatalysis cycle involves cathodic reduction and photoexcitation to afford a strongly potent reductant. Subsequently, the SET reduction of phosphinates species to its radical anion followed by C(sp^3^)–O bond cleavage generates a benzyl radical.^466^ The benzyl radical undergoing reduction to the corresponding carbanion intermediate would further enable either an olefination or a deoxygenation. Surprisingly the photoelectrocatalytic method tolerated aryl chlorides/bromides with similar or more accessible reductive potentials and operated under mild conditions, which favored the late-stage functionalization of complex drug molecules.

**Scheme 106:**
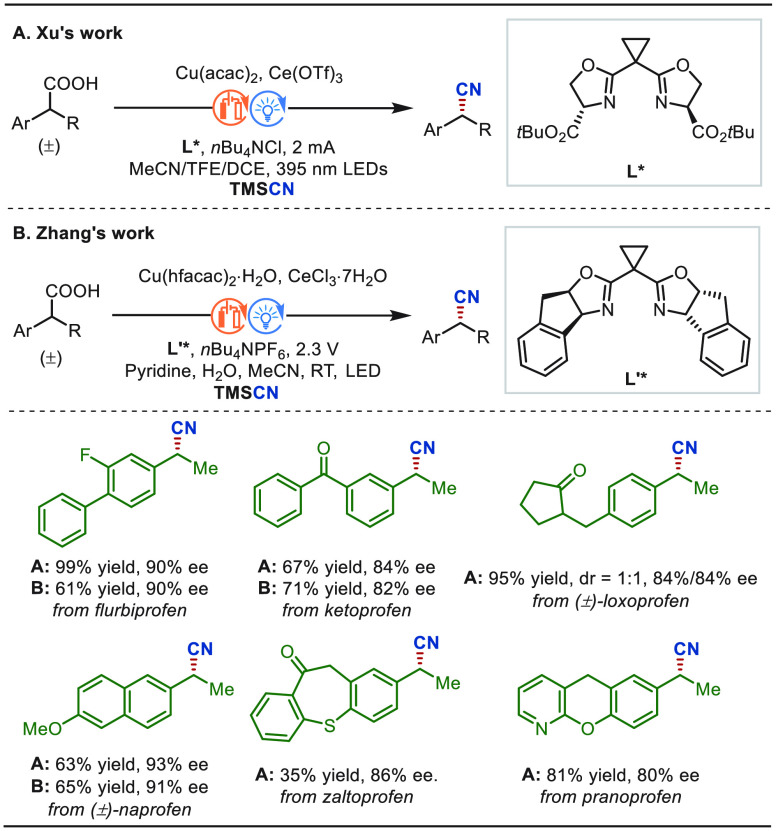
Photoelectrochemical Asymmetric Decarboxylative Cyanation

Based on the success of the proof-of-concept asymmetric photoelectrocatalytic approach, Xu subsequently devised a photoelectrochemical enantioselective decarboxylative cyanation, which converted racemic carboxylic acids directly to enantioenriched nitriles (Scheme 106A).^467^ This method employed a cerium(III) salt and a chiral copper(II)(L*) as the relay catalysis, which proceeded through the cerium salt for photoelectrocatalytic decarboxylation and a chiral copper complex for enantioselectivity control. Key to the success was the ideal incorporation of photoelectrocatalytic tactics with asymmetric copper catalytic cycle, and both catalysts were regenerated by anodic oxidation. The photoelectrochemical asymmetric approach converted straightforwardly commercial carboxylic-acid-based drug molecules to their corresponding enantioenriched nitriles, including flurbiprofen, ketoprofen, loxoprofen, naproxen, zaltoprofen, and pranoprofen. Coincidentally, the same photoelectrochemical strategy, in which cerium(III) salt and copper(II)(BOX*) were employed as cocatalysts to promote the catalytic decarboxylation and asymmetric cyanation was reported by Zhang (Scheme 106B).^468^

## 5 Conclusion and Perspective

Over the past decade, electrocatalysis has undergone a remarkable renaissance and has been identified as an increasingly robust tool in molecular sciences. Consequentially, significant recent momentum has been gained by applications of molecular electrochemistry to late-stage functionalization (LSF). Thus, eLSF was accomplished with a variety of natural products, drugs, peptides, proteins, as well as other bioactive molecules bearing complex structures, typically featuring exceedingly mild conditions with a high level of selectivity control. These eLSFs are characterized by remarkable advantages, namely, the use of electrons and protons as sustainable redox agents, obviating the need of chemical oxidants/reductants; new mechanistic routes enabling reactions to proceed under ambient temperature rather than superheated conditions; direct formation of target molecules via electrolysis, greatly shortening the synthetic steps; exhibiting increased selectivity or different selectivity compared to traditional methods; accomplishing specific transformations that could not be achieved before; and so on. Notably, the application of electrochemical continuous-flow techniques, which feature improved mass transfer, low residence times, and high surface-to-volume ratios, has greatly accelerated the development of the LSF field by increasing reaction efficiency, reducing overoxidation, and simplifying scale-up. In addition, the merger of electrochemistry with photochemistry, so-called photoelectrochemistry, shows huge application potential and has yet to make several dazzling achievements.

Despite the remarkable recent progress, the electro-organic chemistry as well as the eLSF arena are arguably still in their infancies. Here we address the present limitations of eLSF, which might also represent some valuable research directions in the future.

i) The established eLSF methodologies are still rather limited considering the huge demand in drug-discovery programs. For instance, the reported late-stage methylation only occurs in the presence of several restricted nitrogen-oriented directing groups. The development of general strategies for the electrochemical late-stage methylation of complex molecules bears a significant synthetic space. Similarly, strategies describing straightforward and selective inclusion of functionalities such as trifluoromethyl, halogens, methoxy, amino, hydroxy, nitro, methylamino, ethoxy, carbonyl, etc., which comprise high relevance in medicinal chemistry and determine the structure–activity relationship, are limited. Thus, there is a need for further exploration toward increasing the scope of substrate classes and their widespread application in eLSF reactions. The incorporation of a fluorine atom onto an aromatic ring of a drug-like molecule, which is undoubtedly of great significance, has not yet been reported. Furthermore, the development of eLSF strategies for the functionalization with bioisosteres is underexplored and requires immediate consideration.
ii) The labeling or modification of peptides, proteins as well as sugars plays a key role in modern drug discovery. To date, only a limited number of electrochemical peptides/proteins LSF approaches have been disclosed, and these methods mainly focus on the modification of tyrosine or tryptophan side motifs or require prefunctionalization with oxidation-sensitive electroauxiliaries for selectivity control. Developing electrochemical strategies beyond this prefunctionalization would obviously alleviate the synthetic prospects of late-stage peptide and protein modification reactions. Meanwhile, practical eLSF of sugar derivatives remains largely elusive, which needs special attention to resolve.
iii) While a large proportion of drug molecules bear chiral structures, the enantioselective eLSF, or even the electrochemical asymmetric synthesis, has thus far been met with limited success. Hence, more efforts should be devoted toward the development of full selectivity control in asymmetric electrocatalysis.
iv) Electrode materials show great influence on electrochemical LSF reactions. While traditional electrode materials such as graphite, carbon, and metal electrodes have been extensively used in electrochemical transformations, there is a growing interest in exploring new materials with tailored properties. For instance, the development of novel electrode materials with improved catalytic activity and stability, such as modified electrodes, nanomaterials, catalyst-supported electrodes, and metal–organic frameworks (MOFs) may expand the scope of electrochemical LSF in drug discovery.
v) Electrolyte selection is crucial in electrochemical LSF reactions as it influences the reaction kinetics and efficiency. Evaluating the impact of different electrolytes, including ionic liquids or redox mediators, on reaction outcomes and exploring tailored electrolytes, including the optimization of their composition and redox potential, could broaden the applicability of electrochemistry in drug discovery.
vi) Constant current electrolysis (CCE) is the most commonly used electrochemical method. It allows for controlled reaction rates and provides simpler experimental setups, but suffers from low selectivity and competitive reactions in some cases. By contrast, controlled potential electrolysis (CPE) allows precise control over the reaction potential, enabling selective transformations, and hence deserves more attention in the eLSF arena. In addition, the recent surge of alternating current (AC) shows its meticulous control of specific reaction pathways and exact chemoselectivity. A profound comprehension of the merits and constraints of each method can aid in the development of more efficient electrochemical LSF strategies.

In summary, organic electrosynthesis has emerged as an increasingly viable and powerful platform with ideal levels of the resource economy for late-stage functionalization. We expect major advances of eLSF in drug discovery, materials science, crop protection, as well as other areas in the near future.
